# H3K4me2 ChIP-Seq reveals the epigenetic landscape during mushroom formation and novel developmental regulators of *Schizophyllum commune*

**DOI:** 10.1038/s41598-021-87635-8

**Published:** 2021-04-14

**Authors:** Peter Jan Vonk, Robin A. Ohm

**Affiliations:** Microbiology, Department of Biology, Faculty of Science, Utrecht University, Padualaan 8, 3584 CH Utrecht, The Netherlands

**Keywords:** Fungal genetics, Fungal genomics

## Abstract

Mushroom formation represents the most complex multicellular development in fungi. In the model mushroom *Schizophyllum commune*, comparative genomics and transcriptomics have previously resulted in a regulatory model of mushroom development. However, little is known about the role of epigenetic regulation. We used chromatin immunoprecipitation sequencing (ChIP-Seq) to determine the distribution of dimethylation of lysine 4 on histone H3 (H3K4me2), a mark for transcriptionally active genes, during monokaryotic and dikaryotic development. We identified a total of 6032 and 5889 sites during monokaryotic and dikaryotic development, respectively. The sites were strongly enriched near translation initiation sites of genes. Although the overall epigenetic landscape was similar between both conditions, we identified 837 sites of differential enrichment during monokaryotic or dikaryotic development, associated with 965 genes. Six transcription factor genes were enriched in H3K4me2 during dikaryotic development, indicating that these are epigenetically regulated during development. Deletion of two of these genes (*fst1* and *zfc7*) resulted in arrested development of fruiting bodies, resulting in immature mushrooms. Together these results indicate that H3K4me2 ChIP-Seq is a powerful new tool to map the restructuring of the epigenetic landscape during mushroom development. Moreover, it can be used to identify novel developmental regulators.

## Introduction

Mushroom fruiting bodies are the sexual structures of basidiomycete fungi and produce sexual spores for reproduction^1^. Mushrooms are a nutritious food source, produce pharmacological compounds and express a vast array of lignocellulolytic enzymes^2^. Their development is regulated by a complex, but largely unknown, developmental program^3–7^. Comparative genomics and transcriptomics have recently revealed many genetic elements related to mushroom development, creating a regulatory model of mushroom development^6,8–12^. However, these methods do not provide a complete overview of the restructuring of chromatin during mushroom development.

During development, the chromatin in eukaryotic genomes is generally heavily restructured to modulate gene expression by making regions of the genome either more or less accessible and by the recruitment of regulatory proteins^13^. This process is partially facilitated by modifications on the protruding tails of the histones that form the nucleosomes. These modifications can change the chemical attraction between the tail and DNA⁠ and recruit regulatory proteins to the site of modification^14,15^. There is a wide variety of histone modifications, including methylation, acetylation, ubiquitination and phosphorylation^14–16^. Among the best studied are lysine methylations of the N-terminal tail of histone H3. Methylation of the lysine 4 of histone H3 can occur as mono- (H3K4me1), di- (H3K4me2) or trimethylation (H3K4me3) and is strongly associated with transcription start sites (TSS). Therefore, H3K4 methylation is considered a reliable marker of transcription start sites^17^, although the exact distribution of mono-, di- and trimethylation around active genes can vary^18,19^.

Histones and their epigenetic modifications are highly conserved proteins due to their essential role in all eukaryotes^20^. Indeed, antibodies against histone modifications are often shown or predicted to recognize the same modification across many species and even kingdoms, including fungi. However, data on histone modification recognition by antibodies is limited to ascomycotes, including *Saccharomyces cerevisiae, Schizosaccharomyces pombe*, *Neurospora crassa*, *Fusarium graminearum* and *Zymoseptoria tritici*^21–26^. Furthermore, not all histone modifications are present in all fungal clades^27^. For example, H3K27 methylation is lost in multiple ascomycete species, including species within the Taphrinomycotina, Saccharomycotina, Eurotiomycetes and the basidiomycete *Ustilago maydis*^28,29^. In Agaricales, the presence of all H3K methyltransferases of these marks is predicted in *Coprinopsis cinerea*^27^.

Histone ChIP-Seq (chromatin immunoprecipitation followed by sequencing) is a powerful technique to map the locations of histone modifications across the genome^26,30^⁠. With this technique, antibodies against a specific histone modification are used to selectively pull-down fragmented chromatin enriched with the histone modification of interest. Next, the DNA is extracted from the chromatin, sequenced, and aligned to the reference genome. This reveals the differential enrichment of the histone modification across the genome and, by extension, the genetic accessibility of genomic regions during development.

*Schizophyllum commune* is a basidiomycete model organism for mushroom development. It has a short, well-defined lifecycle of ten days in the lab and is genetically accessible^6,31,32^. Recently, the efficiency of gene deletion was greatly increased with the use of CRISPR-Cas9, enabling more in-depth study of gene function^33^.

Here, we developed a method for histone ChIP-Seq in the model mushroom *S. commune* and mapped the distribution of H3K4me2 in the genome during monokaryotic and dikaryotic development. We chose H3K4me2, which is enriched downstream of the TSS of active genes^19^. By assessing the differential enrichment of H3K4me2 between monokaryotic and dikaryotic colonies, we were able to identify potential regulators of mushroom development. We confirmed the role of two transcription factors, *fst1* and *zfc7*, by gene deletion. This study shows that histone ChIP-Seq is a powerful technique that can further elucidate the complex developmental process of mushroom formation. To our knowledge this is the first report of the use of ChIP-Seq in a mushroom-forming basidiomycete.

## Results

### Histone H3 conservation in Fungi

**Figure 1:**
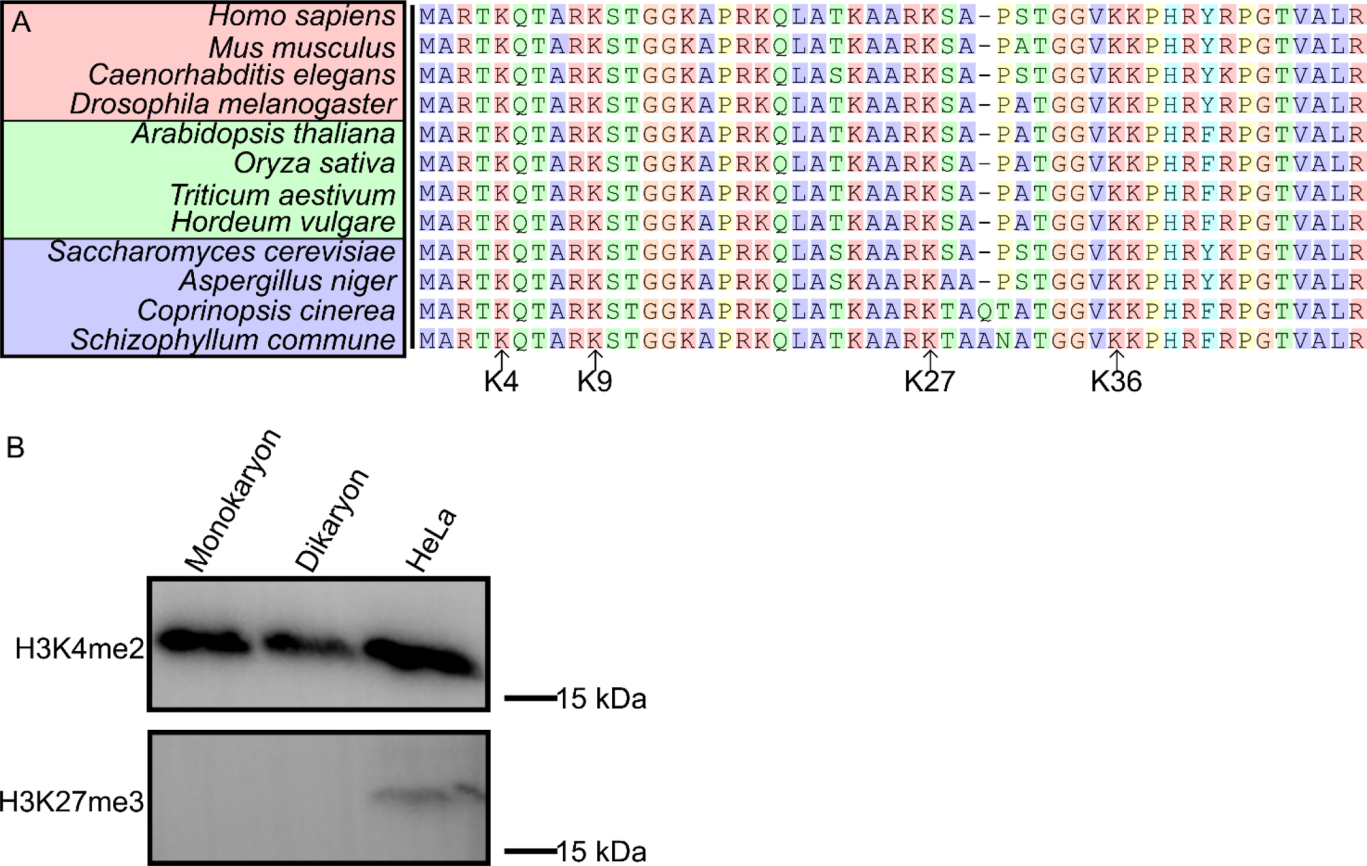
Recognition of methylation on histone H3 by mammalian anti-H3K4me2 and H3K27me3. (**A**) Sequence alignment of the 50 N-terminal amino acids of histone H3 from 12 well-studied species across the kingdoms of animalia (red), plantae (green) and fungi (blue). The four primary lysine methylation sites are indicated. Amino acids are color-coded according to the Clustal X color scheme^48^. While the lysines K4, K9, K27 and K36 are conserved across all species, immediately downstream of K27 several substitutions and an insertion have occurred in the basidiomycete fungi (*C. cinerea* and *S. commune*). This is further confirmed in a larger data set of 57 species, including 49 fungi (Supplementary Fig. 1). (**B**) Western blot for H3K4me2 and H3K27me3 on a monokaryon and dikaryon of 88 h old and HeLa whole cell lysate. The presence of the bands indicates that H3K4me2 can be detected by an antibody raised against human histone H3 with lysine 4 dimethylation, while H3K27me3 cannot be detected by an antibody raised against human histone H3 with lysine 27 trimethylation. The full-length blots of H3K4me2 and H3K27me3 are represented in Supplementary Figs. 2 and 3, respectively.

While the presence of H3K4, H3K9, H3K27 and H3K36 methylation is predicted in the Agaricales^27^, it is currently not known if these are sufficiently conserved for recognition with commercially available mammal-derived antibodies. To assess the conservation of the amino acid sequence surrounding the methylation sites, we aligned the first 50 amino acid residues of histone H3 of several commonly studied species. The N-terminal tail is highly conserved across all the studied species, but immediately downstream of H3K27 there are the substitutions S28T and P30N and the insertion A29_P30insA (i.e., an A was inserted between the A at position 29 and the P at position 30, relative to the human histone H3 sequence) that are unique to *S. commune* (Fig. 1a)*.* Similar changes are observed for the basidiomycete *C. cinerea*, but not for the ascomycetes *S. cerevisiae* and *Aspergillus niger*. This suggests that these changes are unique to basidiomycetes. Indeed, a larger data set of 49 fungal genomes showed variability surrounding H3K27 in basidiomycetes, but not ascomycetes (Supplementary Fig. 1).

A western blot confirmed that an antibody raised against human H3K4me2, but not H3K27me3, was able to detect histone H3 in *S. commune*, while both were able to detect histone H3 in a human protein extract (Fig. 1b, Supplementary Figs. 2 and 3)*.* This strongly suggests that H3K4me2 is sufficiently conserved between mammals and *S. commune* for the antibody to be used in *S. commune*. However, this is not the case for H3K27me3, which suggests that either the modification is not present in *S. commune*, or an antibody raised against human H3K27me3 is unable to recognize the region due to significant mutations. Based on these results, we decided to use H3K4me2 to develop a ChIP-Seq protocol in *S. commune*.

### Histone ChIP-Seq on H3K4me2 reveals restructuring of euchromatin during development

**Figure 2:**
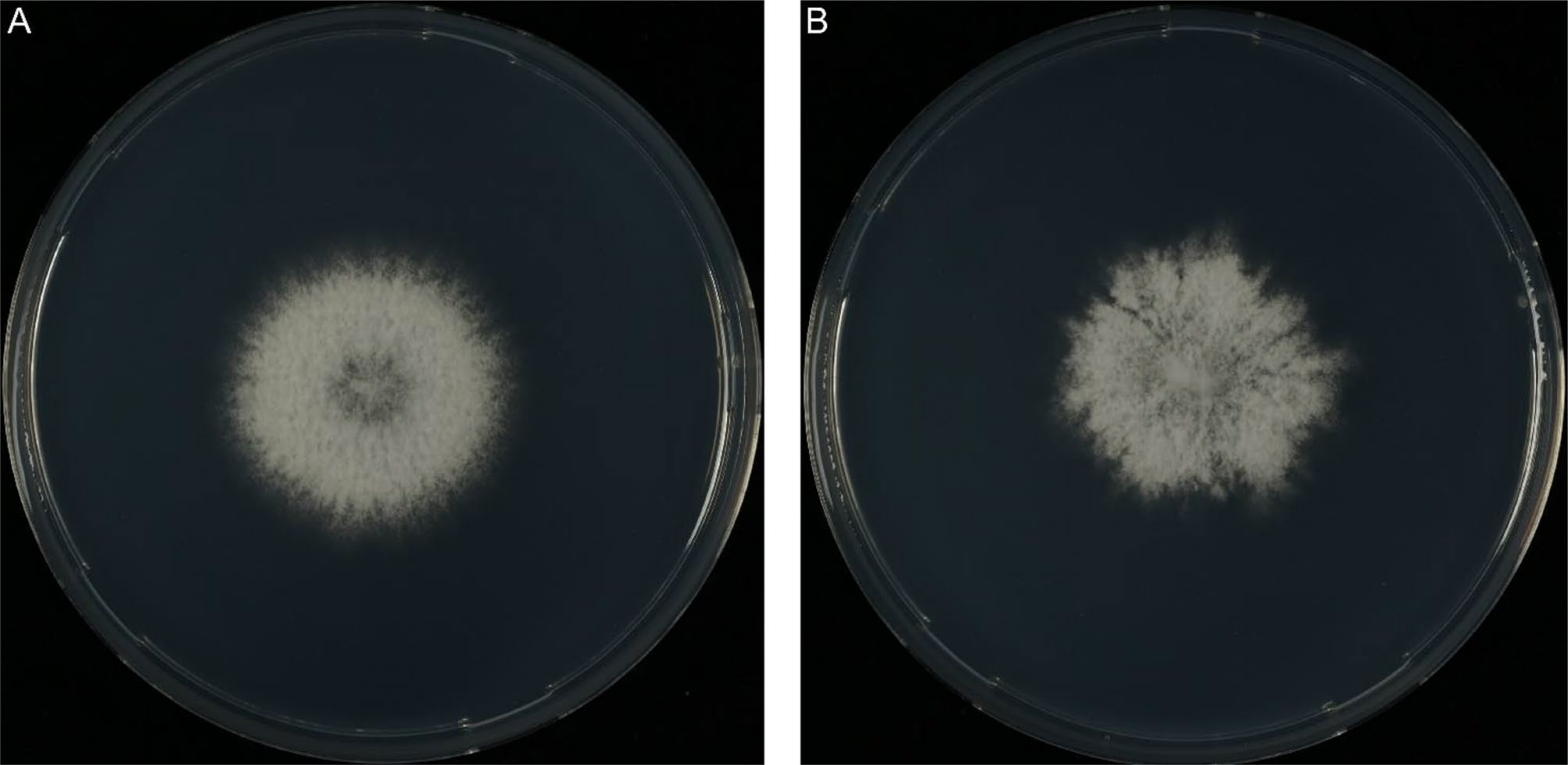
Monokaryotic (**A**) and dikaryotic (**B**) growth of *S. commune* H4-8A after 90 h. The monokaryon grows symmetrically, while the dikaryon is asymmetric and develops hyphal aggregates (the first stage of mushroom development) at the edge of the colony.

Chromatin immunoprecipitation sequencing was performed on biological duplicates of 4-day old monokaryotic (haploid) and dikaryotic colonies grown at 30 °C in a 16/8 h day/night cycle. The developmental stage of these cultures was (symmetric and homogenous) vegetative growth and the formation of secondary hyphal knots, respectively (Fig. 2a,b). The transition from vegetative monokaryotic growth to secondary hyphal knots is an important stage in the developmental process of mushrooms.

The ChIP pull-down was performed with an anti-H3K4me2 antibody, and the resulting DNA was sequenced and aligned to the genome assembly (a detailed version of the protocol can be found in Supplementary Text 1). Between 9.6 million and 21.3 million (paired-end) 75 bp reads were obtained per sample (Table 1), resulting in an average coverage ranging from 18.7 to 41.6-fold. After removing low quality reads, unaligned reads and optical duplicates, between 9.0 and 19.4 million reads remained, with 6–12.5% reads discarded and a coverage ranging from 17.5 to 37.8-fold. The percentage of unaligned reads and optical duplicates was similar between samples and input controls.

**Table 1:** Sequencing read counts for the samples in this study. Masked reads are after filtering on paired-end aligned reads that are unique and not identified as optical duplicates.

Sample	Raw read count	Average sequencing depth (fold coverage)	Read count after filtering	Kept reads (%)	Average sequencing depth after filtering (fold coverage)
Monokaryon Control 1	13,832,046	26.9	12,104,966	87.5	23.4
Monokaryon Control 2	15,463,640	30.1	14,128,322	91.4	27.5
Dikaryon Control 1	21,339,686	41.6	19,391,294	90.9	37.8
Dikaryon Control 2	17,846,656	34.8	16,264,212	91.1	31.7
Monokaryon Sample 1	9,598,100	18.7	8,973,684	93.5	17.5
Monokaryon Sample 2	1,576,8176	30.7	14,078,416	89.3	27.4
Dikaryon Sample 1	1,454,0244	28.3	13,356,868	91.9	26.0
Dikaryon Sample 2	1,394,7854	27.2	13,115,136	94.0	25.5

**Figure 3:**
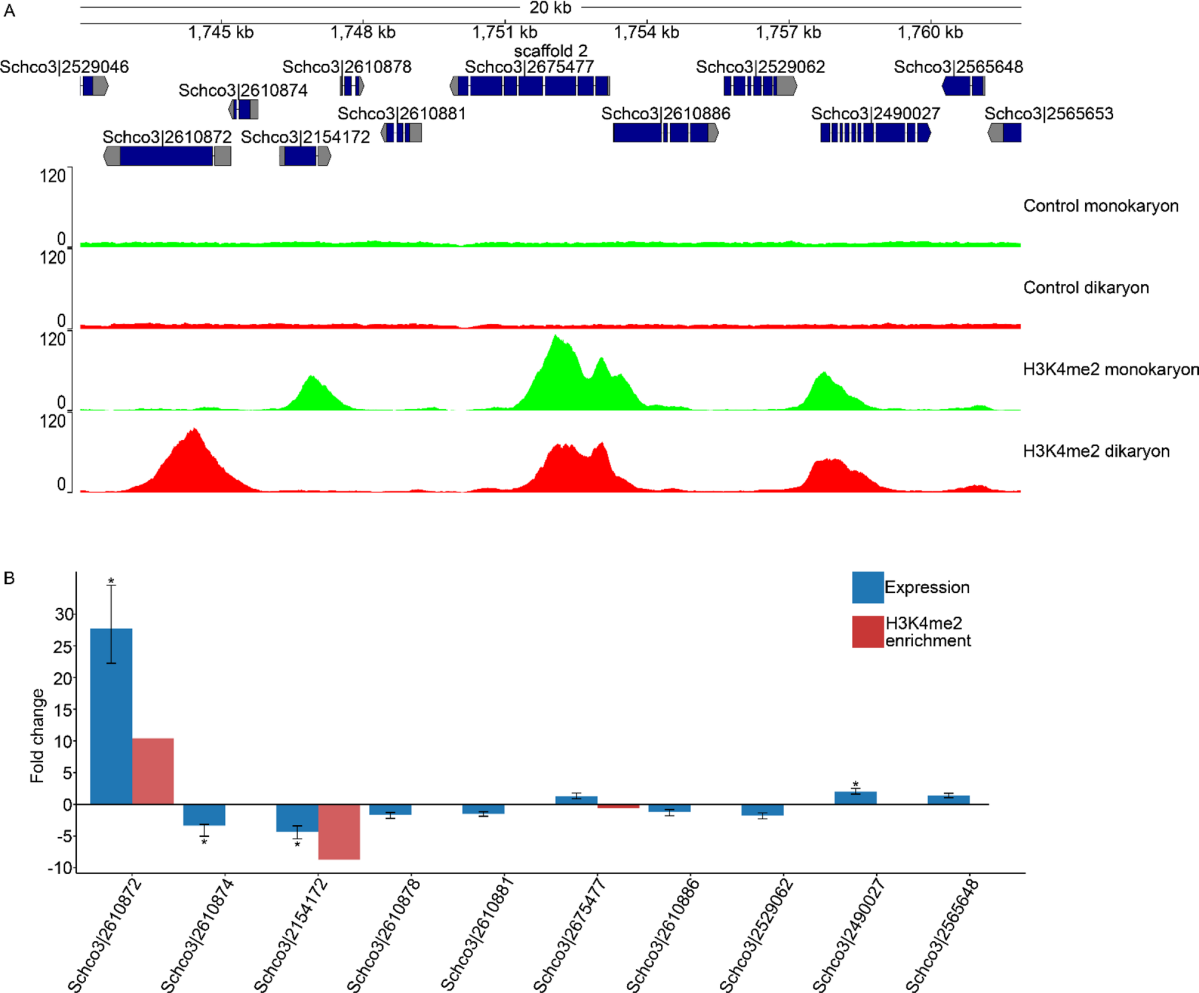
Histone H3 K4 dimethylation distribution and expression during monokaryotic and dikaryotic development on scaffold 2. (**A**) Overview of read-depth from both the control samples and the H3K4me2 ChIP samples on a representative part of the genome (scaffold 2: 1,742,000–1,762,000). The controls show a homogenous read depth across the genome, while ChIP resulted in enrichment around the translation initiation site (TIS) of genes. Most sites of enrichment were found during both monokaryotic and dikaryotic development, while some were unique to each developmental condition. (**B**) Change in expression (blue) and H3K4me2 enrichment (red) of 10 genes on scaffold 2. Positive numbers indicate higher expression and enrichment during dikaryotic growth, while negative numbers indicate higher expression and enrichment during monokaryotic growth. Asterisks (*) indicate a > twofold absolute change in expression.

In general, the input controls (in which no immunoprecipitation was performed) displayed similar read coverage along the genome, whereas the immunoprecipitated samples were concentrated in peaks that represent the binding sites of histone 3 with dimethylation on lysine 4 (Fig. 3a). As expected, within input controls and within ChIP-samples there was a high correlation (> 0.93), while the correlation between the input controls and the ChIP-samples was low (< 0.10) (Supplementary Fig. 4).

**Figure 4:**
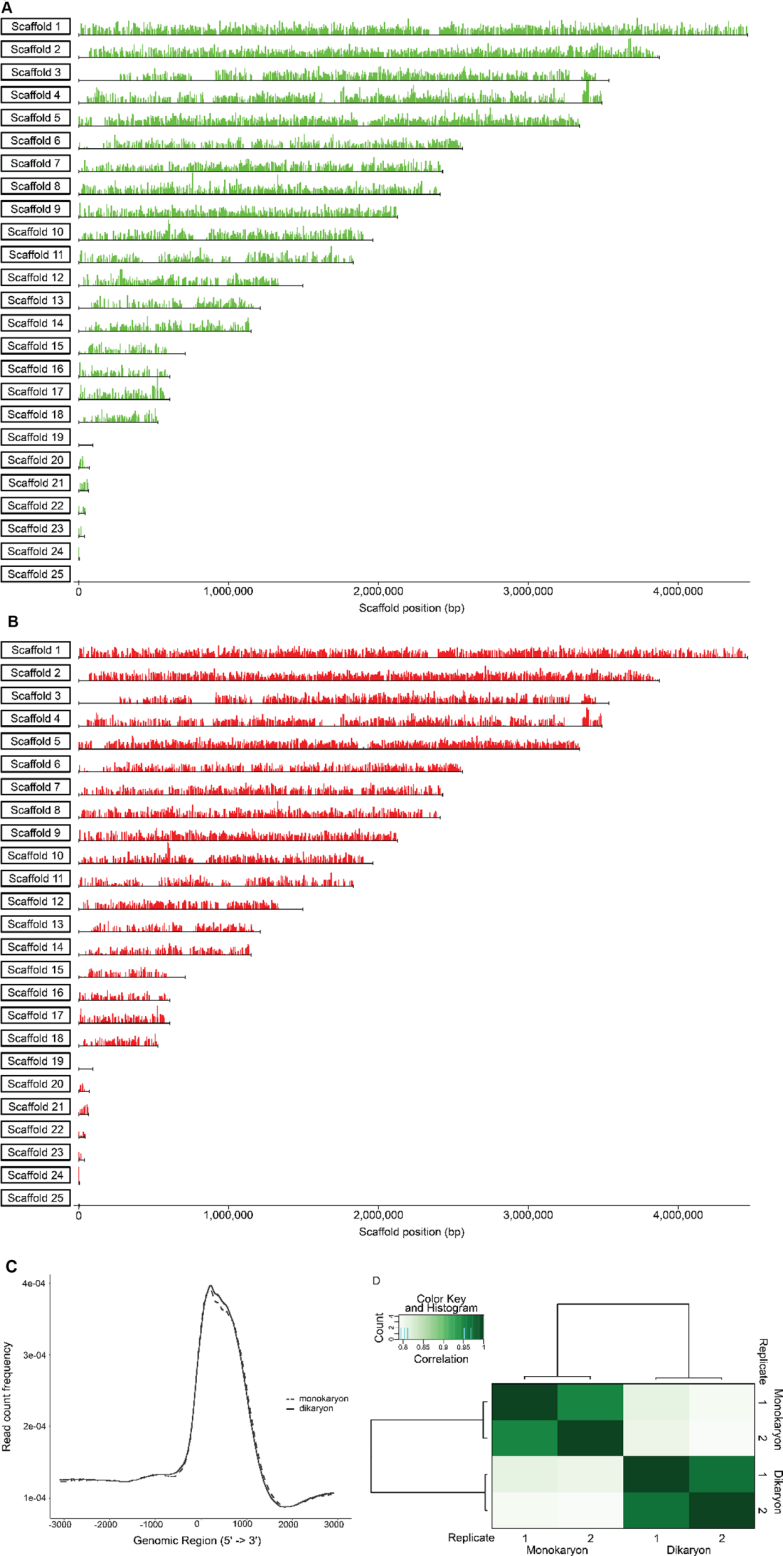
H3K4me2 peak distribution during monokaryotic and dikaryotic development. (**A**) Distribution of H3K4me2 across the *S. commune* genome during monokaryotic development. (**B**) Distribution of H3K4me2 across the *S. commune* genome during dikaryotic development. (**C**) Read count frequency 6000 bp around the translation initiation site (TIS) of genes with a peak in the monokaryon (dotted line) and dikaryon (continuous line) samples. The enrichment ranges from approximately 375 bp upstream to 1500 bp downstream of the TIS, with a summit 100 bp downstream of the TIS. The distribution of both monokaryotic and dikaryotic samples was very similar. (**D**) Correlation between samples and replicates obtained from H3K4me2 ChIP-Seq. The correlation between replicates of the same sample was > 0.95, while the correlation between the monokaryon and dikaryon samples was around 0.8. This indicates considerable conservation of the H3K4me2 landscape across the developmental stages.

Peaks were called for all samples with MACS2^34^, resulting in 6032 and 5889 significant peaks in the monokaryotic and dikaryotic colonies, respectively (Table 2). These peaks were distributed evenly across the genome (Fig. 4a,b). The fraction of reads within peaks ranged from 0.85 to 0.9. These peaks tend to locate near the start of genes (Fig. 3a). To analyze the location of the peaks with respect to the genes in more detail, we associated each peak with the closest gene [with a maximum of 3 kb from the translation initiation site (TIS)]. The coverage depth of the reads was plotted with respect to the TIS of these genes (Fig. 4c), showing an enrichment from about 375 bp upstream to 1500 bp downstream of the TIS. This pattern was almost identical in the monokaryon and dikaryon, with a Pearson correlation coefficient of > 0.99.

**Table 2:** Summary of peaks, differential peaks and associated genes for H3K4me2 ChIP during monokaryotic and dikaryotic development.

Condition	Peaks	Differential peaks	Peak-associated genes
Monokaryon	6032	667	771
Dikaryon	5889	170	194

Next, we compared the peaks between the two conditions, since a difference in the peaks indicates a difference in binding affinity of histone 3 (and therefore the epigenetic regulation) of that locus. A differential peak analysis with DiffBind^35^ revealed a correlation of 0.95 and 0.97 within replicates for the monokaryotic and dikaryotic samples and a correlation of 0.79–0.81 between the conditions (Fig. 4d). This indicates that most of the monokaryotic genome architecture associated with histone 3 methylation (H3K4me2) is maintained in the initial stages of dikaryotic mushroom development. A total of 837 peaks were identified that displayed a difference between the conditions, with 667 enriched in the monokaryotic colonies and 170 in the dikaryotic colonies (Table 2).

These relatively enriched peaks in the monokaryon and dikaryon were associated with the gene(s) they presumably regulate. Based on the general pattern in Fig. 4a, we associated a peak with a gene if it was between 375 bp upstream and 1500 bp downstream of the TIS. This resulted in 771 and 194 genes that were specifically enriched in the monokaryotic and dikaryotic developmental stages, respectively (Supplementary Table 1). A functional annotation enrichment analysis of these genes revealed an over-representation of the PFAM domains PF05920 (homeodomain transcription factors) and PF13476 (ATPase-related proteins) in genes near peaks that were specific for monokaryotic growth. PF05920-annotated genes are homeodomain transcription factors and functional annotation revealed 5 of 6 genes were part of the *mat*A mating type locus. However, due to the variation in mating type loci between compatible strains, this enrichment is likely an artefact of alignment to the reference genome, which only contains the mating type loci of *mat*A43^36^. Therefore, the mating type loci were excluded from the enrichment analysis. No PFAM domains were significantly enriched in the dikaryotic samples.

To verify that H3K4me2 enrichment correlated with gene expression, we performed RT-qPCR on the 10 genes in a 20 kb region on scaffold 2 from 1,742,000 to 1,762,000 (Fig. 3a,b). Of these genes, 4 were differentially regulated (twofold cut-off): Schco3|2610874 and Schco3|2154172 had a higher expression during monokaryotic development of 3.3 and 4.3-fold, respectively, and Schco3|2610872 and Schco3|2490027 had a higher expression during dikaryotic development of 27.7 and twofold, respectively. The two strongest differentially regulated genes, Schco3|2154172 and Schco3|2610872, also had the strongest differential ChIP-Seq peak size of 8.7 and 10.4, respectively (Fig. 4c and Supplementary Table 1). Schco3|2675477, which had a stronger enrichment of H3K4me2 during monokaryotic development (0.64) did not have a significant increase in expression during monokaryotic development, suggesting that small differences in H3K4me2 enrichment do not necessarily lead to differential expression.

A total of 25 transcription factors were associated with enriched peaks in the monokaryon, while six were associated with enriched peaks in the dikaryon (Table 3). Among the transcription factors identified in the monokaryon was *pri2*, a previously studied zinc-finger transcription factor with no known function in *S. commune*^31^. The six transcription factors identified in the dikaryon included five fungal specific transcription factor zinc fingers and one C2H2 zinc finger. None of these transcription factors have been previously studied in any mushroom-forming species.

**Table 3:** Transcription factors with differential enrichment of H3K4me2 during monokaryotic and dikaryotic development.

ProteinId	Condition	Transcription factor type
Schco3|1157466	Monokaryon	bZIP
Schco3|2645246	Monokaryon	bZIP
Schco3|2717189	Monokaryon	C2H2 zinc finger
Schco3|2642040	Monokaryon	Forkhead
Schco3|2493743	Monokaryon	Fungal specific TF
Schco3|2525437	Monokaryon	Fungal specific TF
Schco3|2611855	Monokaryon	Fungal specific TF
Schco3|2620175	Monokaryon	Fungal specific TF
Schco3|2624428	Monokaryon	Fungal specific TF
Schco3|2628620	Monokaryon	Fungal specific TF
Schco3|2631700	Monokaryon	Fungal specific TF
Schco3|2633058	Monokaryon	Fungal specific TF
Schco3|2635378	Monokaryon	Fungal specific TF
Schco3|82290	Monokaryon	Fungal specific TF
Schco3|2181177	Monokaryon	Helix-loop-helix
Schco3|2045557	Monokaryon	HMG
Schco3|2467772	Monokaryon	HMG
Schco3|2517503	Monokaryon	HMG
Schco3|2539542	Monokaryon	HMG
Schco3|2662380	Monokaryon	HMG
Schco3|2686461	Monokaryon	HMG
Schco3|2603970	Monokaryon	Homeodomain
Schco3|2613044	Monokaryon	Homeodomain
Schco3|2625693	Monokaryon	MADS-box
Schco3|2585708	Monokaryon	Zinc finger, PARP-type
Schco3|2601101	Dikaryon	C2H2 zinc finger
Schco3|2351130	Dikaryon	Fungal specific TF
Schco3|2495705	Dikaryon	Fungal specific TF
Schco3|2502848	Dikaryon	Fungal specific TF
Schco3|2577799	Dikaryon	Fungal specific TF
Schco3|2641534	Dikaryon	Fungal specific TF

### Deletion of two dikaryon-specific transcription factors results in incomplete fruiting

**Figure 5:**
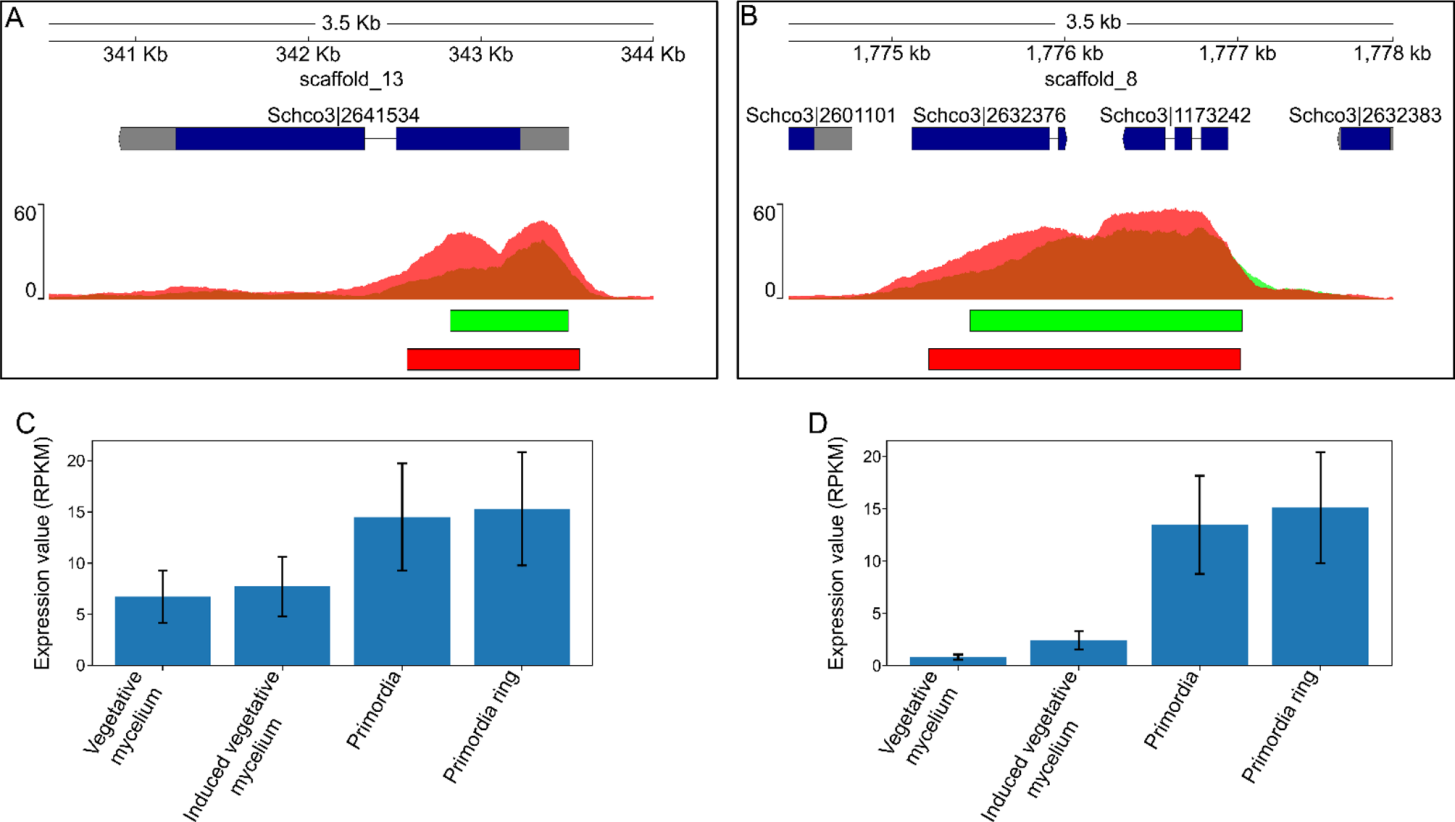
Read depth and expression of the transcription factor genes *fst1* (protein ID Schco3|2601101) and *zfc7* (protein ID Schco3|2601101). (**A**,**B**) The normalized read depth of H3K4me2 ChIP during monokaryotic (green) and dikaryotic (red) development in the loci of *fst1* (**A**) and *zfc7* (**B**). Overlap in read depth is indicated in orange. The identified peaks are indicated below. Both *fst1* and *zfc7* have increased read depth and broader peaks during dikaryotic development. (**C**,**D**) Expression of *fst1* (**C**) and *zfc7* (**D**) during mushroom formation in *S. commune*^6^. The 95% confidence interval is indicated by black error bars. Both transcription factors have increased expression during primordia formation.

We selected two transcription factors, *fst1* (ProteinID: 2641534) and *zfc7* (ProteinID: 2601101), named after the fungal-specific transcription factor (PF04082) and zinc finger C2H2 (PF00096) domains, respectively. Both genes had nearby peaks that were enriched during dikaryotic growth (specifically, primordia formation) when compared to monokaryotic growth (Fig. 5a,b). This profile suggested that their role takes place during primordia formation. Their expression profile also suggested a role in primordia development, as expression increased in the primordia compared to vegetative growth (Fig. 5c,d)^6^. Furthermore, both *fst1* and *zfc7* had higher expression during dikaryotic development than during monokaryotic development (as determined by quantitative RT-PCR), with a relative expression increase of 2.0 and 14.8-fold, respectively. To study their role, the genes were deleted in a *Δku80* strain by homologous recombination with a plasmid containing a nourseothricin resistance cassette flanked by 1 kb upstream and downstream flanks of the target gene. To increase efficiency, Cas9 preassembled with two sgRNAs against the gene of interest were co-transformed^33^. Candidate gene deletions were selected on nourseothricin and counter-selected on phleomycin. Gene deletions were confirmed by colony PCR (Supplementary Fig. 5) and selected knockout strains were crossed with the compatible isogenic H4-8b to obtain compatible monokaryons of the deletion strains with an intact *ku80* gene. To determine if phenotypes could be attributed to the deleted genes, both genes were complemented by ectopic integration of the genes under the control of the endogenous promoter and terminator. Candidate complemented strains were crossed with a compatible deletion strain to assess the recovery of wild type fruiting body formation (Supplementary Fig. 6).

**Figure 6:**
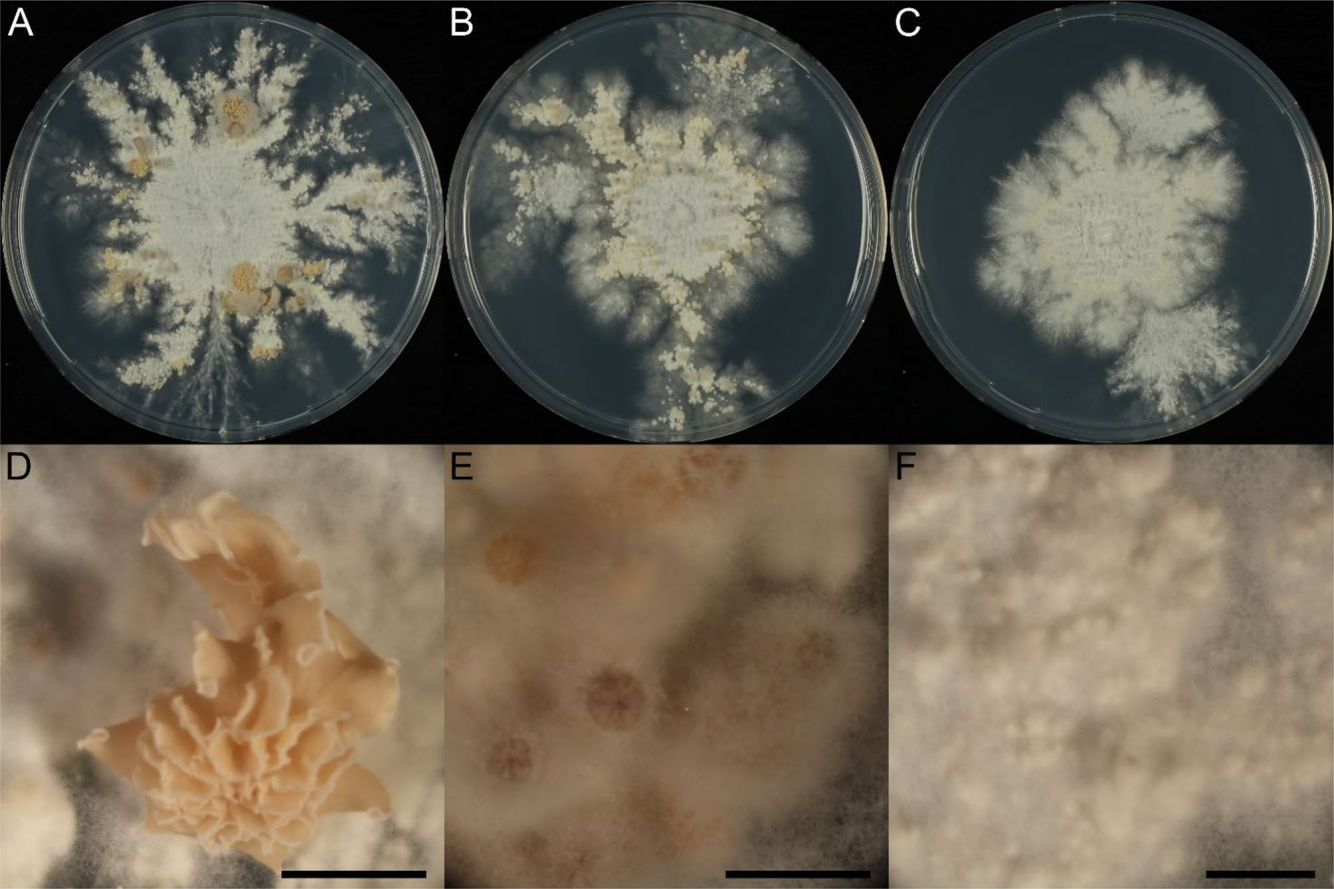
Mushroom development in wild type (**A**,**D**), *Δfst1* (**B**,**E**) and *Δzfc7* (**C**,**F**) dikaryons. (**A**–**C**) Represent full colonies and (**D**–**F**) represent magnifications of the fruiting bodies. The scale bars represent 2.5 mm.

Vegetative growth in monokaryons was not altered by deletion of either *fst1* or *zfc7* after seven days (data not shown). However, both strains showed stunted primordia and mushroom formation (Fig. 6a–f) when grown under mushroom-inducing conditions. While asymmetric growth was established after three days, brown pigmentation and aggregate formation was reduced in the primordia ring. After seven days, wild type dikaryons formed mature mushrooms with opened gills, while homozygous *Δfst1* dikaryons produced cup-shaped mushrooms covered with aerial hyphae and reduced brown pigmentation. Homozygous *Δzfc7* dikaryons formed some spots of immature fruiting bodies, but most of the primordia halted development after aggregate formation and showed reduced brown pigmentation.

## Discussion

Comparative genomics and transcriptomics have become commonplace to study the development of mushroom-forming fungi^5,6,8,9,12^. These approaches have provided valuable insight into the developmental plan of fruiting body formation. However, little is known about the underlying epigenetic changes during development. Here, we used ChIP-Seq to fill the gap between the genome of *S. commune* and the transcriptomic changes during development, by targeting the conserved activating histone mark H3K4me2 during the initial stages of monokaryotic and dikaryotic development. The H3K4me2 sites were strongly enriched around the translation initiation site (TIS). We used the resulting data to identify two genes predicted to play a role in the early stages of primordia formation. Indeed, homozygous deletion strains of either *fst1* or *zfc7* were unable to form mature mushrooms, but instead stopped their development in the primordia stage. This indicates that H3K4me2 enrichment may serve as a marker for the identification of candidate genes involved in processes downstream of the time of sampling.

There are no previous studies on the distribution of H3K4me2 in mushroom-forming fungi, or any other basidiomycete. However, the distribution we observed downstream of the TIS is similar to those observed in ascomycetes, including *Saccharomyces cerevisiae* and *Z. tritici*^26,37,38^. In some plant-pathogenic species histone modifications can be chromosome-specific due to the presence of accessory chromosomes with low gene content^25,39^. We did not observe such a distribution in the genome of *S. commune*, due to the absence of accessory chromosomes. While in general H3K4me2 is associated with active genes^19,39^, we did identify genes previously associated with dikaryotic development that were enriched with H3K4me2 during monokaryotic development. This includes *pri2*, which is strongly upregulated during mushroom-formation and is implicated in dikaryotic development in *Lentinula edodes*^6,9,31,40^. Recently, H3K4 methylation has also been associated with repression of specific gene clusters in *S. cerevisiae*, which can perhaps explain the methylation profile of *pri2*, although a better understanding of histone modifications in *S. commune* is required to confirm this^41^.

While epigenetic histone marks are generally strongly conserved across eukaryotes^20^, we found that significant changes in the protein sequence of histones may limit our ability to probe certain histone marks in *S. commune* with commercially available antibodies*.* Therefore, we were unable to confirm the presence of inhibitory H3K27me3 histone mark in *S. commune.* While this is likely due to amino acid substitutions and insertions surrounding lysine 27 on histone H3, it is also possible that H3K27 methylation is not conserved in *S. commune.* It has previously been shown that H3K27 methylation does not occur in multiple ascomycetes, including several *Aspergillus* species and yeasts, due to the absence of KMT6 homologs^21,22,27–29^. In the basidiomycete yeast *Cryptococcus neoformans* H3K27 methylation was previously detected, and in the mushroom-forming species *C. cinerea* H3K27 methylation is predicted based on presence of a KMT6 ortholog^27,42^. Other basidiomycete species, like *Ustilago maydis*, do not have a KMT6 ortholog, which suggests a loss off function in some species.

With H3K4me2 ChIP-Seq we observed a correlation of 0.95–0.97 within replicates and 0.79–0.81 between monokaryotic and dikaryotic samples. This indicates that the majority of the H3K4me2 landscape is very similar between these samples. This is in line with the relatively low number of differential peaks we identified. Additionally, the distribution around the TIS is strongly correlated between both developmental stages. In human cell lines, it has previously been described that the distribution and location of H3K4me2 can change drastically during development^18,43,44^. Data on more histone modifications during additional stages of development should increase our understanding of the underlying chromatin modifications regulating gene expression in mushroom-forming fungi. Moreover, deletion of histone methyltransferase genes followed by ChIP-Seq may result in more insight into their impact on the epigenetic landscape during development.

Despite the large degree of similarity in the H3K4me2 landscape between monokaryotic and dikaryotic life stages, there were also differences. Surprisingly, more genes had enrichment of H3K4me2 during monokaryotic development than during dikaryotic development, with 771 and 194 genes identified, respectively. Canonically, mushroom development is considered the most complex developmental program of mushroom-forming fungi, but our data suggests that this is not directly reflected in this type of epigenetic regulation^1,7^. However, in our approach we sampled the entire colony of a monokaryotic and dikaryotic culture. Likely, the chromatin is constantly remodeled during mushroom development, leading to different patterns of chromatin regulation in various parts of the colony. Therefore, sampling a full dikaryotic colony likely hides some of this complexity by averaging out these differences. Having developed this technique, we can now sample individual developmental stages to accurately determine their epigenetic profile.

We have developed an efficient ChIP-Seq protocol for *S. commune*. Furthermore, we have shown that this approach can be used to identify novel developmental regulators. To our knowledge this is the first report of the use of ChIP-Seq in a mushroom-forming basidiomycete, and it should be straightforward to adapt it to other mushroom-forming fungi. Moreover, it opens the door to performing ChIP-Seq on specific transcription factors, allowing us to determine their binding dynamics in the genome. Combined, these techniques will further reveal the important layer of epigenetic regulation during mushroom development and other processes. Therefore, the important addition of the study of epigenetic regulation to the molecular toolkit of *S. commune* will facilitate the functional characterization of the genome, leading to important new insights into the biology of this important group of fungi.

## Materials and methods

### Culture conditions and strains

*Schizophyllum commune* was grown from a small agar inoculum on solid *Schizophyllum commune* minimal medium (SCMM) supplemented with 1.5% agar at 30 °C^45^. All strains are derived from *S. commune* H4-8 (*mat*A43*mat*B41; FGSC 9210) and the compatible isogenic strain H4-8b (*matA41matB43)*^32,36^. For deletions, a previously published *Δku80* strain was used^31^. For selection on nourseothricin (Bio-Connect, Netherlands) or phleomycin (Bio-Connect, Netherlands), SCMM was supplemented with 15 μg/mL and 25 μg/mL antibiotic, respectively^46^. For phenotypical characterization, strains were grown for 7 days at 25 °C in a 16/8 h day/night cycle.

### Histone H3 alignment

Histone H3 was identified in each organism based on the best blast-hit with *hht1* from *S. cerevisiae*. The sequences were aligned with MAFFT 7.307 and coloured according to the Clustal X colour scheme^47,48^. A species tree was constructed based on 181 highly conserved single copy genes determined by BUSCO v2 (dataset ‘fungi_odb9’)^49^. The sequences were concatenated and aligned with MAFFT 7.307^47^ and well-aligned positions were selected with Gblocks 0.91b^50^. This resulted in 61723 positions per species and from this a tree was calculated with FastTreeMP^51^. Animal and plant species were added manually based on the NCBI taxonomy. The final tree and alignments were visualized with the interactive Tree Of Life v4^52^. The branch lengths are not drawn to scale.

### Western blot

*Schizophyllum commune* was grown as a monokaryon or dikaryon for 4 days in a 16/8 h day/night cycle on porous polycarbonate (PC) membranes (diameter 76 mm; pore size 0.1 µm; Osmonics; GE Water Technologies, PA, USA). After 90 h, half colonies were harvested and homogenized in a TissueLyser II (Qiagen, Germany) with 2 grinding beads in 2 mL Eppendorf Safe-Lock tubes (Eppendorf, Germany) at 25 Hz for 2 min. The homogenate was resuspended in 500 µL cell lysis buffer (20 mM Tris–HCl pH 8.0, 85 mM KCl, 0.5% IGEPAL CA-630 (Sigma, MO, USA), 1 × cOmplete protease inhibitor cocktail (Roche, Switzerland) and incubated for 10 min at 4 °C. The samples were centrifuged for 5 min at 2500 *g* at 4 °C and the pellet was resuspended in 250 µL nuclei lysis buffer (10 mM Tris–HCl pH 7.5, 1% IGEPAL CA-630, 0.5% sodium deoxycholate, 0.1% SDS, 1 × cOmplete protease inhibitor cocktail). The samples were incubated at 4 °C for 10 min and cellular debris was removed by centrifugation for 10 min at 15,000 *g* at 4 °C. As a positive control, 45 µg of HeLa whole cell lysate was used (ab29545, Abcam UK). All samples were mixed with 250 µL 2 × Laemmli sample buffer (4% SDS, 20% glycerol, 10% β-mercaptoethanol, 0.004% bromophenol blue, 125 mM Tris–HCl pH 6.8) and incubated at 100 °C for 10 min. The protein extracts were size-separated by SDS-PAGE on Mini-PROTEAN TGX Stain-Free Precast Gels (Bio-Rad, CA, USA) according to the manufacturer’s specifications and transferred to polyvinylidene difluoride membrane (ThermoFisher Scientific, MA, USA) according to the manufacturer’s specification. The membrane was blocked with 5% bovine serum albumin (Sigma-Aldrich, MO, USA) in phosphate buffered saline supplemented with Tween (PBS-T) (137 mM NaCl, 10 mM Na_2_HPO_4_, 1.8 mM KH_2_PO_4_, 2.7 mM KCl, 0.1% Tween 20) for 60 min, followed by incubation with either Recombinant Rabbit Anti-Histone H3 (di-methyl K4) (ab32356, Abcam, UK) diluted 1:2000 or, rabbit anti-Tri-Methyl-Histone H3 (Lys27) (#9733, Cell Signaling Technology, MA, USA) diluted 1:1000 in PBS-T with blocking buffer for 60 min. The membrane was washed 3 times with PBS-T for 5 min before incubation with an HRP-linked anti-rabbit IgG (ab205718, Abcam, UK) diluted 1:10,000 in PBS-T for 60 min. The membrane was washed 3 times in PBS-T for five minutes and imaged with Clarity ECL Substrate (Bio-Rad, CA, USA) according to manufacturer’s specification.

### Crosslinking and chromatin isolation

The chromatin immunoprecipitation method was adapted from previous studies in human cell lines and *Zymoseptoria tritici*^26,30^. See also Supplementary Text 1 for a protocol and additional considerations while performing ChIP-Seq. *S. commune* was grown as a monokaryon and dikaryon at 25 °C on porous PC membranes in a 16/8 h day/night cycle. After 90 h 5 full colonies were collected per replicate and briefly washed in Tris-buffered saline (TBS) (50 mM Tris–HCl pH 7.6, 150 mM NaCl). The samples were crosslinked by vacuum infiltration in 1% formaldehyde in TBS for 15 min and crosslinking was quenched by vacuum infiltration in 0.125 M glycine for 5 min. The crosslinked mycelium was briefly dried and frozen in liquid nitrogen. Next, the mycelium was homogenized in stainless steel grinding jars in a TissueLyser II (Qiagen, Germany) at 30 Hz for 2 min. The homogenate was resuspended in 10 mL cell lysis buffer (20 mM Tris–HCl pH 8.0, 85 mM KCl, 0.5% IGEPAL CA-630 (Sigma-Aldrich, MO, USA), 1 × cOmplete protease inhibitor cocktail (Roche, Switzerland) and incubated for 10 min at 4 °C. The samples were centrifuged for 5 min at 2500 *g* at 4 °C and the pellet was resuspended in 3 mL nuclei lysis buffer (10 mM Tris–HCl pH 7.5, 1% IGEPAL CA-630, 0.5% sodium deoxycholate, 0.1% SDS, 1 × cOmplete protease inhibitor cocktail). The soluble chromatin was fragmented by sonication for 8 min on ice, using a branson sonifier 450 with microtip at setting 4 with 35% output. The samples and the tip were cooled every 2 min during sonication. After fragmentation, the samples were incubated for 10 min at 4 °C and then spun down for 10 min at 15,000 *g* at 4 °C. The supernatant contained the isolated chromatin. 300 μL was stored at − 80 °C for input control, while the remaining chromatin was used for ChIP.

### Chromatin immunoprecipitation

The entire chromatin immunoprecipitation (ChIP) was performed at 4 °C unless stated otherwise. ChIP dilution buffer (0.01% SDS, 1.1% Triton X-100, 1.2 mM EDTA, 16.7 mM Tris–HCl pH 8.0, 167 mM NaCl, 1 × cOmplete protease inhibitor cocktail) was added to 3 mL to the chromatin suspension. For pre-clearing, 20 μL Pierce Protein A Magnetic Beads (ThermoFisher Scientifc, MA, USA) were incubated with the samples for 1 h and the supernatant was transferred to a new tube. Next, 5 μL rabbit-anti-histone H3K4me2 (ab32356, Abcam, UK) was incubated with each sample for 18 h. To each sample 20 μL of Pierce Protein A Magnetic Beads was added and the samples were incubated for an additional hour. The magnetic beads were collected and washed 1 time with low salt washing buffer (0.1% SDS, 1% Triton X-100, 2 mM EDTA, 20 mM Tris–HCl pH 8.0, 150 mM NaCl), 2 times with high salt washing buffer (low salt washing buffer with 500 mM NaCl), 2 times with lithium chloride washing buffer (250 mM LiCl, 1% IGEPAL CA-630, 1% sodium deoxycholate, 1 mM EDTA, 10 mM Tris–HCl pH 8.0) and 2 times TE buffer. During each washing step the samples were incubated for 5 min. After the first wash with lithium chloride washing buffer the samples were transferred to room temperature. Chromatin was eluted twice for 10 min in 250 μL in fresh elution buffer (1% SDS, 0.1 M NaHCO_3_). The RNA was degraded by incubating with 50 mg RNAse A (Sigma-Aldrich, MO, USA) for 1 h at 50 °C and then the samples were de-crosslinked overnight with 75 μL reverse crosslinking buffer (250 mM Tris–HCl pH 6.5, 62.5 mM EDTA, 1.25 M NaCl, 5 mg/mL proteinase K (ThermoFisher Scientific, MA, USA)) at 65 °C. The input controls were adjusted to 500 μL with water and de-crosslinked under the same conditions.

### DNA isolation

The decrosslinked DNA was purified by phenol–chloroform extraction. Briefly, 1 volume of phenol–chloroform (1:1) was added, mixed thoroughly and the aqueous phase was extracted after centrifugation at 15,000* g* for 5 min. This step was repeated a total of 3 times. The phenol was removed by repeating extraction with 1 volume of chloroform and 580 µL of the aqueous phase was transferred. The DNA was precipitated with 58 µL 3 M NaAc pH 5.6 and 1160 µL ethanol and coprecipitated with 1 µL glycogen (20 mg/mL) (ThermoFisher Scientific, MA, USA). The sample was mixed thoroughly and then stored at − 80 °C for 2 h. The DNA was collected by centrifugation at 15,000 *g* for 45 min at 4 °C and the DNA pellet was washed with 1 mL 70% ethanol. The pellet was collected by centrifugation at 15,000 *g* for 15 min at 4 °C and resuspended in 30 µL TE buffer. The DNA was further purified with the ChargeSwitch gDNA Plant Kit (ThermoFisher Scientific, MA, USA) according to the manufacturer's specifications and eluted in 50 μL ChargeSwitch elution buffer. The samples were concentrated to 25 μL using a speedvac and sent to the Utrecht Sequencing Facility (USEQ, www.useq.nl) for library preparation and sequencing.

### Library preparation and sequencing

For each sample, 80% was used for library prep with the NEXTflex Rapid DNA-Seq Kit Bundle (Bioo Scientific, TX, USA) according to manufacturer's specifications. The resulting libraries were sequenced on the Illumina NextSeq500 2 × 75 mid output platform.

### Sequencing analysis

The sequence reads were mapped to the *S. commune* H4-8 reference genome (version Schco3^36^) with bowtie2 (version 2.3.4.1)^53^. The aligned reads were filtered on unique, paired-end alignments with a quality score > 1 with samtools (version 1.7)^54^. Duplicated reads were marked with picard (version 2.21.6)^55^ and removed with samtools. Peak calling was performed by macs2 (version 2.2.3)^34^ for individual replicates and across all replicates together. The differential peaks were determined with Bioconductor Diffbind (version 2.14.0)^35^. The initial peak distribution was determined with ChIPseeker^56^ and genes were correlated to peaks based on this distribution. Final distribution was determined by identifying the closest gene to a peak within 3000 bp upstream and downstream of the TIS (translation initiation site) and calculating the relative read depth of each position around the TIS of these genes (Fig. 4a).

### Quantitative RT-PCR

*Schizophyllum commune* was grown as a monokaryon and dikaryon at 25 °C on porous PC membranes in a 16/8 h day/night cycle. After 90 h, half colonies were harvested and homogenized in a TissueLyser II (Qiagen, Germany) with 2 grinding beads in 2 mL Eppendorf Safe-Lock tubes (Eppendorf, Germany) at 25 Hz for 1 min. The homogenized mycelium was resuspended in 1 mL TRIzol reagent (ThermoFisher Scientific, MA, USA) and the RNA was isolated according to the manufacturer’s specifications and resuspended in 100 µL nuclease-free water. The RNA was cleaned up with the GeneJET RNA purification kit (ThermoFisher Scientific, MA, USA) according to manufacturer’s specification and eluted in 100 µL nuclease free water. The RNA was then quantified and 1 µg was used for input in RT-PCR with the Quantitect Reverse Transcription Kit (Qiagen, Germany) according to manufacturer’s specification. Primers for qPCR were designed across introns with a product size of 100–150 bp (Table 4). Primer efficiency was determined in duplicate with dilutions of 1:10 to 1:100,000 of cDNA from the monokaryon. All primers had an efficiency between 90 and 110%. SYBR Green chemistry (ThermoFisher Scientific, MA, USA) and an ABI Prism 7900HT SDS (ThermoFisher Scientific, MA, USA) were used for RT-PCR. Data was analyzed with the QuantStudio V1.3 software. As reference genes, *act1, tef1* and *ubi1* were used and relative quantification was calculated from an average of these genes^57^.

**Table 4:** Primers used in this study. Bold parts are overlapping regions for Gibson assembly. Underlined parts are protospacers for sgRNA.

Primer name	Sequence
2610872_qPCR_fw	CCAGGACCAGGACCAGAC
2610872_qPCR_rv	GCACATCGCTAGCACACAAC
2610874_qPCR_fw	GTCGGTGTTAGCCGAAAGTG
2610874_qPCR_rv	ATGTACGGCCTTGCGACTT
2154172_qPCR_fw	CTTGGAGCCTTCCATACTGC
2154172_qPCR_rv	CGAGGGAAGATCTTGATGGA
2610878_qPCR_fw	GCGGCTGAGGGAAGATAAAT
2610878_qPCR_rv	CGCTAATCGTGTGCAGTCTC
2610881_qPCR_fw	CGACAATCATGTCCGTGTTC
2610881_qPCR_rv	TCGCCTTCCTTACCCTTTC
2675477_qPCR_fw	AGAGGCCGAGTGAATTTTGA
2675477_qPCR_rv	CGCATCACGGACTCGTCT
2610886_qPCR_fw	CCACCAAAACCAGAGAACGA
2610886_qPCR_rv	GCCAAACAAAGCATTCAAAA
2529062_qPCR_fw	ACTCGACGATGGCCAACAC
2529062_qPCR_rv	GCATTGTTGGTCACGGAAAT
2490027_qPCR_fw	ATGCGTATGATCCCGAGAAG
2490027_qPCR_rv	CATTGCGGTCATAGTTGACG
2565648_qPCR_fw	GATGGGGCATCGCTTCTC
2565648_qPCR_rv	TCATGACCCTCTTCAACTACGA
fst1_qPCR_fw	GGCCCTCCTTCGCGTACT
fst1_qPCR_rv	AGGCGTGCGAGAAGTGTAGA
zfc7_qPCR_fw	ACGACCTCAAGCGACACC
zfc7_qPCR_rv	AGGCTTCATCGATTTTACCG
akt1_qPCR_fw	CTGCTCTTGTTATTGACAATGGTTCC
akt1_qPCR_rv	AGGATACCACGCTTGGACTGAGC
tef1_qPCR_fw	AGCTTGGCAAGGGTTCCTTCA
tef1_qPCR_rv	AACTTCCAGAGGGCGATATCA
ubi1_qPCR_fw	GAAGGAGTACGATGCGAAGG
ubi1_qPCR_rv	TCCTCCTCTGCCTTCTTGC
fst1_up_fw	**CTATGACCATGATTACGCCA**AAGACAACAGGACGCGTC
fst1_up_rv	**GTCCCCCTCGAGGCGCGCC**GTATTTGCTTGCTTGCTGCTC
fst1_down_fw	**TCCCAGACCACCATGCCGGG**TGAGCAGGCAGATGAGATC
fst1_down_rv	**GATAACCTTCAGCAGAACGA**AGGGAGGGATAGTCCTTCTC
zfc7_up_fw	**CTATGACCATGATTACGCCA**CGCAGATAACTCGAAAGCTG
zfc7_up_rv	**GTCCCCCTCGAGGCGCGCC**GAGGAGAGACTTTAGAGATCGC
zfc7_down_fw	**TCCCAGACCACCATGCCGGG**ACTCGAGACTTTGAGGTACG
zfc7_down_rv	**GATAACCTTCAGCAGAACGA**GGGAGAGTCGAACCTATGG
p1_sgRNA_fst1_left	TAATACGACTCACTATAGGGCAGCGCGACGTACTCGCG
p2_sgRNA_fst1_left	TTCTAGCTCTAAAACCGCGAGTACGTCGCGCTGCC
p1_sgRNA_fst1_right	TAATACGACTCACTATAGGAGATGCGTGGTCGTCGTAG
p2_sgRNA_fst1_right	TTCTAGCTCTAAAACCTACGACGACCACGCATCTC
p1_sgRNA_zfc7_left	TAATACGACTCACTATAGACTGGTCAGTGCGCGTATAC
p2_sgRNA_zfc7_left	TTCTAGCTCTAAAACGTATACGCGCACTGACCAGT
p1_sgRNA_zfc7_right	TAATACGACTCACTATAGCGTTAGCTCGTCAAGGGCTG
p2_sgRNA_zfc7_right	TTCTAGCTCTAAAACCAGCCCTTGACGAGCTAACG
fst1_ChkA	CAAACGAGAGCACCAGTATG
fst1_ChkB	AGCTCGTAGTACGTCATTGG
fst1_ChkC	ATCATGGTGGGTTGGGAC
fst1_ChkD	TCCAGCTTGTCCCGAGAAG
zfc7_ChkA	GTCTTCCTGTACATGCACTG
zfc7_ChkB	TCAGTGCGCGTATACTGG
zfc7_ChkC	CTAAACGAAAGGCTGGTGC
zfc7_ChkD	AGATGAAGATGAGCGAAGGG
nour_ChkB	CCGAAAGATCCGATCGATAC
nour_ChkC	CGTCATGAATGAAGCCTCAG
fst1_comp_fw	**GCGTGGCCCCAAGCGTTGGA**GACCAAACGAGAGCACCAGT
fst1_comp_rv	**AGACTGACGTGCACTCACAG**GAGGAGGCATGGTGACATTT
zfc7_comp_fw	**GCGTGGCCCCAAGCGTTGGA**TCTCGCACATCGCAGATAAC
zfc7_comp_rv	**AGACTGACGTGCACTCACAG**GATCGACCTTGCCATTGACT

### Gene inactivation

Deletion vectors of *fst1* (ProteinID: 2641534) and *zfc7* (ProteinID: 2601101) were constructed by amplifying the upstream and downstream flanks of each gene and cloned into plasmid pRO402 with a nourseothricin resistance cassette between both flanks. Plasmid pRO402 contains a pUC19 backbone with a phleomycin resistance cassette. For *fst1,* the upstream and downstream flanks were amplified with fst1_up_fw + fst1_up_rv and fst1_down_fw + fst1_down_rv, respectively, to create 1087 bp and 1025 bp fragments, respectively (Table 4). For *zfc7,* the upstream and downstream flanks were amplified with zfc7_up_fw + zfc7_up_rv and zfc7_down_fw + zfc7_down_rv, respectively, to create 1050 bp and 1007 bp fragments, respectively. All upstream PCR products contained 20 bp overlap with pRO402 and a nourseothricin cassette introduced by the forward and reverse primer respectively, while all downstream PCR products contained 20 bp overlap with a nourseothricin resistance cassette and pRO402, respectively. The upstream flank, nourseothricin resistance cassette and downstream flank were cloned into pRO402 digested with HindIII with gibson assembly (NEBuilder HiFi DNA Assembly Master Mix, New England Biolabs, MA, USA), resulting in deletion constructs pPV021 and pPV023 for *fst1* and *zfc7,* respectively. The transformation with CRISPR-Cas9 was performed as previously described with two sgRNAs per gene close to the upstream and downstream flank respectively^33^. The sgRNAs were produced with the GeneArt Precision sgRNA Synthesis Kit (ThermoFisher Scientific, MA, USA) according to manufacturer's specifications with the p1_sgRNA and p2_sgRNA primers listed in Table 4. Gene deletions were verified by PCR. For deletion strains, a primer outside the upstream (fst1_ChkA or zfc7_ChkA) and downstream (fst1_ChkD or zfc7_ChkD) and inside the nourseothricin resistance cassette (nour_ChkB and nour_ChkC) were used. As a negative control, primers inside the gene were used (fst1_ChkB, fst1_ChkC, zfc7_ChkB and zfc7_ChkC) (Table 4).

### Complementation

To complement the deletion strains, a 1000 bp promoter region and the coding sequence were amplified by PCR for *fst1* (primers fst1_comp_fw and fst1_comp_rv; Table 4) and *zfc7* (primers zfc7_comp_fw and zfc7_comp_rv; Table 4). This resulted in DNA fragments of 4207 bp and 2939 bp, respectively. All primers contained 20 bp overhangs to plasmid pPV008. This plasmid contains a pUC19 backbone, phleomycin resistance cassette a promotor and CDS insert flanked by HindIII and BamHI, an intron and a 432 bp *sc3* terminator. The fragments were assembled with pPV008 digested with HindIII and BamHI with gibson assembly. This resulted in complementation plasmids pPV024 and pPV026 for *fst1* and *zfc7,* respectively. Strains were complemented as described previously and candidate complement strains were selected based on their ability to recover the wild type fruiting phenotype^5^.

## Supplementary Information

Supplementary Information.

## Data Availability

The sequence reads are available from the Short Read Archive (SRA) of NCBI under accession ID PRJNA702885.
