# Supplementary material for: H3K4me2 ChIP-Seq reveals the epigenetic landscape during mushroom formation and novel developmental regulators of Schizophyllum commune
Source: Sci Rep. 2021 Apr 14;11:8178. doi: 10.1038/s41598-021-87635-8 (PMC8046757; doi:10.1038/s41598-021-87635-8)
Supplement: Supplementary file 1 — Supplementary Information. [file 41598_2021_87635_MOESM1_ESM.pdf]

Epigenetic landscape during mushroom formation: H3K4me2 ChIP-Seq reveals novel developmental regulators of *Schizophyllum commune*

Peter Jan Vonk, Robin A. Ohm*

Microbiology, Department of Biology, Faculty of Science, Utrecht University, Utrecht, The Netherlands
Padualaan 8, 3584 CH Utrecht, The Netherlands

* Corresponding author: r.a.ohm@uu.nl

Supplemental Text 1 - Detailed histone ChIP-Seq protocol for *Schizophyllum commune*

This protocol for ChIP-Seq in *Schizophyllum commune* is adapted from the ENCODE protocol[1] and the protocol for the ascomycete fungus *Zymoseptoria tritici*[2]. We found that neither protocol was suitable for *S. commune* without adaptation and have included notes when steps are crucial and other methods did not yield satisfactory results.

***Schizophyllum commune* culture**

Grow at least five replicate colonies of *S. commune* per condition from fresh inoculum for the preferred amount of time on SCMM-agar (22 g L^{-1} glucose monohydrate, 1.5 g L^{-1} L-asparagine monohydrate, 1 g L^{-1} K_2HPO_4, 0.5 g L^{-1} $MgSO_4 \cdot 7H_2O$, 0.46 g L^{-1} KH_2PO_4, 0.012 g L^{-1} thiamine, 0.005 g L^{-1} $FeCL_3 \cdot 6H_2O$, 1 mL L^{-1} trace elements (2 g L^{-1} $ZnSO_4 \cdot 7H_2O$, 1.2 g L^{-1} $Ca(NO_3)_2 \cdot H_2O$, 0.4 g L^{-1} $CoCl_2 \cdot 6H_2O$, 0.2 g L^{-1} $CuSO_4 \cdot 5H_2O$, 0.1 g L^{-1} $MnSO_4 \cdot 4H_2O$, 0.06 g L^{-1} HBO_3, 0.04 g L^{-1} $(NH4)_6Mo_7O_{24} \cdot 4H_2O$)) covered with a polycarbonate membrane with pores of 0.1 µm.
Note: polycarbonate membranes prevent the culture from growing into the agar, but is sufficiently porous for nutrient transport. Agar interferes with the downstream processing of samples. Particularly during sonication, where localized temperature spikes cause the agar to dissolve again.

After the required amount of time, collect colonies from polycarbonate into a 50 mL tube and wash the mycelium twice in TBS (200 mM Tris-HCl pH 6.7, 1.5 M NaCl).

Formaldehyde fixation

Add 10 mL 1% formaldehyde in TBS to the mycelium and transfer to a vacuum chamber. Apply a vacuum until the liquid no longer releases air and then remove the vacuum to infiltrate the mycelium. Repeat once and then incubate the mycelium for 10 minutes. Then remove the formaldehyde solution and quench the crosslinking reaction by adding 10 mL 0.125 M glycine. Reapply the vacuum until the liquid no longer releases air and then remove the vacuum to infiltrate the mycelium. Incubate for 5 minutes and remove the glycine.
Note: Because the mycelium is mostly intact, the liquid can be removed simply by pouring it out with the lid of the tube half open. If the mycelium is more fragmented, centrifugation is preferable.
Note: At this point the protocol can be paused by freezing the mycelium in liquid nitrogen and storing it at -80°C

Chromatin isolation

Transfer the samples in stainless steel grinding jars with a Tissuelyzer II (Qiagen, Germany) and cool the jars with liquid nitrogen. Then homogenize the samples for 2 minutes at 30 Hz.
Note: We tested chromatin isolation with small bead homogenization in the Tissuelyzer and by grinding with a mortar and pestle under liquid nitrogen as well, but had significantly reduced chromatin yield compared to homogenization with the steel grinding jars.

Transfer the homogenate to a new 50 mL tube and add 10 mL cell lysis buffer (20 mM Tris-HCl pH 8.0, 85 mM KCl, 0.5% NP-40, 1x complete protease inhibitors (Roche, Switzerland). Vortex the samples thoroughly until no clumps are left and incubate on ice for 10 minutes. Spin down

the lysed homogenate at 2500 g for 5 minutes at 4°C and discard the supernatant. Resuspend the cells in 3 mL nuclei lysis buffer (10 mM Tris-HCl pH 7.5, 1% NP-40, 0.5% deoxycholic acid, 0.1% SDS, 1x complete protease inhibitors).
Note: Most ChIP protocols for fungi do not use SDS during lysis and instead use a combination of non-ionic detergents (Triton X-100, NP-40, deoxycholic acid). We found that without SDS most chromatin would be lost during isolation, presumably due to intact nuclei.

Fragment the DNA with a sonicator. We used a Branson sonifier 450 with a microtip at setting 4 with 35% output for 4 cycles of 2 minutes. Settings for other brands should be verified experimentally by taking samples every 2 minutes and verifying fragmentation on agarose gel. For our settings, full DNA fragmentation was achieved after 8 minutes and further sonication did not reduce fragment size further (Supplemental figure S7), As an alternative micrococcal nuclease can be used to fragment the DNA. However, nucleases may introduce bias and do not yield an even distribution of chromatin size. After fragmentation store 10% (300 µL) at -80°C as input control.

Chromatin immunoprecipitation
Adjust the fragmented chromatin volume to 3 mL with ChIP dilution buffer (167 mM NaCl, 16.7 mM Tris-HCl pH 8.0, 1.2 mM EDTA, 1.1% Triton X-100, 0.01% SDS, 1x complete protease inhibitors). From this point on, always keep the samples at 4°C. Add 20 µL of protein A magnetic beads (10 mg mL^{-1}) that are washed twice in ChIP dilution buffer to each sample and incubate on a rotator at 4°C for 1 hour. Capture the magnetic beads with a magnet and transfer the supernatant to a new tube to remove any non-specific binding by protein A.
Note: At this point the solution is very viscous and the magnetic beads take a while to migrate towards the magnet. This can be alleviated by increasing the volume of ChIP dilution buffer or keeping the tubes against a strong magnet for an extended amount of time.

Add the suggested amount of antibody to the precleared chromatin and incubate on a rotator at 4°C for 18 hours. In our experiments we used 0.5 µg of antibody per ChIP. After antibody incubation, add 20 µL of protein A magnetic beads (10 mg mL^{-1}) that are washed twice in ChIP dilution buffer and incubate the samples for an additional hour at 4°C. Collect the magnetic beads with a magnet and discard the supernatant.

After collecting the magnetic bead bound chromatin, resuspend the magnetic beads in 500 µL cold low salt washing buffer (150 mM NaCl, 20 mM Tris-HCl pH 8.0, 2 mM EDTA, 1% Triton X-100, 0.1% SDS) and transfer the samples to new 2 mL tubes. Rinse the tube with an additional 500 µL of low salt washing buffer and transfer to the new tube. Subsequently wash the beads twice in 1 mL cold high salt washing buffer (500 mM NaCl, 20 mM Tris-HCL pH 8.0, 2 mM EDTA, 1% Triton X-100, 0.1% SDS), each time incubating at 4°C for 5 minutes. Next, wash the beads with 1 mL cold lithium chloride washing buffer (250 mM LiCl, 10 mM Tris-HCl pH 8.0, 1 mM EDTA, 1% IGEPAL CA-630, 1% sodium deoxycholate) and incubate at 4°C for 5 minutes. After removing the supernatant, transfer the samples to room temperature for the remainder of the washing steps. Resuspend the magnetic beats in lithium chloride washing buffer and incubate for 5 minutes. Remove the supernatant and wash twice for 5 minutes in 1 mL TE-buffer (10 mM Tris-HCl pH 8.0, 1 mM EDTA). Then elute the DNA from the magnetic beads in two steps in 250 µL freshly made elution buffer (100 mM $NaHCO_3$, 1% SDS) with constant agitation for 10

minutes. At this point, take the previously stored input control samples and adjust the volume to 500 µL with water. To remove the RNA, add 50 µg RNAse A to each sample and incubate at 50 °C for 1 hour. Then proceed with de-crosslinking by adding 75 µL reverse crosslinking buffer (1.25 M NaCl, 250 mM Tris-HCl pH 6.5, 62.5 mM EDTA, 5 mg mL^{-1} proteinase K) and incubate at 65 °C overnight.

DNA isolation

To each sample add 1 volume of phenol-chloroform (1:1), mix thoroughly, and centrifuge the samples at 15,000 g for 5 minutes. Transfer the upper aqueous phase to a new tube. Repeat this step until no white interphase is present.

Note: depending on the amount of input, this step must be repeated up to 5 times. Especially in the input control samples a lot of protein is present at the start of DNA isolation.

To remove phenol, add 1 volume of chloroform, mix thoroughly, and centrifuge the samples at 15,000 g for 5 minutes. Transfer the aqueous phase to a new tube and proceed with DNA precipitation. To each sample add 0.1 volume of 3M NaAC pH 5.6 and 2 volumes of ethanol, mix thoroughly, and incubate the samples at -80°C for 2 hours.

Note: 20 µg of glycogen can be added during this step to improve DNA precipitation and make the pellet easier to observe.

Centrifuge the samples at 4°C for 45 minutes at 15,000g and discard the supernatant carefully. Resuspend the precipitated DNA in 1 mL 70% ethanol and mix thoroughly. Collect the DNA by centrifugation at 15,000 g for 15 minutes at 4°C and discard the supernatant. Briefly dry the pellet and resuspend in 30 µL TE. Clean up the DNA further by using the isolated DNA as input in the purification method of choice.

Note: We use the ChargeSwitch gDNA plant kit from ThermoFisher Scientific for further clean-up, as S. commune produces a lot of polysaccharides that contaminate the gDNA and are not eliminated by traditional purification methods, including phenol-chloroform extraction and silica-based methods. Early ChIP-qPCR trials without additional purification did not yield reproducible results, which was alleviated by DNA cleanup.

ChIP-qPCR

To determine if the ChIP DNA is enriched compared to the input control qPCR can performed at regions expected to be enriched with the modification of choice and regions that are expected not to be enriched. Compare the ΔCt of the positive and negative regions in both input control samples and the ChIP samples. The difference in ΔCt (ΔΔCt) between the input control and ChIP samples is a measure for enrichment.

Note: We used qPCR on the region near the translation initiation site of actin, β-tubulin and gpd as regions with expected enrichment. Negative regions were selected that showed no expression nearby in any RNA-Seq data of S. commune. Specifically: scaffold 10: 864,508-864,623 (region A); scaffold 12: 8,012-8,112 (region B); scaffold 19: 17,635-17,749 (region C). For each region we found a 30-37-fold enrichment of active regions compared to regions with no expression (Supplemental figure S8).

Primers used:

Target	*Forward primer*	*Reverse primer*
actin	*CCTCTCTTCCGCGTATTCTG*	*CGTTGGCTATTCGTGAAAAA*
β-tubulin	*TGTTCACGACCTTTCCTTCC*	*GATACGATGCGACCACGAC*
gpd	*TGTGTTCATCCCGTTTTGTC*	*AAGTGCTCTGGATGGGAGTG*
region A	*CCCGGTGTCTGGTACTGACT*	*CCGTCCGAATCCGTATACAT*
region B	*TTGCTCAAAGTCGTCAATGC*	*AGCTCGCAAAACGCAGTAAT*
region C	*CGTCCTGAGAAGCTTCAACC*	*ACGTCGGCTTGAAAAAGAGA*

Library preparation and sequencing

The libraries were generated with the NEXTflex Rapid DNA-Seq Kit Bundle (Bioo Scientific, TX, USA) according to manufacturer's specifications. The resulting libraries were sequenced on the Illumina NextSeq500 2x75 mid output platform. During library preparation, the ChIP DNA can be amplified by PCR to obtain a suitable amount for sequencing. However, this may introduce bias and impact the significance of data. It is therefore recommended to increase the amount of input material when possible. Furthermore, it is strongly recommended to use paired-end sequencing, as the algorithm for peak prediction can use this information to determine the average fragment-length. This improves peak detection and results in more reproducible data.

Sequencing analysis

Reads can be trimmed using the standard tools and aligned with Bowtie2[3]. We used the sensitive-local option for alignment. Next, filter the reads based on paired-end alignment and a quality score >1 with samtools[4]. This is a very low cut-off and results in only a small loss of reads. To remove any bias introduced by PCR amplification, it is recommended to mark and remove any duplicate reads from the alignments. A strong tool for this is Picard MarkDuplicates[5,6], which can identify optical duplicate reads and marks each duplicate with a 0x400 flag that can be filtered out with samtools.
Note: for our samples only a small number of reads was excluded. However, for less ubiquitous modifications, more amplification may be required to obtain sufficient DNA for sequencing, resulting in more optical duplicate reads.

Next call peaks with macs2[6], using BAMPE (bam paired-end) as input a genome size of 3.8×10^7. For downstream analysis it is recommended to run both individual replicates and all replicated together for peak prediction.

References

1. Johnson, D. S., Mortazavi, A., Myers, R. M. & Wold, B. Genome-wide mapping of in vivo protein-DNA interactions. *Science* **316**, 1497–1502 (2007).
2. Soyer, J. L. *et al.* Chromatin analyses of Zymoseptoria tritici: Methods for chromatin immunoprecipitation followed by high-throughput sequencing (ChIP-seq). *Fungal Genetics and Biology* **79**, 63–70 (2015).
3. Langmead, B. & Salzberg, S. L. Fast gapped-read alignment with Bowtie 2. *Nature Methods* **9**, 357–359 (2012).

4. Li, H. *et al.* The Sequence Alignment/Map format and SAMtools. *Bioinformatics* **25**, 2078–2079 (2009).
5. Broad Institute. Picard Tools - By Broad Institute. *Github* http://broadinstitute.github.io/picard/ (2009).
6. Feng, J., Liu, T., Qin, B., Zhang, Y. & Liu, X. S. Identifying ChIP-seq enrichment using MACS. *Nature Protocols* **7**, 1728–1740 (2012).

Supplemental Figures

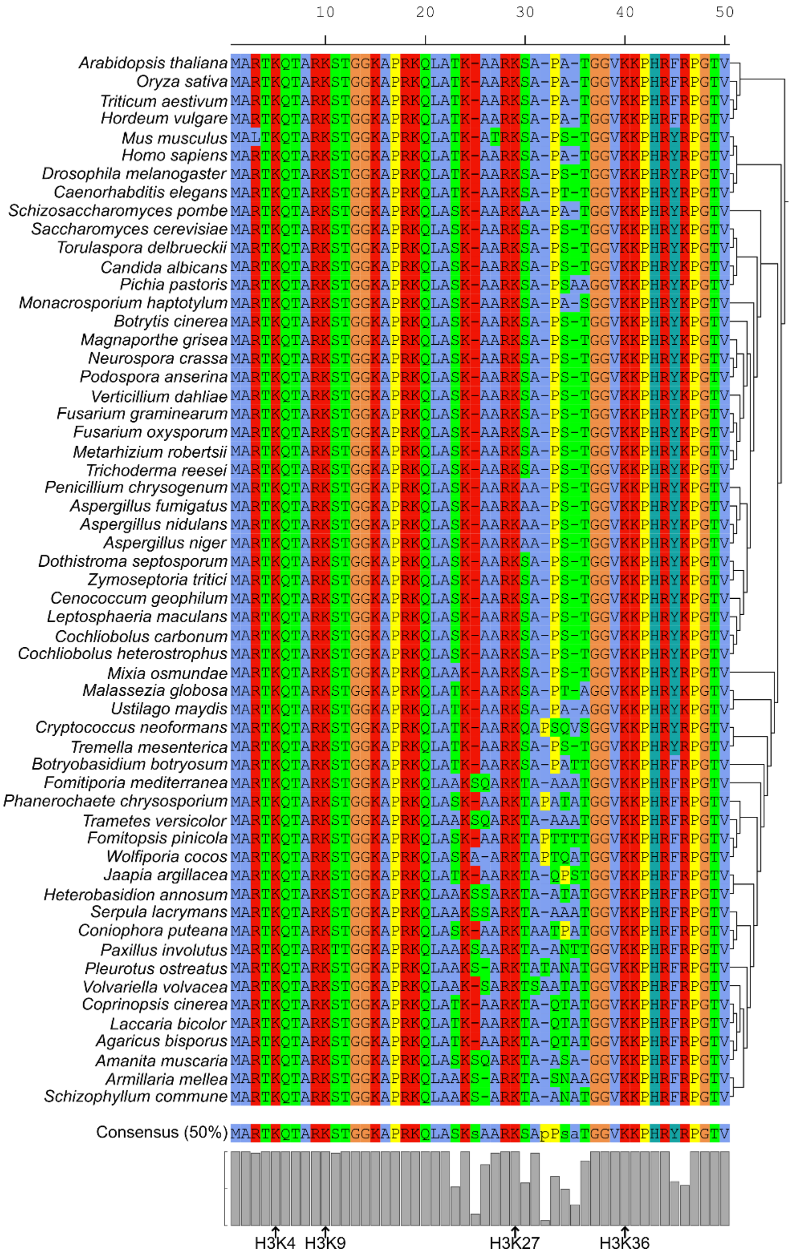

Figure S1: Sequence alignment of histone H3 for 4 animal species, 4 plant species and 49 fungal species. Fungal species included an equal distribution of ascomycete and basidiomycete species across all phylogenetic groups. The area surrounding K4 (the lysin residue at position 4), K9 and K36 is strongly conserved in all species. Around K27 there are multiple substitutions, insertions and deletions that may influence antibody recognition. These changes are primarily found in basidiomycete species.

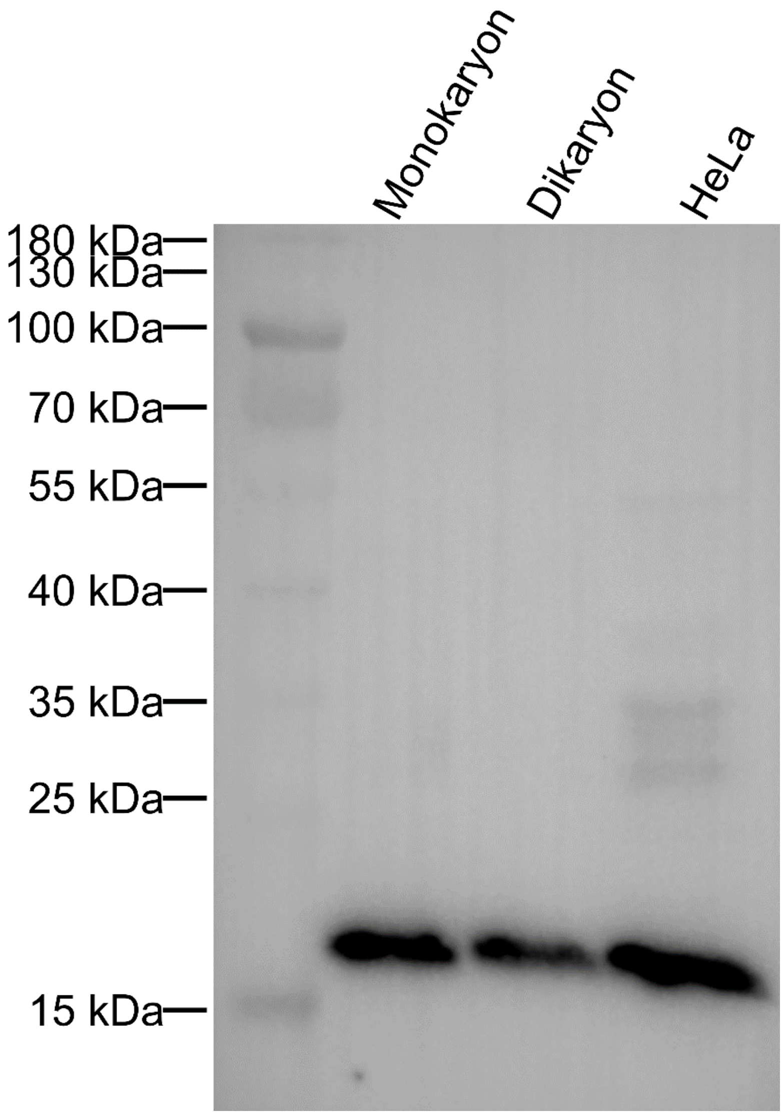

Figure S2: Western blot with anti-H3K4me2 on 88-hour old monokaryon and dikaryon samples and HeLa whole cell lysate detects histone H3 at the expected size of 17 kDa.

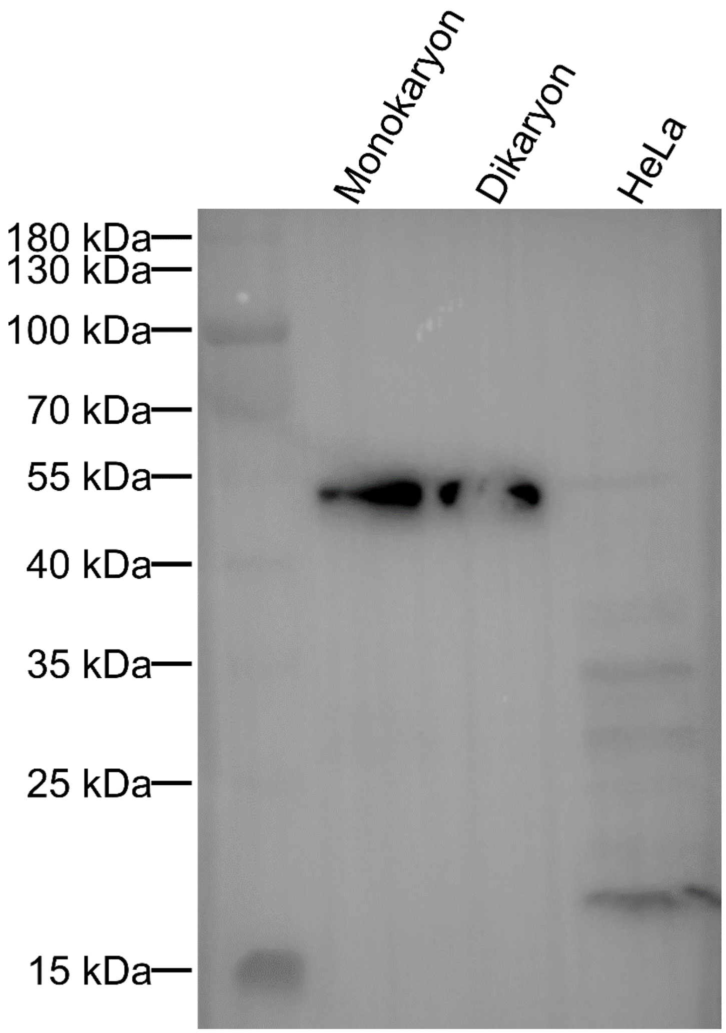

Figure S3: Western blot with anti-H3K27me3 on 88-hour old monokaryon and dikaryon samples does not detect histone H3 at the expected size of 17 kDa. However, there is an aspecific band around 50 kDa of unknown origin. In HeLa whole cell lysate histone H3 is detected at the expected height of 17 kDa.

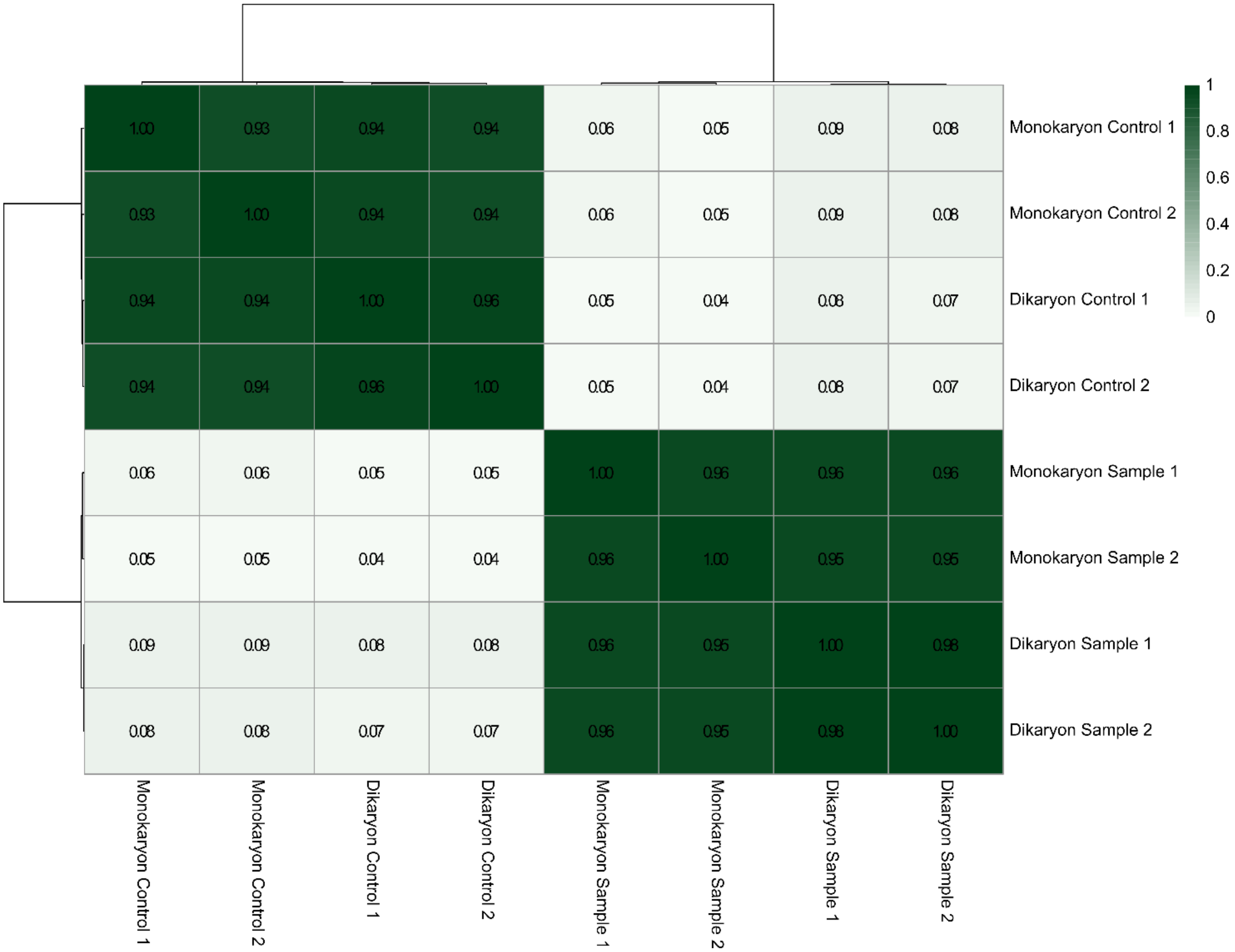

Figure S4: Pearson R correlation between all BigWig files generated from sequence alignments. Within control samples and ChIP samples there is strong correlation between all samples (> 0.93). However, between controls and ChIP samples there is very low correlation (< 0.10). Replicates of the ChIP samples had a higher correlation than between the samples.

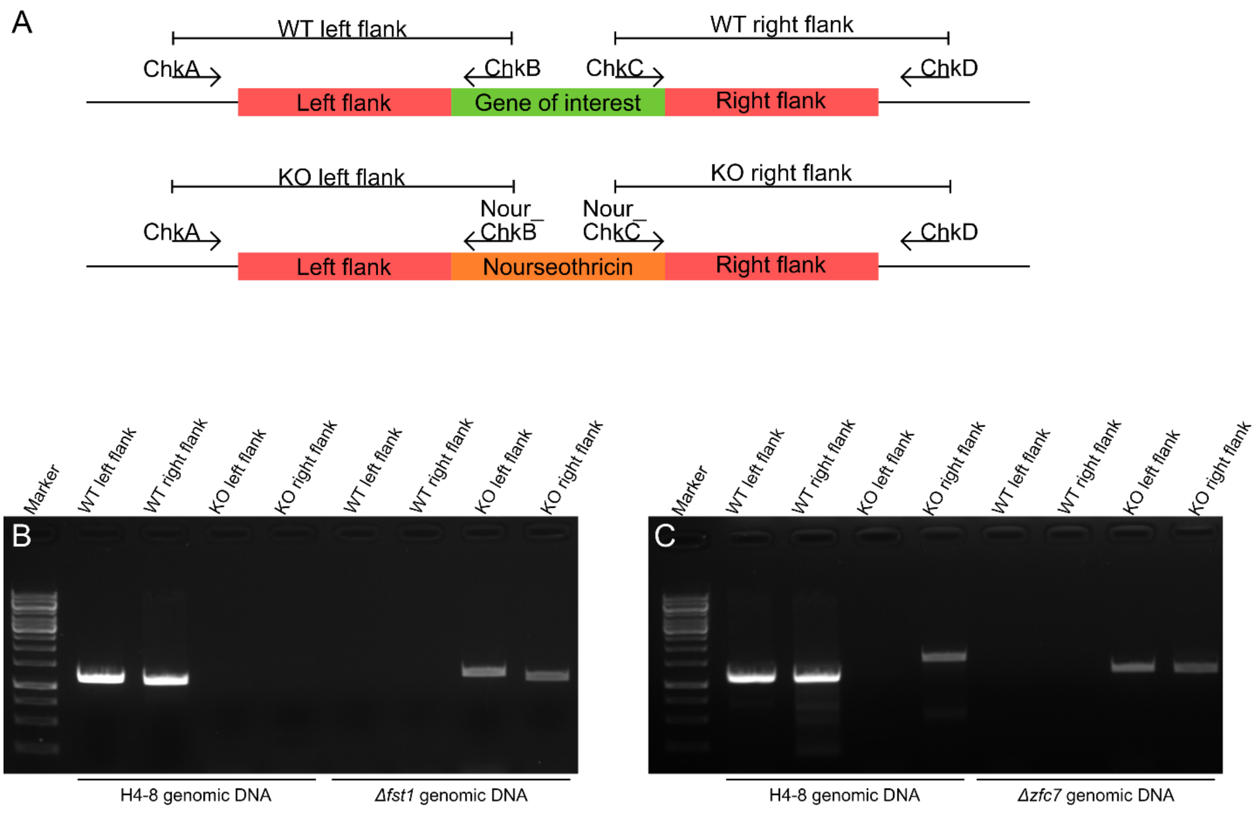

Figure S5: PCR on H4-8 genomic DNA and the gene deletions of *fst1* (B) and *zfc7* (C). Primers used are listed in Table 4. ChkA and D primers bind outside the gene deletion in both the wildtype and deletion strains, while ChkB and C are WT-specific. Nour_ChkB and nour ChkC are deletion-mutant specific (A). PCR should yield a band of ~1 kb in the wildtype using the WT primers, and in the gene deletion using the KO primers. The KO primers of the right flank of *zfc7* detect a non-specific band of 1.5 kb in the wildtype. The marker used is the GeneRuler 1 kb DNA ladder (ThermoFisher Scientific, MA, USA)

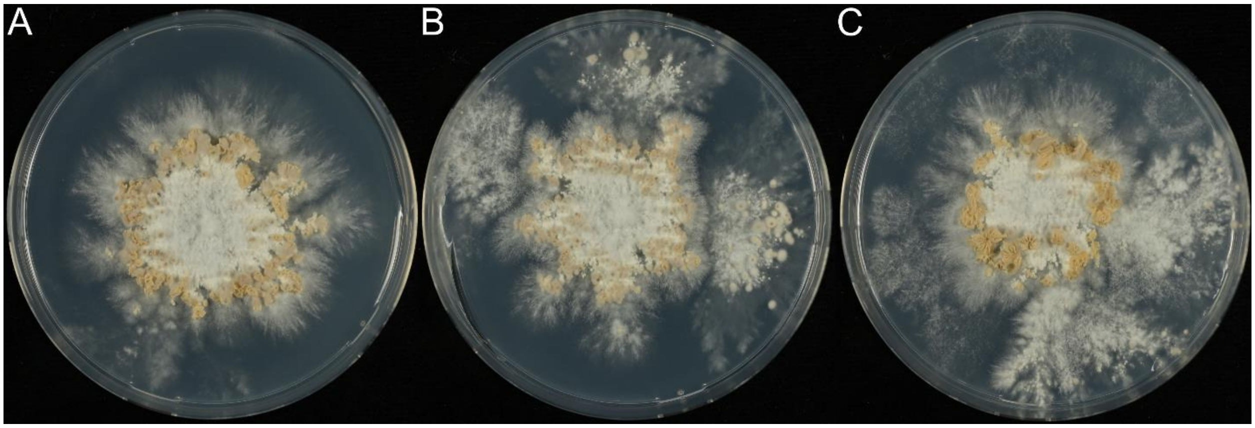

Figure S6: Mushroom development in wild type (A), *Δfst1* crossed with *Δfst1::fst1* (B) and *Δzfc7* crossed with *Δzfc7::zfc7*. In all cases, mature mushrooms are formed, thus restoring the phenotypes of the deletion strains.

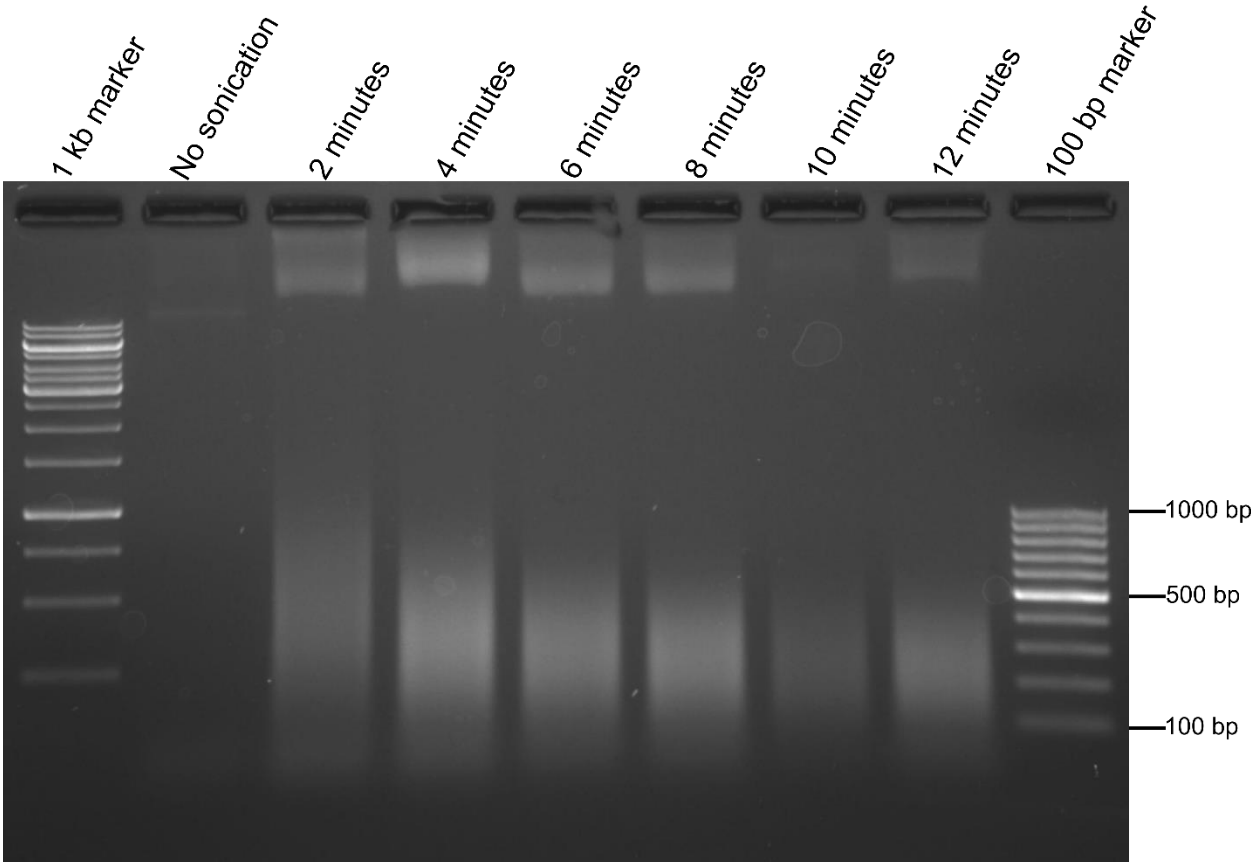

Figure S7: DNA degradation during sonication at different time-points. Before sonication, a genomic DNA band > 10 kb is seen, indicating intact genomic DNA. Sonication resulted in a distribution of fragmented DNA that reached an end-point after 8 minutes, with a fragment size ranging from 60 bp to 700 bp, with a maximum around 250 bp. The large smears found at the top of the lanes after sonication are polysaccharides released from the mycelium during sonication.

Markers used are the GeneRuler 1 kb DNA ladder and GeneRuler 100 bp DNA ladder (ThermoFisher Scientific, MA, USA).

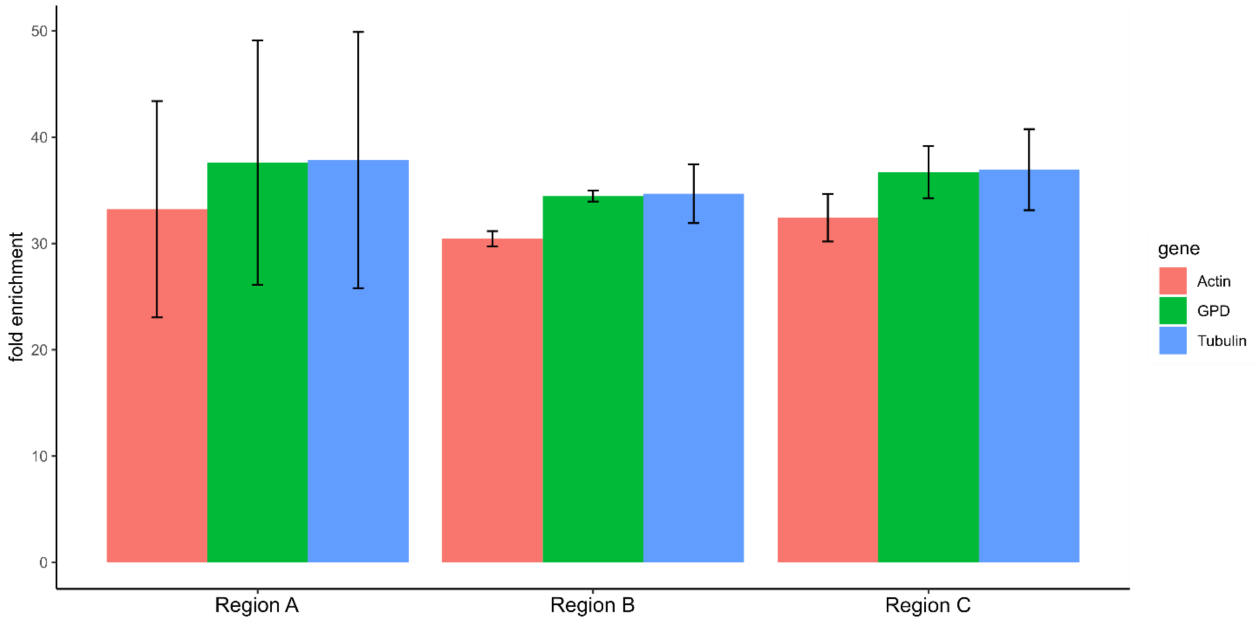

Figure S8: Relative enrichment of actin, GPD and tubulin DNA regions compared to three regions without expression after ChIP. Region A: Scaffold_10: 864,508-864,623. Region B: Scaffold_12: 8,012-8,112. Region C: Scaffold_19: 17,635-17,749.

Table S1. Peaks identified in the monokaryotic and/or dikaryotic samples, as well as any genes that were associated with these peaks.

**scaffold**	**start**	**end**	**concen-tration**	**concen-tration mono-karyon**	**concen-tration dikary-on**	**fold differ-ence**	**p-value**	**Corrected p-value (FDR)**	**Associated gene(s)**	
scaffold_11	262826	263989	8.27	9.21	4.83	4.37	2.69E-55	1.58E-51	Schco3|2066417	
scaffold_3	2706524	2707753	8.51	9.39	5.88	3.51	1.21E-41	3.56E-38	Schco3|2597800	
scaffold_2	3117428	3118862	7.82	8.75	4.46	4.29	3.07E-37	6.01E-34	Schco3|2684833	
scaffold_7	450760	451581	7.63	8.62	1.76	6.86	1.18E-35	1.73E-32		
scaffold_3	2927655	2929651	8.95	9.95	0.43	9.52	2.01E-35	2.37E-32	Schco3|2616525	
scaffold_10	594578	595856	11.03	10.32	11.5	-1.18	9.96E-34	9.75E-31	Schco3|237129	
scaffold_4	2762049	2762780	7.52	8.5	1.91	6.6	7.57E-31	6.36E-28		
scaffold_2	525810	527268	9.46	8.08	10.15	-2.07	1.93E-29	1.42E-26		
scaffold_5	1613303	1615847	10.01	-0.29	11.01	-11.29	1.93E-28	1.26E-25	Schco3|2622454	
scaffold_11	115122	117506	9.8	-0.29	10.8	-11.09	1.87E-27	1.10E-24	Schco3|2065510	
scaffold_2	3473786	3474646	7.82	8.77	3.77	5	5.63E-26	3.01E-23	Schco3|1163017	
scaffold_1	1988783	1992397	9.68	10.23	8.8	1.43	3.21E-25	1.57E-22	Schco3|2022394	
scaffold_2	3221239	3222803	8.43	9.18	6.82	2.36	4.31E-25	1.95E-22	Schco3|2612912	
scaffold_2	1762668	1764596	9.22	-0.29	10.22	-10.51	1.08E-24	4.53E-22	Schco3|2565653	
scaffold_18	183361	185572	9.18	-0.29	10.18	-10.46	1.77E-24	6.94E-22	Schco3|2673869	
scaffold_2	1743563	1745356	9.12	-0.29	10.12	-10.41	3.41E-24	1.25E-21	Schco3|2610872	Schco3|2610874
scaffold_18	188209	189499	8.88	9.88	-0.56	10.44	7.21E-24	2.49E-21	Schco3|2645615	
scaffold_14	244995	246177	8.32	9.11	6.45	2.66	8.70E-24	2.84E-21		
scaffold_14	528168	529430	8.66	6.52	9.48	-2.97	1.18E-23	3.65E-21	Schco3|1248523	Schco3|2519211
scaffold_5	1611617	1612682	8.68	9.68	-0.56	10.24	7.22E-23	2.12E-20		
scaffold_4	2181771	2182889	8.56	9.56	-0.56	10.12	2.19E-22	6.13E-20	Schco3|1349130	
scaffold_1	3120189	3121101	8.74	9.41	7.47	1.94	2.31E-22	6.17E-20	Schco3|2607625	
scaffold_13	894966	895993	8.62	9.39	6.87	2.52	6.70E-22	1.71E-19	Schco3|2642105	
scaffold_11	126630	128224	8.56	-0.29	9.55	-9.84	1.54E-21	3.77E-19		
scaffold_1	615595	616683	8.4	9.39	-0.56	9.95	6.72E-21	1.58E-18	Schco3|2006913	
scaffold_2	2532727	2533649	8.97	9.58	7.89	1.69	7.89E-21	1.78E-18	Schco3|2611904	Schco3|2611907
scaffold_11	224093	226111	10.43	10.84	9.84	1	1.35E-20	2.94E-18	Schco3|2704855	
scaffold_2	1244564	1246693	10.77	11.17	10.23	0.93	1.45E-20	3.05E-18	Schco3|2489147	
scaffold_6	1902720	1903803	8.72	9.35	7.6	1.74	2.53E-20	5.13E-18	Schco3|2626850	

scaffold_4	1889281	1890594	8.19	5.99	9.02	-3.03	1.07E-19	2.10E-17	Schco3|1088480	
scaffold_8	1018046	1018858	7.99	8.99	-0.56	9.55	1.28E-19	2.42E-17	Schco3|1317053	Schco3|2750086
scaffold_7	1322680	1323494	7.88	8.87	-0.56	9.43	4.44E-19	8.15E-17	Schco3|2581163	
scaffold_9	1768485	1769369	7.83	8.83	-0.56	9.39	8.24E-19	1.47E-16	Schco3|1158360	
scaffold_2	1232317	1234586	9.49	9.99	8.71	1.28	1.41E-18	2.43E-16		
scaffold_13	84037	84969	8.66	9.29	7.5	1.79	8.69E-18	1.46E-15	Schco3|2641343	
scaffold_7	1133475	1134239	7.53	8.53	-0.56	9.09	4.32E-17	7.05E-15	Schco3|2505445	
scaffold_12	1184409	1185054	6.56	7.53	1.76	5.77	6.30E-17	1.00E-14	Schco3|2641168	
scaffold_7	2185877	2186955	7.5	5.52	8.3	-2.78	2.63E-16	4.07E-14	Schco3|2630028	
scaffold_14	219984	221503	8.78	9.41	7.66	1.75	3.47E-16	5.22E-14	Schco3|2642607	
scaffold_11	196516	197748	7.4	-0.29	8.39	-8.68	6.30E-16	9.26E-14	Schco3|2066065	
scaffold_7	2319467	2320588	7.19	8.19	-0.56	8.75	1.07E-15	1.53E-13	Schco3|2630148	
scaffold_16	369386	370168	7.18	8.18	-0.56	8.74	1.68E-15	2.33E-13	Schco3|2644610	
scaffold_18	199587	201306	9.36	8.5	9.89	-1.39	1.70E-15	2.33E-13	Schco3|2645630	
scaffold_11	125216	126020	6.54	1.07	7.52	-6.46	2.55E-15	3.40E-13	Schco3|2552351	
scaffold_2	3161735	3164074	8.89	9.42	8.06	1.36	3.13E-15	4.09E-13	Schco3|2488038	Schco3|2612812
scaffold_4	692138	693438	8.81	7.78	9.41	-1.63	3.45E-15	4.41E-13	Schco3|2470416	
scaffold_2	1746613	1747437	7.15	8.15	-0.56	8.71	3.77E-15	4.72E-13	Schco3|2154172	
scaffold_2	206237	207757	8.83	9.35	7.98	1.37	7.15E-15	8.76E-13	Schco3|2609155	
scaffold_9	254481	255718	7.04	8.04	-0.56	8.6	8.23E-15	9.88E-13	Schco3|2690838	
scaffold_1	3604035	3604780	6.98	7.98	-0.56	8.54	1.09E-14	1.28E-12	Schco3|2608151	
scaffold_6	1084008	1084951	6.52	7.5	0.77	6.73	1.16E-14	1.33E-12	Schco3|1171498	Schco3|2289485
scaffold_1	2515616	2516598	6.91	7.9	-0.56	8.46	2.76E-14	3.11E-12	Schco3|2606891	
scaffold_3	1244782	1245923	8.53	9.11	7.53	1.59	3.47E-14	3.85E-12	Schco3|2614543	
scaffold_2	1919524	1920406	7.05	8.05	-0.56	8.61	3.91E-14	4.22E-12	Schco3|2487754	
scaffold_9	279929	280678	7.36	8.18	5.23	2.95	3.95E-14	4.22E-12	Schco3|2703470	
scaffold_2	1455883	1456517	7.06	7.9	4.78	3.12	6.62E-14	6.95E-12	Schco3|2610516	
scaffold_13	1131439	1132181	6.79	7.78	-0.56	8.34	1.40E-13	1.44E-11	Schco3|1263310	Schco3|2642462
scaffold_6	2393017	2393695	6.74	7.74	-0.56	8.3	1.61E-13	1.64E-11	Schco3|2502348	
scaffold_13	980669	985536	10.93	11.24	10.53	0.71	1.76E-13	1.75E-11	Schco3|2642233	
scaffold_1	4327989	4329042	9.74	10.14	9.2	0.93	1.79E-13	1.75E-11	Schco3|2482809	
scaffold_13	147539	148413	6.74	7.74	-0.56	8.3	1.86E-13	1.79E-11	Schco3|2518082	
scaffold_14	319416	320227	6.53	7.5	1.89	5.61	2.76E-13	2.62E-11	Schco3|2556205	
scaffold_1	1500697	1501815	8.56	9.17	7.51	1.66	2.89E-13	2.70E-11	Schco3|2481326	

scaffold_18	248030	248947	6.78	-0.29	7.78	-8.07	4.56E-13	4.19E-11	Schco3|2558619	
scaffold_6	450603	451769	9.07	9.61	8.2	1.41	5.39E-13	4.88E-11	Schco3|2625100	
scaffold_6	2504843	2506314	9.43	10	8.45	1.55	1.12E-12	9.99E-11	Schco3|2627529	
scaffold_4	728391	729424	8.93	9.45	8.12	1.32	2.35E-12	2.06E-10	Schco3|2495301	
scaffold_9	2028028	2029894	9.16	9.65	8.41	1.24	2.44E-12	2.11E-10	Schco3|2635658	
scaffold_7	1059280	1061225	9.12	9.6	8.4	1.19	3.06E-12	2.61E-10		
scaffold_7	1290743	1291510	6.47	7.46	-0.56	8.02	3.85E-12	3.23E-10	Schco3|1190373	
scaffold_1	4296959	4297671	7.9	8.67	6.18	2.49	4.32E-12	3.57E-10	Schco3|2608854	
scaffold_10	1788454	1790986	9.55	9.98	8.94	1.04	4.51E-12	3.68E-10	Schco3|2637835	Schco3|2637838
scaffold_2	1265688	1267100	9.9	10.28	9.4	0.88	5.25E-12	4.23E-10	Schco3|2610293	
scaffold_2	583031	584103	8.58	9.11	7.75	1.36	6.29E-12	5.00E-10	Schco3|1186310	Schco3|2609519
scaffold_1	4246249	4247047	8.65	9.21	7.74	1.47	6.52E-12	5.11E-10	Schco3|2523093	
scaffold_4	2349213	2351977	10.13	10.51	9.63	0.88	7.87E-12	6.08E-10	Schco3|2573048	
scaffold_20	5218	6202	8.95	9.44	8.2	1.25	7.97E-12	6.08E-10	Schco3|2522123	
scaffold_4	1235011	1236325	8.85	8.02	9.38	-1.36	8.66E-12	6.52E-10	Schco3|2535442	
scaffold_8	231528	232270	7.56	8.37	5.48	2.89	8.82E-12	6.56E-10	Schco3|2509510	Schco3|2669177
scaffold_7	318305	319184	7.93	8.64	6.45	2.19	1.14E-11	8.39E-10	Schco3|2627867	
scaffold_11	289432	290935	9.36	9.82	8.68	1.14	1.52E-11	1.10E-09	Schco3|2458967	
scaffold_9	1588374	1588966	6.33	7.32	-0.56	7.88	1.87E-11	1.34E-09	Schco3|2549882	
scaffold_8	432951	433759	6.48	-0.29	7.47	-7.76	2.70E-11	1.91E-09		
scaffold_15	582131	582950	6.39	-0.29	7.39	-7.68	3.13E-11	2.19E-09	Schco3|2644174	Schco3|2644178
scaffold_10	701687	702352	6.8	7.64	4.53	3.11	3.62E-11	2.50E-09	Schco3|2636602	
scaffold_10	621002	621652	6.36	-0.29	7.35	-7.64	4.42E-11	3.02E-09	Schco3|2704336	
scaffold_6	1875027	1876060	6.41	-0.29	7.4	-7.69	5.36E-11	3.62E-09	Schco3|2626803	
scaffold_5	3072728	3073764	6.24	7.23	-0.56	7.79	5.90E-11	3.94E-09	Schco3|2624387	Schco3|2624391
scaffold_2	3064582	3065364	6.26	7.25	-0.56	7.81	6.77E-11	4.47E-09	Schco3|2530642	Schco3|2612640
scaffold_11	350105	350791	6.33	7.33	-0.56	7.89	7.24E-11	4.73E-09	Schco3|2588858	Schco3|2704900
scaffold_17	192246	193240	8.97	9.44	8.26	1.18	8.01E-11	5.17E-09	Schco3|2603637	
scaffold_14	535444	536257	6.3	-0.29	7.29	-7.58	1.44E-10	9.23E-09	Schco3|2592626	
scaffold_4	1354059	1355576	9.67	10.05	9.17	0.88	1.50E-10	9.45E-09	Schco3|2495727	
scaffold_2	1183480	1184480	8.22	8.83	7.13	1.69	1.72E-10	1.07E-08	Schco3|2565008	
scaffold_8	2177396	2178321	6.24	-0.29	7.23	-7.52	1.94E-10	1.20E-08	Schco3|1214686	
scaffold_9	275841	276620	8.13	8.78	6.95	1.83	2.55E-10	1.56E-08	Schco3|2357467	
scaffold_5	2965593	2966460	6.3	-0.29	7.29	-7.58	2.67E-10	1.62E-08	Schco3|2624256	

<table>
<tr><td>scaffold_8</td><td>2114271</td><td>2115135</td><td>8.61</td><td>9.12</td><td>7.8</td><td>1.32</td><td>2.99E-10</td><td>1.79E-08</td><td>Schco3|2632813</td><td></td></tr>
<tr><td>scaffold_2</td><td>1242389</td><td>1242955</td><td>6.24</td><td>-0.29</td><td>7.23</td><td>-7.52</td><td>4.38E-10</td><td>2.60E-08</td><td>Schco3|2744136</td><td></td></tr>
<tr><td>scaffold_6</td><td>1684314</td><td>1685265</td><td>9.32</td><td>9.72</td><td>8.76</td><td>0.96</td><td>4.64E-10</td><td>2.71E-08</td><td>Schco3|2626551</td><td></td></tr>
<tr><td>scaffold_10</td><td>1576771</td><td>1577544</td><td>6.05</td><td>7.04</td><td>-0.56</td><td>7.6</td><td>4.65E-10</td><td>2.71E-08</td><td>Schco3|2602114</td><td></td></tr>
<tr><td>scaffold_10</td><td>1164789</td><td>1165334</td><td>6.04</td><td>7.03</td><td>-0.56</td><td>7.59</td><td>5.60E-10</td><td>3.23E-08</td><td>Schco3|1332340</td><td>Schco3|2637052</td></tr>
<tr><td>scaffold_5</td><td>2333015</td><td>2333535</td><td>6.01</td><td>0.4</td><td>7</td><td>-6.6</td><td>5.70E-10</td><td>3.25E-08</td><td>Schco3|2623344</td><td></td></tr>
<tr><td>scaffold_2</td><td>1751434</td><td>1753877</td><td>10.6</td><td>10.88</td><td>10.25</td><td>0.63</td><td>5.76E-10</td><td>3.26E-08</td><td>Schco3|2675477</td><td></td></tr>
<tr><td>scaffold_10</td><td>751034</td><td>752013</td><td>7.03</td><td>7.92</td><td>4.26</td><td>3.65</td><td>6.31E-10</td><td>3.53E-08</td><td>Schco3|2512531</td><td></td></tr>
<tr><td>scaffold_4</td><td>1317953</td><td>1318623</td><td>7.55</td><td>8.25</td><td>6.14</td><td>2.11</td><td>8.70E-10</td><td>4.82E-08</td><td>Schco3|2535527</td><td></td></tr>
<tr><td>scaffold_2</td><td>3229801</td><td>3230748</td><td>8.13</td><td>8.7</td><td>7.18</td><td>1.52</td><td>9.18E-10</td><td>5.04E-08</td><td>Schco3|2612921</td><td></td></tr>
<tr><td>scaffold_2</td><td>299186</td><td>300189</td><td>7.58</td><td>8.25</td><td>6.29</td><td>1.96</td><td>9.94E-10</td><td>5.41E-08</td><td>Schco3|2662412</td><td></td></tr>
<tr><td>scaffold_2</td><td>287007</td><td>288075</td><td>9.56</td><td>9.94</td><td>9.03</td><td>0.91</td><td>1.26E-09</td><td>6.79E-08</td><td>Schco3|2487489</td><td>Schco3|2609245</td></tr>
<tr><td>scaffold_14</td><td>114994</td><td>115976</td><td>9.14</td><td>9.6</td><td>8.47</td><td>1.13</td><td>1.69E-09</td><td>9.04E-08</td><td>Schco3|2519496</td><td></td></tr>
<tr><td>scaffold_4</td><td>1511265</td><td>1511852</td><td>5.94</td><td>6.93</td><td>-0.56</td><td>7.49</td><td>1.72E-09</td><td>9.08E-08</td><td>Schco3|2618632</td><td></td></tr>
<tr><td>scaffold_8</td><td>1381975</td><td>1382526</td><td>6.09</td><td>7.06</td><td>1.62</td><td>5.44</td><td>1.73E-09</td><td>9.10E-08</td><td>Schco3|2631920</td><td></td></tr>
<tr><td>scaffold_3</td><td>2583622</td><td>2584538</td><td>8.4</td><td>8.93</td><td>7.54</td><td>1.39</td><td>1.92E-09</td><td>1.00E-07</td><td></td><td></td></tr>
<tr><td>scaffold_7</td><td>862814</td><td>863967</td><td>8.4</td><td>9</td><td>7.36</td><td>1.63</td><td>2.57E-09</td><td>1.32E-07</td><td></td><td></td></tr>
<tr><td>scaffold_4</td><td>1251315</td><td>1252292</td><td>8.37</td><td>7.42</td><td>8.94</td><td>-1.52</td><td>3.13E-09</td><td>1.60E-07</td><td>Schco3|2618320</td><td></td></tr>
<tr><td>scaffold_3</td><td>2663960</td><td>2665374</td><td>9.25</td><td>9.69</td><td>8.61</td><td>1.08</td><td>3.44E-09</td><td>1.74E-07</td><td>Schco3|2616244</td><td></td></tr>
<tr><td>scaffold_7</td><td>254420</td><td>255549</td><td>8.55</td><td>9.05</td><td>7.76</td><td>1.29</td><td>3.54E-09</td><td>1.78E-07</td><td>Schco3|2627802</td><td></td></tr>
<tr><td>scaffold_1</td><td>4343161</td><td>4343959</td><td>7.35</td><td>8.17</td><td>5.23</td><td>2.95</td><td>5.33E-09</td><td>2.65E-07</td><td>Schco3|2608895</td><td></td></tr>
<tr><td>scaffold_1</td><td>4182061</td><td>4182917</td><td>6.31</td><td>2.27</td><td>7.27</td><td>-5</td><td>5.88E-09</td><td>2.90E-07</td><td>Schco3|2608744</td><td></td></tr>
<tr><td>scaffold_10</td><td>1021276</td><td>1022525</td><td>9.22</td><td>9.62</td><td>8.66</td><td>0.96</td><td>6.29E-09</td><td>3.08E-07</td><td>Schco3|2751591</td><td></td></tr>
<tr><td>scaffold_1</td><td>1888336</td><td>1888796</td><td>5.89</td><td>-0.29</td><td>6.88</td><td>-7.17</td><td>6.68E-09</td><td>3.25E-07</td><td>Schco3|1084888</td><td>Schco3|2606175</td></tr>
<tr><td>scaffold_9</td><td>341078</td><td>342573</td><td>7.53</td><td>8.33</td><td>5.52</td><td>2.81</td><td>7.75E-09</td><td>3.73E-07</td><td>Schco3|2633389</td><td></td></tr>
<tr><td>scaffold_3</td><td>1276821</td><td>1278302</td><td>10.06</td><td>10.38</td><td>9.65</td><td>0.72</td><td>7.92E-09</td><td>3.77E-07</td><td>Schco3|2532119</td><td>Schco3|2614573</td></tr>
<tr><td>scaffold_1</td><td>3391208</td><td>3391889</td><td>5.89</td><td>-0.29</td><td>6.88</td><td>-7.16</td><td>7.95E-09</td><td>3.77E-07</td><td>Schco3|2607913</td><td></td></tr>
<tr><td>scaffold_4</td><td>3214856</td><td>3218352</td><td>9.91</td><td>9.46</td><td>10.25</td><td>-0.79</td><td>8.46E-09</td><td>3.98E-07</td><td>Schco3|2598675</td><td>Schco3|2747038</td></tr>
<tr><td>scaffold_12</td><td>99098</td><td>99701</td><td>5.76</td><td>6.75</td><td>-0.56</td><td>7.31</td><td>9.86E-09</td><td>4.60E-07</td><td>Schco3|2080555</td><td>Schco3|2516273</td></tr>
<tr><td>scaffold_7</td><td>550434</td><td>553998</td><td>10.38</td><td>10.7</td><td>9.96</td><td>0.74</td><td>1.04E-08</td><td>4.79E-07</td><td>Schco3|2668517</td><td></td></tr>
<tr><td>scaffold_2</td><td>573991</td><td>574637</td><td>5.78</td><td>6.77</td><td>-0.56</td><td>7.33</td><td>1.18E-08</td><td>5.37E-07</td><td>Schco3|2609508</td><td></td></tr>
<tr><td>scaffold_5</td><td>1465732</td><td>1466890</td><td>8.28</td><td>8.83</td><td>7.39</td><td>1.44</td><td>1.18E-08</td><td>5.37E-07</td><td>Schco3|2538948</td><td></td></tr>
<tr><td>scaffold_17</td><td>405504</td><td>406148</td><td>5.84</td><td>-0.29</td><td>6.83</td><td>-7.12</td><td>1.19E-08</td><td>5.38E-07</td><td></td><td></td></tr>
<tr><td>scaffold_10</td><td>1746224</td><td>1747218</td><td>7.84</td><td>8.45</td><td>6.75</td><td>1.7</td><td>1.31E-08</td><td>5.88E-07</td><td>Schco3|1159800</td><td></td></tr>
</table>

scaffold_7	1742588	1743904	9.59	10.03	8.97	1.05	1.40E-08	6.25E-07	Schco3|2629581	
scaffold_9	773968	774784	7.99	8.58	6.96	1.62	1.93E-08	8.54E-07	Schco3|1157466	
scaffold_2	3346473	3348271	10.2	10.51	9.82	0.68	2.04E-08	8.95E-07	Schco3|1135989	Schco3|2567539
scaffold_8	929345	930146	8.57	9.03	7.89	1.14	2.27E-08	9.87E-07	Schco3|2631311	
scaffold_1	3470185	3471236	9.22	9.61	8.68	0.93	2.58E-08	1.12E-06	Schco3|2481375	Schco3|2481850
scaffold_5	1238241	1239672	9.2	9.61	8.61	1	2.87E-08	1.23E-06	Schco3|1113856	
scaffold_14	943380	943882	5.82	-0.29	6.81	-7.09	3.18E-08	1.35E-06	Schco3|2673190	
scaffold_7	1363182	1363830	5.81	-0.29	6.8	-7.08	3.32E-08	1.40E-06	Schco3|2629138	
scaffold_8	2406073	2406518	5.63	6.62	-0.56	7.18	3.71E-08	1.56E-06	Schco3|2633058	
scaffold_5	2056774	2057939	8.91	9.39	8.18	1.2	3.80E-08	1.58E-06	Schco3|2262994	
scaffold_2	3499677	3501246	8.95	9.34	8.42	0.93	4.32E-08	1.79E-06	Schco3|2567695	Schco3|2567698
scaffold_15	276082	277310	9.96	10.35	9.42	0.92	5.43E-08	2.23E-06	Schco3|2643858	
scaffold_3	548178	549264	8.45	8.98	7.61	1.37	5.69E-08	2.32E-06		
scaffold_7	1754483	1756779	9.91	9.41	10.27	-0.86	5.99E-08	2.43E-06	Schco3|2629605	
scaffold_11	233559	233974	5.67	-0.29	6.66	-6.95	6.86E-08	2.76E-06	Schco3|60621	
scaffold_1	4262140	4263808	9.12	9.52	8.57	0.95	7.39E-08	2.95E-06	Schco3|2482925	Schco3|2743676
scaffold_4	2121150	2121766	6.77	7.54	5.02	2.52	7.46E-08	2.96E-06	Schco3|2619160	
scaffold_1	3457144	3457544	5.58	6.55	0.77	5.78	7.56E-08	2.98E-06	Schco3|2608014	
scaffold_9	343761	346284	10.09	10.4	9.7	0.7	8.36E-08	3.28E-06	Schco3|2509997	
scaffold_5	1746779	1747620	8.36	8.84	7.63	1.21	8.53E-08	3.32E-06	Schco3|2747679	
scaffold_7	1007297	1008174	5.55	6.54	-0.56	7.1	8.80E-08	3.40E-06		
scaffold_10	747536	748699	9.22	8.56	9.67	-1.1	1.03E-07	3.94E-06	Schco3|2636674	
scaffold_9	15441	15893	6.15	7.1	2.36	4.74	1.13E-07	4.30E-06		
scaffold_5	1859262	1860031	7.38	8.03	6.16	1.87	1.13E-07	4.30E-06	Schco3|2539413	
scaffold_1	1525144	1526300	8.84	9.28	8.21	1.07	1.15E-07	4.32E-06	Schco3|2524481	
scaffold_11	377625	379116	9.83	9.46	10.13	-0.67	1.37E-07	5.14E-06	Schco3|2638422	
scaffold_5	2108135	2109333	8.73	9.17	8.09	1.08	1.38E-07	5.14E-06	Schco3|2263509	
scaffold_3	3236562	3237701	8.02	8.55	7.18	1.37	1.48E-07	5.47E-06	Schco3|2616871	
scaffold_11	220889	222257	9.18	9.55	8.68	0.87	1.71E-07	6.27E-06	Schco3|83222	
scaffold_11	231710	233339	9.41	9.75	8.97	0.79	1.89E-07	6.89E-06	Schco3|17250	
scaffold_3	1616867	1618082	8.42	8.89	7.72	1.17	1.91E-07	6.94E-06	Schco3|2614981	
scaffold_10	1666824	1668615	9.41	9.76	8.93	0.83	1.95E-07	7.04E-06	Schco3|2062189	Schco3|2637680
scaffold_7	1913002	1913900	7.77	8.39	6.65	1.74	2.11E-07	7.56E-06	Schco3|2629741	
scaffold_2	885534	885977	5.46	6.45	-0.56	7.01	2.22E-07	7.90E-06	Schco3|2528059	

scaffold_13	342557	343565	8.26	7.53	8.74	-1.21	2.46E-07	8.69E-06	Schco3|2641534	
scaffold_1	518623	519257	7.2	7.86	5.92	1.94	2.50E-07	8.79E-06	Schco3|2742326	
scaffold_8	1210365	1211776	9.17	9.5	8.72	0.78	2.74E-07	9.58E-06	Schco3|2631700	
scaffold_2	982765	983575	8.75	9.19	8.13	1.06	2.83E-07	9.86E-06	Schco3|2528175	Schco3|2609955
scaffold_10	389215	391073	10.25	10.6	9.78	0.82	2.85E-07	9.86E-06	Schco3|2670907	
scaffold_10	347735	349038	9.19	9.54	8.73	0.81	2.96E-07	1.02E-05	Schco3|2636140	
scaffold_11	760693	761365	7.46	8.2	5.84	2.36	3.00E-07	1.02E-05	Schco3|1216147	Schco3|2638739
scaffold_2	3526137	3526819	5.53	-0.29	6.52	-6.81	3.03E-07	1.03E-05	Schco3|2613255	
scaffold_11	325515	326496	7.6	6.56	8.2	-1.64	3.53E-07	1.19E-05	Schco3|2638350	
scaffold_2	782444	783582	8.25	8.73	7.53	1.2	3.67E-07	1.23E-05	Schco3|2145092	
scaffold_8	546924	548199	9.84	10.22	9.32	0.9	3.68E-07	1.23E-05	Schco3|2630811	
scaffold_7	2141306	2142508	9.12	9.47	8.64	0.83	3.73E-07	1.24E-05	Schco3|2629974	
scaffold_1	2825713	2827222	8.04	8.57	7.19	1.39	3.99E-07	1.32E-05		
scaffold_14	354999	358649	11.1	11.38	10.76	0.63	4.81E-07	1.58E-05	Schco3|2519603	
scaffold_2	2406786	2408275	9.02	9.42	8.46	0.97	4.98E-07	1.63E-05	Schco3|2489526	Schco3|2611745
scaffold_4	612169	613598	9.14	9.57	8.52	1.04	5.29E-07	1.72E-05	Schco3|2665179	
scaffold_2	3374948	3375925	8.08	8.6	7.26	1.34	5.37E-07	1.73E-05	Schco3|2613084	
scaffold_8	1796356	1797629	9.76	10.04	9.41	0.63	5.53E-07	1.78E-05	Schco3|2547616	
scaffold_10	1873130	1873902	7.95	8.47	7.14	1.33	5.68E-07	1.81E-05		
scaffold_16	295691	296590	9.24	9.64	8.68	0.97	5.69E-07	1.81E-05	Schco3|1259931	
scaffold_4	1058396	1058862	6.14	7.08	2.52	4.57	5.75E-07	1.82E-05	Schco3|2618084	Schco3|2618085
scaffold_5	1280442	1281413	8.14	7.35	8.64	-1.29	5.81E-07	1.83E-05	Schco3|1113897	
scaffold_5	766278	767479	9.55	9.86	9.15	0.71	6.06E-07	1.89E-05	Schco3|2621408	
scaffold_4	1022640	1023622	9.26	9.58	8.85	0.73	6.76E-07	2.10E-05	Schco3|2618038	
scaffold_10	502865	503772	8.49	8.94	7.85	1.09	7.69E-07	2.38E-05	Schco3|2550939	
scaffold_1	1008030	1008682	7.41	8.03	6.31	1.72	8.50E-07	2.61E-05	Schco3|1146732	
scaffold_8	284231	284918	7.15	7.84	5.79	2.05	8.53E-07	2.61E-05	Schco3|2679176	
scaffold_16	228361	229366	8.68	9.2	7.85	1.35	8.82E-07	2.69E-05	Schco3|2644458	
scaffold_4	2626468	2628170	9.6	9.91	9.21	0.71	8.96E-07	2.70E-05		
scaffold_8	859682	860205	5.4	-0.29	6.39	-6.68	8.96E-07	2.70E-05		
scaffold_2	2268118	2268792	6.11	7.05	2.52	4.53	9.42E-07	2.83E-05	Schco3|2529670	
scaffold_4	2615403	2616274	8.47	8.96	7.73	1.23	1.01E-06	3.01E-05	Schco3|234397	
scaffold_3	1541997	1543635	8.73	9.16	8.12	1.04	1.02E-06	3.04E-05	Schco3|2568997	Schco3|2685428
scaffold_2	1960078	1960937	8.44	8.88	7.79	1.09	1.09E-06	3.21E-05	Schco3|2529309	Schco3|2611154

scaffold_7	1320466	1321427	8.81	9.18	8.32	0.86	1.12E-06	3.28E-05	Schco3|1154396	
scaffold_3	344722	346498	10	10.29	9.63	0.66	1.22E-06	3.56E-05	Schco3|2181177	
scaffold_10	628763	629189	6.01	6.91	3.09	3.82	1.25E-06	3.64E-05	Schco3|2712119	
scaffold_8	1238155	1239102	8.66	9.04	8.13	0.91	1.28E-06	3.70E-05	Schco3|1155975	
scaffold_2	1148126	1149098	7.41	8.06	6.21	1.84	1.38E-06	3.98E-05	Schco3|2148653	Schco3|2610156
scaffold_1	108515	109449	8.77	9.16	8.25	0.91	1.40E-06	4.02E-05	Schco3|2000796	Schco3|2660710
scaffold_15	512012	513037	9.32	9.67	8.86	0.81	1.63E-06	4.66E-05	Schco3|1136736	
scaffold_13	375712	376179	5.21	6.19	0.02	6.17	1.86E-06	5.27E-05		
scaffold_5	2011328	2012293	9	9.35	8.55	0.8	1.94E-06	5.48E-05	Schco3|2498569	
scaffold_8	1101180	1102902	9.44	9.75	9.05	0.7	2.04E-06	5.73E-05	Schco3|2631554	
scaffold_4	1877780	1878279	5.22	6.2	-0.56	6.76	2.06E-06	5.76E-05	Schco3|2618915	
scaffold_8	833066	833733	6.01	6.91	3.08	3.82	2.17E-06	6.05E-05	Schco3|1190859	Schco3|2631173
scaffold_7	817572	818601	7.9	6.97	8.46	-1.49	2.24E-06	6.19E-05	Schco3|2668609	
scaffold_5	2077967	2078925	7.94	8.51	7.01	1.49	2.24E-06	6.19E-05	Schco3|2623001	
scaffold_2	213434	213935	5.2	6.18	-0.56	6.74	2.33E-06	6.39E-05	Schco3|2662380	
scaffold_14	560722	563169	10.67	10.89	10.41	0.48	2.43E-06	6.64E-05	Schco3|2642982	
scaffold_6	2467206	2467727	6.11	6.97	3.72	3.24	2.54E-06	6.91E-05	Schco3|2501785	
scaffold_14	457210	458113	9.61	9.92	9.21	0.7	2.71E-06	7.35E-05	Schco3|2642909	
scaffold_18	353005	353502	5.28	-0.29	6.27	-6.56	3.01E-06	8.10E-05	Schco3|2594925	
scaffold_10	1515032	1515489	5.16	6.15	-0.56	6.71	3.14E-06	8.42E-05	Schco3|2060873	
scaffold_2	1952080	1953042	9.32	9.66	8.88	0.78	3.45E-06	9.22E-05	Schco3|2488527	Schco3|2721494
scaffold_6	1302500	1303943	8.59	8.98	8.06	0.92	3.58E-06	9.51E-05	Schco3|2626124	
scaffold_1	994710	995145	5.15	6.13	-0.56	6.69	3.61E-06	9.54E-05	Schco3|2482226	Schco3|2605066
scaffold_11	245096	250565	11.09	11.29	10.86	0.43	3.62E-06	9.54E-05	Schco3|1034973	
scaffold_1	1204182	1204630	5.15	6.14	-0.56	6.7	3.66E-06	9.60E-05		
scaffold_5	2022751	2024032	9.26	9.56	8.87	0.69	3.69E-06	9.65E-05	Schco3|2730626	
scaffold_5	248118	249065	8.02	8.59	7.07	1.52	3.87E-06	0.000101	Schco3|2699781	
scaffold_8	1556771	1558329	9.58	9.24	9.85	-0.62	3.89E-06	0.000101	Schco3|2632118	
scaffold_6	444542	445073	5.19	6.18	-0.56	6.74	4.01E-06	0.000103	Schco3|2625086	
scaffold_8	1416149	1416624	5.13	6.12	-0.56	6.68	4.02E-06	0.000103	Schco3|2345780	Schco3|2703034
scaffold_12	736574	737649	8.95	9.3	8.48	0.81	4.12E-06	0.000105	Schco3|2088105	
scaffold_11	989327	990765	8.91	8.46	9.25	-0.79	4.21E-06	0.000107	Schco3|258402	
scaffold_9	868310	870900	10.54	10.87	10.12	0.74	4.42E-06	0.000112	Schco3|2364181	
scaffold_7	1767428	1769688	10.09	10.33	9.8	0.53	4.57E-06	0.000115	Schco3|2629622	

scaffold_4	3132628	3133471	6.49	3.42	7.4	-3.98	4.75E-06	0.000119	Schco3|2620286	
scaffold_2	3702317	3703170	9.46	9.79	9.03	0.76	4.86E-06	0.000122	Schco3|2597182	Schco3|2745057
scaffold_9	1044079	1044580	5.8	6.69	2.96	3.73	5.05E-06	0.000126		
scaffold_17	410859	411313	5.2	-0.29	6.18	-6.47	5.24E-06	0.00013	Schco3|2645260	
scaffold_3	2912923	2913849	6.14	6.99	3.79	3.2	5.26E-06	0.00013	Schco3|2616512	
scaffold_1	2523044	2528302	11.61	11.42	11.77	-0.35	5.48E-06	0.000135	Schco3|1118382	
scaffold_10	574277	575579	9.19	9.56	8.7	0.87	5.91E-06	0.000145	Schco3|2636417	
scaffold_1	2223381	2224646	9.7	9.99	9.35	0.64	5.99E-06	0.000146	Schco3|2742971	
scaffold_9	1365079	1366235	8.73	8.1	9.17	-1.08	6.12E-06	0.000149	Schco3|1100542	Schco3|2369271
scaffold_10	537150	538517	7	5.22	7.78	-2.56	6.17E-06	0.000149	Schco3|1330447	
scaffold_3	1081111	1082282	9.17	9.49	8.75	0.73	6.30E-06	0.000152	Schco3|2676119	
scaffold_4	2019124	2020068	9.18	9.5	8.76	0.74	6.52E-06	0.000156	Schco3|2619044	
scaffold_14	974971	975541	7.15	7.91	5.4	2.51	6.53E-06	0.000156	Schco3|2643439	
scaffold_10	1442193	1442915	7.75	8.3	6.85	1.46	6.68E-06	0.000159	Schco3|2637388	Schco3|2637391
scaffold_8	1049072	1049524	5.38	6.34	1.02	5.33	7.04E-06	0.000167	Schco3|2631487	
scaffold_11	701343	702187	9.07	9.4	8.63	0.78	7.21E-06	0.00017	Schco3|2638685	
scaffold_2	1597596	1599410	9.5	9.79	9.15	0.64	7.45E-06	0.000175	Schco3|2610670	Schco3|2610672
scaffold_10	1226536	1227116	6.51	7.25	4.86	2.39	7.65E-06	0.000179	Schco3|2637158	
scaffold_18	177791	179222	9.55	9.21	9.82	-0.61	8.19E-06	0.000191	Schco3|2645610	
scaffold_5	289421	290617	8.71	9.1	8.18	0.93	8.28E-06	0.000192	Schco3|2500455	
scaffold_5	2504718	2506209	9.06	9.44	8.54	0.9	8.68E-06	0.000201	Schco3|2623594	Schco3|2700610
scaffold_4	3458767	3459657	8.74	9.11	8.24	0.87	8.83E-06	0.000203		
scaffold_9	1797756	1798490	7.19	7.86	5.9	1.96	8.94E-06	0.000205	Schco3|2635346	
scaffold_5	417411	418900	8.68	9.07	8.15	0.91	9.66E-06	0.000221	Schco3|2574283	
scaffold_7	2369834	2372043	9.03	9.44	8.44	0.99	1.00E-05	0.000228	Schco3|2630203	
scaffold_1	2850916	2851896	8.47	8.85	7.95	0.91	1.04E-05	0.000235		
scaffold_5	3223956	3224501	5.55	1.73	6.5	-4.77	1.04E-05	0.000235	Schco3|2624579	
scaffold_5	2887897	2888935	9.65	9.93	9.3	0.63	1.05E-05	0.000236	Schco3|2624151	
scaffold_1	3766352	3767650	8.85	9.25	8.31	0.94	1.17E-05	0.000263	Schco3|2608324	
scaffold_3	1089372	1089744	5.01	6	-0.56	6.56	1.19E-05	0.000266	Schco3|2187853	
scaffold_4	1183569	1184425	7.74	8.28	6.87	1.4	1.20E-05	0.000267	Schco3|2618228	
scaffold_2	115183	117440	10.61	10.85	10.33	0.52	1.21E-05	0.000269	Schco3|2662337	
scaffold_5	1106839	1107923	9.16	9.46	8.78	0.67	1.27E-05	0.00028	Schco3|2253768	Schco3|2500762
scaffold_18	82651	83735	8.54	7.97	8.95	-0.98	1.27E-05	0.00028	Schco3|2645537	

scaffold_3	1942635	1943666	9.36	9.66	8.99	0.66	1.41E-05	0.000308	Schco3|2615368	
scaffold_1	2820451	2821208	7.85	8.41	6.94	1.47	1.41E-05	0.000308		
scaffold_1	2569505	2570600	9.24	9.53	8.88	0.65	1.42E-05	0.000308	Schco3|2661640	
scaffold_5	2181735	2182763	8.66	9.03	8.17	0.85	1.42E-05	0.000308	Schco3|2623156	
scaffold_4	2564238	2565778	9.94	10.19	9.64	0.55	1.46E-05	0.000316	Schco3|2619710	
scaffold_10	515615	515967	5.51	6.46	1.77	4.69	1.50E-05	0.000323		
scaffold_1	2852912	2853878	7.64	6.74	8.19	-1.45	1.56E-05	0.000333	Schco3|1186902	
scaffold_5	2397660	2398311	6.02	2.85	6.93	-4.09	1.56E-05	0.000333	Schco3|2730943	
scaffold_7	841852	842638	7.89	8.43	7.02	1.41	1.59E-05	0.000338	Schco3|2628501	
scaffold_11	137373	138324	7.79	7.04	8.29	-1.24	1.68E-05	0.000357	Schco3|1174780	
scaffold_11	653812	655075	9.62	9.9	9.28	0.62	1.79E-05	0.000378	Schco3|2638615	
scaffold_9	1790350	1791793	7.99	8.58	6.98	1.6	1.86E-05	0.000391	Schco3|2635333	
scaffold_16	134106	134924	7.24	8.03	5.34	2.69	1.90E-05	0.000398	Schco3|2644327	
scaffold_18	140831	141914	9.47	9.12	9.75	-0.63	1.91E-05	0.000398	Schco3|2645588	
scaffold_11	1573359	1575377	10.04	10.28	9.75	0.53	1.91E-05	0.000398	Schco3|2639451	
scaffold_5	471194	472008	7.32	8.03	5.85	2.17	1.98E-05	0.000412	Schco3|2246556	
scaffold_11	1677967	1679245	9.72	9.96	9.42	0.54	2.01E-05	0.000416	Schco3|2553610	
scaffold_11	569061	569457	5.72	6.65	2.26	4.39	2.15E-05	0.000444	Schco3|2552688	
scaffold_11	240938	243446	10.5	10.77	10.17	0.61	2.16E-05	0.000445		
scaffold_14	928469	930695	9.71	10	9.34	0.66	2.19E-05	0.000448	Schco3|2556751	Schco3|2717811
scaffold_1	3785954	3788084	10.54	10.83	10.19	0.63	2.20E-05	0.00045	Schco3|2608339	
scaffold_13	942738	943482	7.49	8.05	6.58	1.47	2.36E-05	0.00048		
scaffold_2	1351629	1352917	9.63	9.89	9.31	0.59	2.42E-05	0.00049	Schco3|2610398	
scaffold_10	924025	928439	10.57	10.77	10.33	0.44	2.50E-05	0.000504	Schco3|2671052	
scaffold_6	1834527	1837761	10.07	10.35	9.73	0.62	2.53E-05	0.000509	Schco3|2451399	Schco3|2542750
scaffold_2	1664166	1664973	6.5	7.33	4.32	3.01	2.56E-05	0.000513	Schco3|2610764	
scaffold_13	398887	400926	10.02	10.35	9.59	0.76	2.56E-05	0.000513	Schco3|2517503	
scaffold_4	1638746	1639099	4.91	5.89	-0.56	6.45	2.58E-05	0.000514	Schco3|2618755	
scaffold_2	3493403	3494875	9.59	9.9	9.2	0.69	2.65E-05	0.000526	Schco3|2613217	
scaffold_10	1647015	1650091	10.61	10.82	10.35	0.47	2.69E-05	0.000533		
scaffold_1	1478566	1479539	8.03	8.48	7.38	1.09	2.92E-05	0.000576	Schco3|2661224	
scaffold_1	3058021	3059274	9.85	10.09	9.57	0.52	3.04E-05	0.000597	Schco3|2607549	
scaffold_3	1719178	1720381	8.84	8.36	9.2	-0.84	3.11E-05	0.00061	Schco3|2597566	
scaffold_9	582673	584117	8.74	9.07	8.31	0.76	3.14E-05	0.000613	Schco3|2670175	

scaffold_8	469644	470266	4.97	-0.29	5.95	-6.24	3.35E-05	0.000651	Schco3|2335105	
scaffold_9	906086	909774	11.07	11.29	10.8	0.49	3.53E-05	0.000685	Schco3|2509551	Schco3|2703700
scaffold_12	408006	409458	8.51	8.97	7.84	1.13	3.59E-05	0.000693	Schco3|2672114	
scaffold_6	348445	349040	5.99	6.91	2.82	4.09	3.77E-05	0.000723	Schco3|2678116	
scaffold_10	658359	661634	10.42	10.63	10.17	0.46	3.77E-05	0.000723	Schco3|2636554	
scaffold_17	228749	229796	8.86	9.26	8.29	0.97	3.86E-05	0.000738	Schco3|2645038	
scaffold_16	287262	289802	11.25	11.43	11.05	0.39	3.89E-05	0.000741		
scaffold_16	136274	136918	7.54	8.13	6.51	1.62	3.89E-05	0.000741	Schco3|2644330	
scaffold_10	383123	383841	7.89	8.34	7.22	1.13	3.94E-05	0.000747	Schco3|2512973	
scaffold_6	694650	695163	4.93	-0.29	5.91	-6.2	3.96E-05	0.000748	Schco3|2625433	
scaffold_5	2826857	2827393	6.01	6.91	3.2	3.71	4.24E-05	0.000799	Schco3|2624059	Schco3|2624063
scaffold_20	56994	57588	6.66	7.39	5.11	2.28	4.42E-05	0.000827		
scaffold_11	725570	726438	7.15	5.99	7.78	-1.8	4.42E-05	0.000827	Schco3|2638708	
scaffold_13	830852	831358	5.88	3.58	6.73	-3.15	4.44E-05	0.000829	Schco3|2555613	
scaffold_5	3017769	3020687	10.43	10.66	10.15	0.5	4.49E-05	0.000836	Schco3|2624314	
scaffold_1	2734707	2735673	8.97	8.56	9.29	-0.73	4.73E-05	0.000873	Schco3|2607155	
scaffold_6	2141468	2143000	9.31	9.59	8.97	0.63	4.75E-05	0.000873	Schco3|2579523	
scaffold_3	506517	507590	9.64	9.9	9.32	0.57	4.75E-05	0.000873	Schco3|1199648	
scaffold_8	1897107	1899067	10.29	10.5	10.05	0.45	4.75E-05	0.000873	Schco3|2350414	Schco3|2632529
scaffold_12	472811	474432	9.41	9.09	9.68	-0.59	5.02E-05	0.000919	Schco3|2515857	
scaffold_11	351472	351863	5.75	6.67	2.43	4.25	5.07E-05	0.000926	Schco3|2704900	
scaffold_1	3047197	3048466	9.17	9.52	8.72	0.79	5.10E-05	0.000927	Schco3|2607535	
scaffold_11	206371	207540	9.41	9.73	9	0.73	5.12E-05	0.000928		
scaffold_5	456949	457782	7.92	8.47	7	1.48	5.19E-05	0.000938	Schco3|2621012	
scaffold_8	758968	760023	10.62	10.89	10.28	0.62	5.49E-05	0.000989	Schco3|2631085	Schco3|2631088
scaffold_9	1923695	1924993	9.07	9.38	8.67	0.7	5.56E-05	0.001	Schco3|2635519	
scaffold_1	677459	678851	9.57	9.82	9.27	0.55	5.71E-05	0.00102	Schco3|2604681	
scaffold_5	45098	46440	9.69	9.37	9.95	-0.58	5.84E-05	0.00104	Schco3|2620593	
scaffold_4	1801093	1802127	8.52	8.89	8.03	0.85	5.90E-05	0.00105	Schco3|2676970	
scaffold_6	477736	480597	9.83	9.48	10.12	-0.64	6.32E-05	0.00112	Schco3|2625124	
scaffold_11	864748	866093	8.95	9.28	8.5	0.78	6.33E-05	0.00112	Schco3|1240410	Schco3|1240411
scaffold_6	2370205	2373527	11.21	11.39	11.02	0.37	6.51E-05	0.00115		
scaffold_2	1562986	1563372	4.81	5.79	-0.56	6.35	6.68E-05	0.00118	Schco3|2662880	
scaffold_3	2406784	2408693	9.73	10	9.39	0.6	6.71E-05	0.00118	Schco3|2664588	

scaffold_9	1487879	1488967	9.81	10.04	9.54	0.49	6.85E-05	0.0012	Schco3|2670536	
scaffold_1	95388	96663	9.45	9.78	9.04	0.74	6.88E-05	0.0012	Schco3|2603970	
scaffold_9	1360882	1361944	9.5	9.77	9.17	0.6	6.94E-05	0.00121	Schco3|2549660	
scaffold_18	344540	345325	6.94	7.72	5.13	2.59	7.58E-05	0.00131	Schco3|2673930	
scaffold_9	1106096	1106886	8.44	8.81	7.94	0.87	7.59E-05	0.00131	Schco3|2670385	
scaffold_4	3223873	3224869	9.1	9.4	8.72	0.67	7.75E-05	0.00134	Schco3|2620364	
scaffold_4	2419559	2420878	10.13	10.35	9.86	0.48	7.82E-05	0.00134	Schco3|2536562	
scaffold_4	2774246	2775220	8.48	7.71	8.98	-1.27	7.82E-05	0.00134	Schco3|2699497	
scaffold_6	1057048	1058280	9.01	8.62	9.32	-0.7	7.83E-05	0.00134	Schco3|2688704	
scaffold_5	2005158	2006646	9.64	9.89	9.34	0.55	7.87E-05	0.00134	Schco3|2500748	
scaffold_5	487892	488839	9.09	9.46	8.6	0.86	7.95E-05	0.00135	Schco3|2621053	
scaffold_1	1880516	1881399	4.83	-0.29	5.81	-6.1	8.10E-05	0.00137	Schco3|2485086	
scaffold_5	1112851	1114232	9.17	9.44	8.84	0.6	8.70E-05	0.00147	Schco3|2621873	
scaffold_1	4274012	4275246	8.72	9.13	8.16	0.96	8.73E-05	0.00147	Schco3|2608834	
scaffold_3	1436338	1438219	8.89	9.2	8.5	0.7	8.89E-05	0.00149	Schco3|2614789	
scaffold_14	896241	899702	10.58	10.78	10.35	0.44	8.91E-05	0.00149		
scaffold_11	218807	220490	9.42	9.67	9.12	0.56	8.93E-05	0.00149	Schco3|1238578	
scaffold_5	2891345	2892089	8.74	9.06	8.34	0.72	8.97E-05	0.00149	Schco3|2624153	
scaffold_8	1731112	1732028	8.35	8.75	7.78	0.97	9.03E-05	0.0015	Schco3|1156373	
scaffold_6	543090	543703	6.04	4.08	6.85	-2.77	9.07E-05	0.0015	Schco3|2732293	
scaffold_5	504234	504872	5.38	6.29	2.44	3.85	9.88E-05	0.00163	Schco3|2621071	
scaffold_14	232356	233196	8.46	8.83	7.94	0.89	1.00E-04	0.00165	Schco3|2642631	
scaffold_1	947332	948320	8.7	9.09	8.17	0.91	0.000107	0.00176	Schco3|1146793	
scaffold_6	409661	410695	9.25	9.52	8.91	0.61	0.00011	0.0018	Schco3|2625041	
scaffold_2	1358732	1359424	8.04	8.46	7.46	0.99	0.000112	0.00183	Schco3|2610404	
scaffold_9	482272	483601	9.88	10.1	9.61	0.48	0.000113	0.00184	Schco3|2510448	
scaffold_2	2027111	2027960	8.82	9.14	8.4	0.74	0.000116	0.00189	Schco3|1143694	
scaffold_14	289474	290502	6.21	7.11	3.33	3.78	0.00012	0.00194	Schco3|1106673	
scaffold_4	2765459	2767142	9.74	9.96	9.47	0.49	0.000121	0.00196	Schco3|2699494	
scaffold_4	3124151	3124813	6.48	7.37	3.71	3.66	0.000122	0.00196	Schco3|2747016	
scaffold_3	2455749	2456715	8.53	8.99	7.86	1.12	0.000125	0.00201	Schco3|2533371	
scaffold_7	1514294	1515774	9.46	9.71	9.17	0.54	0.000126	0.00202	Schco3|2629302	
scaffold_4	3460437	3461792	10.01	10.24	9.73	0.51	0.000134	0.00213	Schco3|2495894	
scaffold_6	1589990	1591384	8.94	9.25	8.54	0.72	0.000135	0.00215		

<table>
<tr><td>scaffold_11</td><td>689918</td><td>691151</td><td>9.38</td><td>9.08</td><td>9.62</td><td>-0.54</td><td>0.000142</td><td>0.00226</td><td>Schco3|2638672</td><td></td></tr>
<tr><td>scaffold_7</td><td>1433074</td><td>1434039</td><td>9.43</td><td>9.73</td><td>9.06</td><td>0.67</td><td>0.000146</td><td>0.00232</td><td>Schco3|2702249</td><td></td></tr>
<tr><td>scaffold_13</td><td>836304</td><td>837479</td><td>9.99</td><td>10.24</td><td>9.68</td><td>0.57</td><td>0.000153</td><td>0.00241</td><td>Schco3|2642040</td><td></td></tr>
<tr><td>scaffold_7</td><td>1012810</td><td>1014019</td><td>7.42</td><td>7.99</td><td>6.45</td><td>1.54</td><td>0.000153</td><td>0.00241</td><td>Schco3|2628678</td><td></td></tr>
<tr><td>scaffold_17</td><td>202006</td><td>203334</td><td>9.18</td><td>9.44</td><td>8.85</td><td>0.58</td><td>0.000155</td><td>0.00244</td><td>Schco3|2645018</td><td></td></tr>
<tr><td>scaffold_7</td><td>1389609</td><td>1390260</td><td>7.3</td><td>7.88</td><td>6.3</td><td>1.58</td><td>0.000157</td><td>0.00246</td><td>Schco3|2629162</td><td></td></tr>
<tr><td>scaffold_3</td><td>1567088</td><td>1568281</td><td>9.68</td><td>9.95</td><td>9.36</td><td>0.59</td><td>0.000158</td><td>0.00247</td><td>Schco3|2664270</td><td></td></tr>
<tr><td>scaffold_3</td><td>679230</td><td>680257</td><td>8.31</td><td>8.75</td><td>7.65</td><td>1.1</td><td>0.000162</td><td>0.00253</td><td></td><td></td></tr>
<tr><td>scaffold_6</td><td>1223595</td><td>1224461</td><td>8.31</td><td>8.66</td><td>7.83</td><td>0.83</td><td>0.000164</td><td>0.00254</td><td>Schco3|2504269</td><td>Schco3|2626019</td></tr>
<tr><td>scaffold_14</td><td>240597</td><td>242473</td><td>9.89</td><td>10.11</td><td>9.64</td><td>0.48</td><td>0.000166</td><td>0.00258</td><td>Schco3|2642646</td><td></td></tr>
<tr><td>scaffold_8</td><td>1709303</td><td>1710605</td><td>9.76</td><td>9.99</td><td>9.49</td><td>0.5</td><td>0.000171</td><td>0.00264</td><td>Schco3|2632313</td><td></td></tr>
<tr><td>scaffold_14</td><td>853832</td><td>855189</td><td>9.25</td><td>9.55</td><td>8.87</td><td>0.67</td><td>0.000175</td><td>0.00269</td><td>Schco3|2643281</td><td></td></tr>
<tr><td>scaffold_9</td><td>671978</td><td>673473</td><td>9.66</td><td>9.99</td><td>9.25</td><td>0.74</td><td>0.000182</td><td>0.00279</td><td>Schco3|2703618</td><td></td></tr>
<tr><td>scaffold_9</td><td>813548</td><td>816190</td><td>10.76</td><td>10.99</td><td>10.48</td><td>0.5</td><td>0.000183</td><td>0.0028</td><td>Schco3|2585394</td><td></td></tr>
<tr><td>scaffold_5</td><td>2419708</td><td>2420494</td><td>7.76</td><td>6.69</td><td>8.37</td><td>-1.69</td><td>0.000188</td><td>0.00288</td><td>Schco3|2623455</td><td></td></tr>
<tr><td>scaffold_6</td><td>1067793</td><td>1070894</td><td>10.63</td><td>10.9</td><td>10.31</td><td>0.59</td><td>0.00019</td><td>0.0029</td><td>Schco3|2625871</td><td></td></tr>
<tr><td>scaffold_1</td><td>3904798</td><td>3905851</td><td>8.7</td><td>9.08</td><td>8.16</td><td>0.92</td><td>0.000194</td><td>0.00295</td><td>Schco3|2483083</td><td></td></tr>
<tr><td>scaffold_6</td><td>2227796</td><td>2230202</td><td>9.22</td><td>9.47</td><td>8.91</td><td>0.56</td><td>0.000199</td><td>0.00302</td><td>Schco3|1153316</td><td></td></tr>
<tr><td>scaffold_1</td><td>1811121</td><td>1811823</td><td>8.38</td><td>8.77</td><td>7.85</td><td>0.92</td><td>0.000202</td><td>0.00306</td><td>Schco3|2606050</td><td></td></tr>
<tr><td>scaffold_5</td><td>1837582</td><td>1838739</td><td>9.67</td><td>9.9</td><td>9.41</td><td>0.49</td><td>0.000205</td><td>0.00309</td><td>Schco3|2500638</td><td></td></tr>
<tr><td>scaffold_8</td><td>249426</td><td>249813</td><td>4.94</td><td>5.89</td><td>1.02</td><td>4.88</td><td>0.000207</td><td>0.00311</td><td></td><td></td></tr>
<tr><td>scaffold_3</td><td>3249302</td><td>3251769</td><td>10.13</td><td>10.39</td><td>9.81</td><td>0.58</td><td>0.000207</td><td>0.00312</td><td>Schco3|2616887</td><td></td></tr>
<tr><td>scaffold_1</td><td>4033717</td><td>4035166</td><td>9.67</td><td>9.4</td><td>9.9</td><td>-0.5</td><td>0.000211</td><td>0.00315</td><td>Schco3|2608629</td><td></td></tr>
<tr><td>scaffold_2</td><td>3548149</td><td>3549355</td><td>7.58</td><td>8.06</td><td>6.87</td><td>1.19</td><td>0.000211</td><td>0.00315</td><td>Schco3|2487947</td><td>Schco3|2613276</td></tr>
<tr><td>scaffold_3</td><td>3357976</td><td>3360349</td><td>9.84</td><td>10.12</td><td>9.5</td><td>0.62</td><td>0.000216</td><td>0.00321</td><td></td><td></td></tr>
<tr><td>scaffold_8</td><td>364775</td><td>365978</td><td>9.19</td><td>8.88</td><td>9.44</td><td>-0.56</td><td>0.000216</td><td>0.00321</td><td>Schco3|2334177</td><td>Schco3|2630582</td></tr>
<tr><td>scaffold_13</td><td>108079</td><td>108854</td><td>8.51</td><td>8.84</td><td>8.08</td><td>0.76</td><td>0.00022</td><td>0.00326</td><td>Schco3|2693088</td><td>Schco3|2715873</td></tr>
<tr><td>scaffold_2</td><td>2337465</td><td>2338031</td><td>6.88</td><td>7.56</td><td>5.54</td><td>2.02</td><td>0.000226</td><td>0.00334</td><td>Schco3|2611646</td><td></td></tr>
<tr><td>scaffold_11</td><td>709056</td><td>710198</td><td>9.57</td><td>9.82</td><td>9.27</td><td>0.54</td><td>0.000226</td><td>0.00334</td><td>Schco3|2638691</td><td></td></tr>
<tr><td>scaffold_10</td><td>944561</td><td>945773</td><td>9.41</td><td>9.65</td><td>9.12</td><td>0.52</td><td>0.000228</td><td>0.00336</td><td>Schco3|2704432</td><td></td></tr>
<tr><td>scaffold_1</td><td>235919</td><td>236719</td><td>7.19</td><td>7.77</td><td>6.17</td><td>1.6</td><td>0.000229</td><td>0.00336</td><td>Schco3|2604137</td><td></td></tr>
<tr><td>scaffold_5</td><td>3215935</td><td>3217436</td><td>10.23</td><td>10.48</td><td>9.92</td><td>0.56</td><td>0.000232</td><td>0.00341</td><td>Schco3|2624569</td><td></td></tr>
<tr><td>scaffold_4</td><td>98644</td><td>99741</td><td>9.17</td><td>9.46</td><td>8.8</td><td>0.67</td><td>0.000234</td><td>0.00342</td><td></td><td></td></tr>
<tr><td>scaffold_7</td><td>1785780</td><td>1786745</td><td>8.59</td><td>8.91</td><td>8.19</td><td>0.72</td><td>0.00024</td><td>0.0035</td><td>Schco3|2689773</td><td></td></tr>
</table>

scaffold_11	79264	80257	9.03	9.34	8.62	0.71	0.000243	0.00353		
scaffold_9	1673773	1674502	8.21	8.62	7.64	0.98	0.000248	0.0036		
scaffold_11	264591	266139	9.1	9.38	8.75	0.63	0.000253	0.00366	Schco3|2638251	
scaffold_1	462408	464136	9.44	9.67	9.15	0.53	0.000258	0.00372	Schco3|1179436	
scaffold_5	1790600	1791721	9.08	9.39	8.68	0.71	0.000261	0.00376	Schco3|2622685	Schco3|2622691
scaffold_2	1334808	1335729	8.85	9.16	8.47	0.69	0.000271	0.00389	Schco3|2528585	
scaffold_4	1546634	1547851	9.74	9.98	9.44	0.54	0.000274	0.00392	Schco3|2618683	
scaffold_2	1881388	1882152	8.24	8.59	7.77	0.82	0.000279	0.00398	Schco3|2611030	
scaffold_2	3588776	3589585	7.15	7.68	6.28	1.41	0.00029	0.00414	Schco3|2490227	
scaffold_8	1581974	1583828	10.23	10.45	9.96	0.49	0.000303	0.00431	Schco3|2632147	
scaffold_1	1982991	1984579	9.52	9.75	9.23	0.52	0.000306	0.00435	Schco3|2525011	
scaffold_8	1297605	1298508	8.62	8.92	8.23	0.69	0.000309	0.00437	Schco3|2508427	
scaffold_5	701543	704000	10.08	10.27	9.85	0.42	0.000312	0.0044	Schco3|1165624	
scaffold_14	106442	107539	8.07	8.57	7.3	1.27	0.000313	0.00441	Schco3|2642542	
scaffold_5	56712	57992	8.31	8.67	7.82	0.85	0.000322	0.00452		
scaffold_9	1947868	1949094	9.5	9.76	9.19	0.56	0.000345	0.00484	Schco3|2510193	
scaffold_6	267062	268616	10.11	10.31	9.88	0.43	0.000349	0.00488	Schco3|85278	
scaffold_16	372148	373146	7.8	7.17	8.23	-1.06	0.000349	0.00488	Schco3|2644613	
scaffold_4	1843259	1844390	9.66	9.9	9.37	0.53	0.000351	0.00489	Schco3|2496541	
scaffold_5	2276153	2276750	8.03	8.45	7.46	0.99	0.000361	0.00502	Schco3|2576356	
scaffold_17	400181	401747	10.24	10.42	10.02	0.41	0.000362	0.00502	Schco3|2645246	
scaffold_7	1136912	1138064	9.61	9.83	9.35	0.48	0.000369	0.0051	Schco3|1154254	
scaffold_2	1336679	1339036	10.15	9.92	10.34	-0.43	0.00037	0.0051	Schco3|2610379	Schco3|2610381
scaffold_4	3438274	3440586	10.26	10.45	10.05	0.4	0.000373	0.00513	Schco3|1149721	Schco3|2620492
scaffold_2	1715737	1718115	10.47	10.66	10.24	0.43	0.000375	0.00514	Schco3|2488193	
scaffold_3	2712319	2713233	8.11	7.58	8.49	-0.91	0.000378	0.00518	Schco3|2616301	
scaffold_14	113694	114380	8.12	8.55	7.49	1.07	0.000382	0.00521		
scaffold_1	2178397	2181030	10.03	10.25	9.76	0.48	0.000384	0.00524		
scaffold_6	535025	535982	8.69	8.99	8.31	0.69	0.00039	0.0053	Schco3|2625215	
scaffold_5	679795	681803	10.8	11.03	10.53	0.5	0.000393	0.00533	Schco3|2621301	Schco3|2621304
scaffold_10	636387	636982	6.46	7.22	4.71	2.51	0.000394	0.00534	Schco3|2670971	
scaffold_16	218024	219759	10.03	10.29	9.71	0.58	4.00E-04	0.0054	Schco3|2125341	
scaffold_5	1130558	1131302	7.79	8.28	7.03	1.25	0.000402	0.00541	Schco3|2621898	
scaffold_2	425049	427634	10.99	11.22	10.73	0.49	0.000403	0.00542	Schco3|2609408	

scaffold_12	581507	582168	6.41	7.29	3.75	3.54	0.000406	0.00544		
scaffold_6	847095	848653	9.31	9.62	8.91	0.71	0.000408	0.00547	Schco3|2625582	
scaffold_10	707670	708698	9.31	9.55	9.02	0.53	0.00041	0.00548	Schco3|1159065	
scaffold_1	1804607	1805734	9.85	10.05	9.62	0.43	0.00042	0.00559	Schco3|2606037	
scaffold_1	4447425	4449304	9.38	9.64	9.06	0.58	0.00042	0.00559	Schco3|2045557	Schco3|2482047
scaffold_6	1225326	1226564	8.35	8.73	7.82	0.91	0.00043	0.0057		
scaffold_5	1672597	1673871	9.57	9.29	9.81	-0.52	0.000431	0.00571	Schco3|2622520	
scaffold_17	377482	377876	4.61	-0.29	5.58	-5.87	0.000438	0.00578	Schco3|2594459	
scaffold_8	1943977	1945174	9	8.64	9.29	-0.65	0.000438	0.00578	Schco3|2351130	
scaffold_1	3053746	3057370	10.99	10.82	11.15	-0.32	0.000444	0.00584	Schco3|2562706	
scaffold_1	2272072	2273776	10.11	10.38	9.77	0.61	0.000447	0.00586	Schco3|2025596	
scaffold_6	1203640	1205229	9.58	9.8	9.32	0.48	0.000453	0.00593	Schco3|82290	
scaffold_18	337320	338519	9.92	9.68	10.12	-0.44	0.000466	0.00608	Schco3|2694296	
scaffold_9	951182	954444	11.07	11.22	10.91	0.31	0.000467	0.00608	Schco3|2509648	Schco3|2510172
scaffold_5	1792726	1793667	8.65	9.02	8.15	0.87	0.000469	0.0061	Schco3|2622693	
scaffold_5	229634	230034	5.1	6.03	1.6	4.43	0.00047	0.0061		
scaffold_2	645592	648149	10.18	9.86	10.44	-0.59	0.000472	0.00611	Schco3|2609610	Schco3|2743920
scaffold_6	1817685	1818486	7.83	8.23	7.27	0.95	0.000478	0.00617	Schco3|2626720	
scaffold_10	1881228	1881597	4.56	-0.29	5.54	-5.83	0.000485	0.00625	Schco3|2637962	
scaffold_8	681977	683406	9.71	9.95	9.42	0.53	0.000497	0.00639	Schco3|257784	
scaffold_16	400363	403615	10.73	10.93	10.51	0.42	0.000499	0.0064		
scaffold_6	52566	54420	8.87	9.16	8.5	0.66	5.00E-04	0.0064	Schco3|2624760	
scaffold_14	1062859	1065672	10.49	10.66	10.29	0.37	0.000503	0.00642	Schco3|2116266	Schco3|2116292
scaffold_8	607595	607960	4.47	5.44	-0.56	6	0.000508	0.00648	Schco3|2630897	
scaffold_4	1479244	1480482	9.77	9.51	9.99	-0.48	0.000509	0.00648	Schco3|54219	
scaffold_6	976217	977050	8.59	8.92	8.17	0.75	0.00053	0.00673	Schco3|2625740	
scaffold_3	1559606	1560617	9.35	9.62	9.01	0.62	0.000531	0.00673	Schco3|256471	
scaffold_1	53845	54629	8.58	8.92	8.13	0.79	0.000547	0.00689	Schco3|2603938	
scaffold_4	243510	245039	10.11	10.34	9.84	0.49	0.000547	0.00689	Schco3|2617245	
scaffold_2	3495492	3496804	9.69	9.93	9.41	0.52	0.000559	0.00704	Schco3|2174313	
scaffold_3	693832	694980	9.28	9.53	8.97	0.56	0.000569	0.00715	Schco3|2493556	
scaffold_6	2404473	2405769	9.39	9.62	9.12	0.5	0.000573	0.00718	Schco3|2627391	
scaffold_1	3250440	3252277	9.72	9.92	9.49	0.43	0.000577	0.00721	Schco3|2607767	
scaffold_8	1513347	1514698	9.91	9.68	10.11	-0.43	0.00058	0.00723	Schco3|2508511	

scaffold_18	53244	54224	8.09	7.46	8.53	-1.07	6.00E-04	0.00747		
scaffold_7	196661	198979	10.29	10.46	10.08	0.38	0.000604	0.0075	Schco3|2627726	
scaffold_7	953877	955063	8.64	8.18	8.99	-0.81	0.000612	0.00759	Schco3|2314501	
scaffold_8	128095	128884	8.05	8.45	7.5	0.95	0.000619	0.00766	Schco3|2331944	
scaffold_2	2774091	2775442	8.88	9.16	8.52	0.64	0.000628	0.00775	Schco3|2566871	
scaffold_6	1444536	1446442	10.65	10.92	10.33	0.58	0.000644	0.00793	Schco3|2626273	
scaffold_8	1386316	1386990	7.17	7.7	6.32	1.38	0.000651	0.00801	Schco3|2583540	
scaffold_6	1804986	1805540	6.87	7.44	5.93	1.51	0.00066	0.0081	Schco3|2701543	
scaffold_6	1823484	1824609	8.76	9.14	8.26	0.88	0.000661	0.0081	Schco3|2501753	
scaffold_5	2430023	2432956	10.65	10.44	10.84	-0.4	0.000668	0.00817	Schco3|2747928	
scaffold_5	444354	446285	9.6	9.81	9.35	0.46	0.000697	0.00849	Schco3|1194601	
scaffold_9	1705883	1706799	8.03	8.4	7.53	0.87	0.000714	0.00868	Schco3|2635219	
scaffold_6	1217267	1218479	9.34	9.6	9.02	0.58	0.000715	0.00868	Schco3|2542105	
scaffold_7	1591119	1592316	7.09	8.05	2.82	5.23	0.00072	0.00872		
scaffold_6	1525846	1526209	4.5	-0.29	5.47	-5.76	0.000734	0.00888	Schco3|2667966	
scaffold_7	1090753	1092443	9.88	10.07	9.65	0.42	0.000737	0.0089	Schco3|1127222	
scaffold_2	3165997	3167333	9.69	9.98	9.32	0.67	0.000743	0.00895	Schco3|2612816	
scaffold_5	654805	655457	6.58	7.21	5.44	1.77	0.000752	0.00904	Schco3|2538036	
scaffold_5	2427059	2428073	8.71	9.01	8.32	0.69	0.000772	0.00926	Schco3|2623466	
scaffold_9	1816275	1817676	9.68	9.96	9.34	0.62	0.000791	0.00947	Schco3|2635378	
scaffold_2	1706310	1707812	10.11	9.87	10.31	-0.43	0.000801	0.00956	Schco3|2662940	
scaffold_5	1387687	1388988	9.53	9.74	9.29	0.45	0.000822	0.00979	Schco3|2622212	
scaffold_14	393984	395250	9.43	9.65	9.18	0.48	0.000827	0.00983	Schco3|2642828	
scaffold_4	2164214	2165949	10.05	9.84	10.23	-0.39	0.000835	0.00991	Schco3|2496272	Schco3|2619216
scaffold_13	847476	848302	7.53	8	6.84	1.15	0.000856	0.0101	Schco3|2642051	
scaffold_4	2919049	2919891	8.08	8.49	7.49	1	0.00086	0.0102	Schco3|2620109	Schco3|2620112
scaffold_2	2348094	2349274	7.24	6.42	7.75	-1.33	0.000864	0.0102	Schco3|2611665	
scaffold_6	2211491	2212547	9.46	9.7	9.18	0.53	0.000872	0.0103	Schco3|1181112	
scaffold_5	1149663	1150640	9.37	8.99	9.67	-0.68	0.000875	0.0103	Schco3|2498924	
scaffold_3	2149510	2150082	7.08	6.24	7.61	-1.38	0.000907	0.0106	Schco3|1093059	
scaffold_4	2424610	2427641	10.2	10.39	9.97	0.42	0.000908	0.0106	Schco3|2495855	Schco3|2686852
scaffold_9	602396	603580	8.85	8.52	9.13	-0.61	0.000922	0.0108	Schco3|2511180	
scaffold_1	323391	325422	10.61	10.82	10.37	0.44	0.000925	0.0108	Schco3|2604244	
scaffold_4	2867765	2868158	4.36	5.34	-0.56	5.9	0.000927	0.0108		

scaffold_13	1134249	1135282	8.74	9.02	8.4	0.62	0.000933	0.0108	Schco3|2642463	
scaffold_13	142321	144387	10.93	11.14	10.68	0.45	0.000937	0.0109	Schco3|1206404	
scaffold_2	2643972	2645062	9.48	9.69	9.24	0.46	0.00094	0.0109	Schco3|2612072	Schco3|2612073
scaffold_12	228397	231431	10.69	10.85	10.51	0.34	0.000942	0.0109	Schco3|1161064	
scaffold_7	1219441	1220872	9.58	9.79	9.34	0.45	0.000944	0.0109	Schco3|2628974	
scaffold_5	1958246	1959553	9.48	9.7	9.22	0.48	0.000958	0.011	Schco3|2622814	
scaffold_1	4058924	4059828	7.65	8.18	6.82	1.36	0.000983	0.0113	Schco3|2522904	
scaffold_4	1223226	1224426	9.86	10.08	9.59	0.49	0.000989	0.0113	Schco3|2686461	
scaffold_5	2608975	2610270	9.38	9.13	9.6	-0.47	0.00099	0.0113	Schco3|2623746	
scaffold_6	1284124	1285250	8.94	9.25	8.54	0.71	0.00101	0.0115	Schco3|2501779	
scaffold_5	2576591	2577773	9.47	9.68	9.22	0.47	0.00101	0.0115	Schco3|2623701	
scaffold_3	1112984	1114428	9.62	9.86	9.33	0.53	0.00102	0.0116	Schco3|2614499	
scaffold_2	1634806	1635680	7.79	8.18	7.26	0.92	0.00103	0.0117	Schco3|1082935	
scaffold_18	207723	209010	9.43	9.18	9.64	-0.46	0.00103	0.0117	Schco3|2521520	
scaffold_3	2067065	2069261	10.13	9.9	10.32	-0.42	0.00106	0.012	Schco3|2615524	
scaffold_16	239749	240777	8.89	9.14	8.57	0.57	0.00106	0.012	Schco3|2644468	
scaffold_11	677561	678320	6.96	7.52	6.03	1.49	0.00107	0.012	Schco3|1160309	Schco3|2069651
scaffold_17	412915	415393	9.74	9.96	9.48	0.48	0.00107	0.012	Schco3|71133	
scaffold_10	1760969	1762310	9.57	9.77	9.33	0.44	0.00108	0.0121	Schco3|2511858	
scaffold_5	2256571	2257786	8.73	9.02	8.39	0.63	0.00109	0.0122	Schco3|2539827	
scaffold_3	3432934	3433744	8.01	8.45	7.37	1.08	0.0011	0.0123		
scaffold_8	661600	662709	9.2	9.43	8.93	0.49	0.00111	0.0123	Schco3|2630977	
scaffold_3	2084381	2085648	9.7	9.89	9.48	0.41	0.00111	0.0124	Schco3|75401	
scaffold_1	1796171	1797762	9.13	9.39	8.82	0.57	0.00111	0.0124	Schco3|2485669	
scaffold_4	3014370	3015352	9.21	9.43	8.95	0.49	0.00112	0.0124		
scaffold_9	1049385	1050074	7.5	6.84	7.96	-1.11	0.00113	0.0125	Schco3|2634287	
scaffold_10	655021	655989	7.2	6.29	7.75	-1.46	0.00113	0.0125	Schco3|2636549	
scaffold_12	852461	853851	9.76	9.54	9.95	-0.41	0.00113	0.0125	Schco3|2640812	
scaffold_2	1575264	1577183	10.05	10.23	9.84	0.39	0.00114	0.0125	Schco3|2565421	
scaffold_5	825560	826040	5.93	4.36	6.66	-2.3	0.00114	0.0125	Schco3|1220509	
scaffold_1	2114112	2115229	9.49	9.71	9.23	0.49	0.00115	0.0126	Schco3|2485498	
scaffold_6	613224	615442	10.4	10.6	10.18	0.42	0.00117	0.0128	Schco3|2625339	
scaffold_2	2862472	2863733	8.47	8.77	8.1	0.67	0.00118	0.0129	Schco3|2612383	
scaffold_9	1011547	1013016	9.26	9.52	8.96	0.56	0.0012	0.0131	Schco3|2549267	

scaffold_10	957065	958262	9.74	9.94	9.51	0.44	0.00121	0.0131	Schco3|2671068	
scaffold_1	3598074	3599116	9.32	9.54	9.05	0.49	0.00124	0.0134	Schco3|2608143	
scaffold_8	827261	828116	7.05	7.65	6.01	1.65	0.00127	0.0138	Schco3|2582924	
scaffold_2	1129278	1130497	9.4	9.66	9.08	0.58	0.00127	0.0138	Schco3|2486592	
scaffold_3	1582475	1583627	8.49	8.78	8.13	0.65	0.00127	0.0138	Schco3|2614939	
scaffold_4	1955942	1956993	9.32	9.53	9.07	0.47	0.0013	0.014	Schco3|2699217	
scaffold_13	92608	93621	6.26	4.9	6.94	-2.04	0.00132	0.0142	Schco3|1193821	
scaffold_7	1952487	1953801	9.44	9.65	9.2	0.45	0.00132	0.0142	Schco3|2629781	
scaffold_1	413212	414185	7.97	8.37	7.43	0.94	0.00133	0.0143	Schco3|2004062	Schco3|2604352
scaffold_1	1401508	1402553	9.15	9.45	8.77	0.68	0.00135	0.0144	Schco3|2605520	
scaffold_3	2905966	2907404	9.43	9.14	9.67	-0.53	0.00135	0.0144	Schco3|2616501	
scaffold_2	381948	383191	9.75	9.97	9.5	0.46	0.00136	0.0145	Schco3|2609373	
scaffold_18	381770	383135	9.48	9.72	9.19	0.52	0.00138	0.0147	Schco3|2645865	
scaffold_8	1220749	1222482	10.28	10.09	10.44	-0.36	0.00138	0.0147	Schco3|2343794	Schco3|2631712
scaffold_6	2292462	2294625	9.31	9.02	9.55	-0.52	0.00138	0.0147	Schco3|1304977	
scaffold_2	2622329	2623635	10.23	10.44	9.99	0.45	0.0014	0.0148	Schco3|2663317	
scaffold_9	1591912	1594924	11.06	11.22	10.87	0.35	0.0014	0.0148	Schco3|2586217	Schco3|2635034
scaffold_10	1456876	1457912	9.41	9.62	9.16	0.46	0.00144	0.0152	Schco3|2512014	
scaffold_1	13848	17773	11.16	11.31	11.01	0.3	0.00145	0.0153	Schco3|1120444	Schco3|2603898
scaffold_14	120734	122560	10.16	10.35	9.95	0.4	0.00146	0.0153	Schco3|2642553	
scaffold_7	960151	961863	8.74	9.02	8.39	0.63	0.00147	0.0154	Schco3|2628620	
scaffold_1	1228065	1229809	10.09	10.27	9.88	0.4	0.00148	0.0155	Schco3|2605307	Schco3|2605313
scaffold_8	1736124	1736671	7.28	7.76	6.55	1.21	0.00148	0.0155	Schco3|2703146	
scaffold_5	3056239	3058120	9.38	9.61	9.11	0.5	0.00149	0.0156	Schco3|2624363	
scaffold_4	1202479	1203911	9.04	9.28	8.75	0.54	0.00152	0.0158	Schco3|2618252	
scaffold_11	1211152	1212603	10.3	10.52	10.03	0.49	0.00153	0.0159	Schco3|2553185	
scaffold_12	32593	35039	11	11.17	10.79	0.38	0.00154	0.016	Schco3|2553746	
scaffold_10	1129291	1130497	9.53	9.29	9.73	-0.44	0.00155	0.016	Schco3|2636997	
scaffold_5	1538061	1538969	9.12	9.37	8.83	0.54	0.00155	0.016	Schco3|1165921	
scaffold_9	1639725	1640378	6.5	7.1	5.44	1.66	0.00158	0.0163	Schco3|2372352	Schco3|2670601
scaffold_11	284740	285421	6.9	7.43	6.08	1.35	0.0016	0.0165	Schco3|2513919	
scaffold_4	3441057	3442407	9.37	9.6	9.09	0.51	0.00162	0.0167	Schco3|2620505	Schco3|2699688
scaffold_5	2896204	2897218	8.64	8.91	8.31	0.61	0.00163	0.0168	Schco3|2624161	Schco3|2624163
scaffold_5	2412987	2413712	7.23	6.48	7.73	-1.25	0.00165	0.0169	Schco3|2623449	

scaffold_5	393954	395086	6.85	7.43	5.86	1.57	0.00167	0.0171	Schco3|2447881	Schco3|2620946
scaffold_7	1734045	1734844	7.63	8.04	7.07	0.97	0.0017	0.0174	Schco3|2506202	Schco3|2629570
scaffold_3	1149964	1151220	9.42	9.63	9.17	0.46	0.00173	0.0177	Schco3|2568673	
scaffold_3	1862840	1863958	8.78	9.03	8.46	0.57	0.00175	0.0178	Schco3|2615260	
scaffold_2	1322433	1323551	9.22	9.46	8.93	0.53	0.00176	0.0179	Schco3|2490454	
scaffold_2	3123459	3125110	10.02	10.23	9.78	0.44	0.00177	0.018	Schco3|2612754	Schco3|2612756
scaffold_10	493404	493910	5.52	2.77	6.41	-3.64	0.00178	0.018	Schco3|2636333	
scaffold_5	2298941	2299314	5.51	6.32	3.49	2.83	0.00178	0.018	Schco3|2539870	
scaffold_15	576659	577593	8.96	9.22	8.66	0.56	0.00178	0.018		
scaffold_1	2554182	2555766	10.58	10.75	10.4	0.34	0.00179	0.018	Schco3|2606937	
scaffold_2	2523696	2524910	9.45	9.65	9.2	0.45	0.0018	0.0182	Schco3|1230336	Schco3|2611888
scaffold_9	1233020	1234272	9.96	10.17	9.7	0.47	0.00182	0.0183		
scaffold_9	1093190	1095658	10.24	10.46	9.98	0.47	0.00183	0.0184	Schco3|2634355	Schco3|58871
scaffold_12	622837	624311	9.93	10.15	9.65	0.5	0.00184	0.0184	Schco3|2640518	
scaffold_2	2377218	2379835	10.03	10.2	9.83	0.38	0.00184	0.0184	Schco3|2611704	
scaffold_7	664090	664997	7.37	7.98	6.3	1.68	0.00185	0.0185	Schco3|2628264	
scaffold_2	1077666	1078708	9.27	9.57	8.9	0.68	0.00186	0.0185	Schco3|1196424	
scaffold_9	405748	407322	9.95	10.12	9.74	0.38	0.00187	0.0186	Schco3|2633443	Schco3|2633445
scaffold_2	1860806	1861898	9.95	10.12	9.75	0.37	0.00188	0.0187	Schco3|1080583	Schco3|2611006
scaffold_5	175999	177095	9.16	9.49	8.75	0.73	0.00194	0.0193	Schco3|2243453	Schco3|2620649
scaffold_2	705761	708465	10.55	10.77	10.29	0.47	0.00197	0.0195	Schco3|2743933	
scaffold_1	629048	630218	8.85	9.81	4.43	5.38	0.00199	0.0196	Schco3|2742365	
scaffold_5	1036871	1038250	9.28	9.53	8.97	0.56	0.00202	0.0199	Schco3|1221170	
scaffold_9	1581165	1582146	8.56	8.88	8.14	0.75	0.00202	0.0199	Schco3|2510275	
scaffold_7	1697939	1698914	9.15	9.38	8.87	0.51	0.00203	0.0199	Schco3|2545061	
scaffold_16	269969	271564	9.78	9.97	9.55	0.43	0.00208	0.0204	Schco3|2644507	
scaffold_1	197158	198357	8.84	9.11	8.52	0.58	0.00209	0.0204	Schco3|2604109	
scaffold_8	738248	740883	10.65	10.81	10.48	0.33	0.00209	0.0205	Schco3|2631069	
scaffold_13	639831	640669	5.95	3.84	6.78	-2.94	0.0021	0.0205	Schco3|2641873	
scaffold_3	632499	633573	9.48	9.68	9.26	0.42	0.00212	0.0206	Schco3|1122822	
scaffold_2	1687907	1689431	10.04	10.23	9.82	0.41	0.00212	0.0206	Schco3|2610799	
scaffold_9	1103240	1104664	9.61	9.82	9.35	0.48	0.00213	0.0207	Schco3|2585708	Schco3|2585710
scaffold_1	3356260	3356913	6.69	7.26	5.75	1.5	0.00214	0.0207	Schco3|2607871	
scaffold_15	187608	189190	9.81	9.6	9.99	-0.4	0.00215	0.0208	Schco3|2681670	

scaffold_2	1649786	1652458	10.77	10.91	10.61	0.3	0.00215	0.0208	Schco3|2153236	Schco3|2528923
scaffold_2	3156619	3158174	9.33	9.58	9.03	0.55	0.00224	0.0216	Schco3|1193511	Schco3|2612806
scaffold_3	587930	589185	9.68	9.89	9.44	0.44	0.00226	0.0218	Schco3|2614017	
scaffold_8	377779	378297	5.77	6.55	3.96	2.59	0.00229	0.022	Schco3|2630602	
scaffold_5	2222716	2224675	9.67	9.88	9.43	0.46	0.0023	0.022	Schco3|2500037	
scaffold_6	458049	459069	8.75	9.02	8.41	0.61	0.00231	0.0221	Schco3|2502058	
scaffold_5	2841663	2842751	8.7	8.39	8.96	-0.57	0.00233	0.0223		
scaffold_10	640153	641170	8.42	8.05	8.72	-0.67	0.00234	0.0223		
scaffold_3	1730528	1731358	7.61	8	7.09	0.91	0.00235	0.0223	Schco3|2615099	
scaffold_13	584020	585042	8.66	8.99	8.25	0.74	0.00235	0.0223	Schco3|2555469	Schco3|2641815
scaffold_8	715177	716146	8.85	9.1	8.53	0.58	0.00238	0.0227		
scaffold_11	611657	612764	7.93	8.29	7.44	0.84	0.00239	0.0227	Schco3|2638565	Schco3|2692297
scaffold_3	1390355	1391336	9.51	9.72	9.25	0.47	0.0024	0.0227	Schco3|1123248	
scaffold_6	2524733	2526404	9.98	10.23	9.68	0.54	0.00242	0.0229	Schco3|2627560	
scaffold_8	1890290	1893210	10.26	10.05	10.45	-0.4	0.00244	0.0231	Schco3|2632514	Schco3|2632518
scaffold_9	1606987	1607915	7.47	7.91	6.81	1.1	0.00249	0.0235	Schco3|2586235	
scaffold_2	1237603	1239075	9.69	9.9	9.45	0.45	0.00251	0.0237	Schco3|2565065	
scaffold_8	1868701	1872772	11.3	11.42	11.16	0.26	0.00256	0.0241	Schco3|2632493	
scaffold_3	2283423	2284118	8.04	8.37	7.62	0.75	0.0026	0.0244	Schco3|2198700	
scaffold_6	948040	949399	9.37	9.58	9.13	0.45	0.0026	0.0244	Schco3|2625693	
scaffold_2	2489222	2490854	10.35	10.53	10.13	0.4	0.00265	0.0248	Schco3|2611855	
scaffold_8	1635255	1636813	9.25	9.49	8.98	0.51	0.00266	0.0248	Schco3|2632240	
scaffold_6	37979	38762	8.25	7.84	8.57	-0.72	0.00267	0.0249		
scaffold_12	354504	355824	9	8.68	9.26	-0.59	0.00272	0.0253	Schco3|2752466	
scaffold_13	968052	969492	8.94	8.61	9.21	-0.6	0.00272	0.0253	Schco3|2753029	
scaffold_1	2038586	2040530	10.46	10.29	10.62	-0.33	0.00273	0.0253	Schco3|2606391	
scaffold_1	1460548	1461819	9.97	10.14	9.77	0.36	0.00276	0.0256	Schco3|2605607	
scaffold_4	1383582	1384364	8.34	8.63	7.96	0.67	0.00277	0.0256	Schco3|2618479	
scaffold_10	1205888	1206907	9.46	9.66	9.22	0.43	0.00277	0.0256	Schco3|2637126	
scaffold_10	684263	685219	9.39	9.58	9.16	0.43	0.00278	0.0257	Schco3|2636577	
scaffold_13	962975	964279	8.12	7.66	8.47	-0.81	0.0028	0.0258	Schco3|2642193	
scaffold_9	475763	477975	10.09	10.26	9.89	0.37	0.00282	0.0259	Schco3|2633538	Schco3|2633542
scaffold_13	476777	478102	9.83	10	9.63	0.37	0.00285	0.0261	Schco3|2555361	Schco3|2641689
scaffold_7	1125721	1128893	11.21	11.39	11	0.39	0.00288	0.0264	Schco3|2628870	

scaffold_13	534620	535973	8.53	8.2	8.8	-0.6	0.00288	0.0264		
scaffold_8	1668945	1669574	6.12	6.77	4.91	1.85	0.0029	0.0265	Schco3|2547500	
scaffold_5	790014	791021	8.13	7.73	8.45	-0.72	0.00291	0.0265	Schco3|2621449	
scaffold_2	3145255	3146707	9.05	9.28	8.79	0.48	0.00295	0.0269	Schco3|2612793	
scaffold_9	1114235	1116004	10.59	10.76	10.39	0.37	0.00296	0.0269	Schco3|2634380	
scaffold_7	2014520	2016558	10.16	10.36	9.93	0.43	0.00297	0.027	Schco3|1057329	Schco3|2735894
scaffold_2	3018928	3021627	10.35	10.17	10.51	-0.34	0.00298	0.027	Schco3|2612594	
scaffold_3	3099833	3100483	6.38	7.18	4.45	2.73	0.00299	0.0271	Schco3|2491912	Schco3|2685994
scaffold_6	1185770	1186941	8.68	8.96	8.34	0.63	0.00299	0.0271	Schco3|2625944	
scaffold_2	2360333	2361049	7.56	7.96	7.03	0.93	0.003	0.0271	Schco3|1116386	
scaffold_14	374195	375127	8.68	8.96	8.34	0.62	0.00302	0.0272	Schco3|2518851	
scaffold_4	344349	346285	10.5	10.7	10.28	0.42	0.00304	0.0274	Schco3|2497373	
scaffold_8	1286725	1287414	7.28	7.71	6.65	1.06	0.00305	0.0274	Schco3|2631792	
scaffold_12	803387	804346	7.19	8.14	3.24	4.91	0.00305	0.0274	Schco3|2088871	
scaffold_1	3658183	3660590	10.81	10.98	10.63	0.35	0.00306	0.0274	Schco3|2608225	Schco3|2608228
scaffold_14	1047291	1048899	9.48	9.73	9.18	0.56	0.00312	0.0279	Schco3|2603352	
scaffold_21	32733	35935	11.1	11.3	10.86	0.44	0.00314	0.0281	Schco3|2646061	
scaffold_2	2874640	2875654	8.87	9.1	8.58	0.52	0.00317	0.0283	Schco3|2488254	
scaffold_5	3137087	3137868	8.07	8.39	7.66	0.73	0.00318	0.0284	Schco3|1142628	
scaffold_12	612680	616674	11.32	11.45	11.18	0.27	0.0032	0.0284	Schco3|2086861	
scaffold_5	2535445	2536874	9.66	9.84	9.45	0.39	0.0032	0.0284	Schco3|2268565	Schco3|2623646
scaffold_7	1634667	1635766	9.92	9.73	10.08	-0.36	0.00323	0.0286	Schco3|2629417	
scaffold_2	1902891	1907126	10.86	11	10.71	0.29	0.00323	0.0286	Schco3|2611066	
scaffold_7	1998022	2001541	10.75	10.93	10.55	0.38	0.00325	0.0288		
scaffold_1	3987170	3988189	9.18	9.41	8.92	0.49	0.00328	0.0289	Schco3|2608591	
scaffold_4	343129	344011	8.4	8.68	8.04	0.64	0.00329	0.029	Schco3|2686181	
scaffold_6	1719077	1720347	9.78	9.95	9.58	0.37	0.00332	0.0292	Schco3|2295872	
scaffold_5	1000442	1001837	9.85	9.65	10.02	-0.36	0.00333	0.0292	Schco3|2621741	
scaffold_1	1347926	1349490	10.01	10.21	9.78	0.42	0.00334	0.0293	Schco3|2605448	
scaffold_1	1606747	1607866	9.54	9.25	9.78	-0.52	0.00339	0.0297	Schco3|2605806	
scaffold_1	1297794	1300167	10.56	10.71	10.4	0.31	0.00342	0.0299	Schco3|2560806	
scaffold_8	1281391	1282430	9.73	9.53	9.91	-0.39	0.00342	0.0299	Schco3|2631786	
scaffold_5	2506578	2509205	11.16	11.35	10.93	0.41	0.00349	0.0304	Schco3|2268131	Schco3|2623596
scaffold_5	1559371	1560489	9.83	10.08	9.53	0.54	0.00349	0.0304	Schco3|2599095	

scaffold_9	1330933	1333774	10.84	10.67	10.99	-0.32	0.00351	0.0305	Schco3|1203519	Schco3|2585972
scaffold_2	2904763	2906775	10.63	10.79	10.44	0.35	0.00351	0.0305	Schco3|2612445	Schco3|2744772
scaffold_1	3165944	3167051	9.71	9.89	9.51	0.39	0.00352	0.0305	Schco3|2483413	
scaffold_10	650336	651303	8.1	7.69	8.42	-0.73	0.00355	0.0308	Schco3|2636544	
scaffold_10	79467	80798	9.36	9.58	9.1	0.48	0.00356	0.0308	Schco3|2635786	
scaffold_13	119180	120062	8.4	8.68	8.04	0.64	0.00357	0.0308	Schco3|2094287	
scaffold_4	906557	907293	8.09	7.58	8.47	-0.9	0.00358	0.0309	Schco3|2698845	
scaffold_5	2989916	2990957	8.56	8.82	8.24	0.57	0.00366	0.0315	Schco3|2624284	
scaffold_5	1720495	1721430	8.99	9.28	8.62	0.67	0.00367	0.0315	Schco3|2747669	
scaffold_1	2451051	2452233	9.33	9.54	9.08	0.46	0.00368	0.0316	Schco3|2525437	
scaffold_6	202241	203441	9.59	9.79	9.36	0.43	0.00373	0.0319	Schco3|2624823	
scaffold_13	324085	325981	10.67	10.83	10.49	0.35	0.0038	0.0325	Schco3|2641517	
scaffold_6	1756170	1756743	6.85	7.36	6.05	1.32	0.00382	0.0326	Schco3|2626646	
scaffold_5	3105623	3107180	10.07	10.24	9.86	0.38	0.00383	0.0326	Schco3|2624428	
scaffold_7	661211	663452	10.54	10.75	10.29	0.46	0.00383	0.0326	Schco3|2628260	
scaffold_7	1344895	1345542	5.84	6.61	4.06	2.55	0.00385	0.0328		
scaffold_5	2762228	2762975	8.38	8.66	8.02	0.64	0.00388	0.033	Schco3|2498147	
scaffold_6	1058732	1060386	10.47	10.68	10.24	0.44	0.0039	0.0331	Schco3|76719	
scaffold_12	1246839	1247400	6.09	6.78	4.75	2.03	0.00391	0.0331	Schco3|2641260	
scaffold_2	1870339	1870839	6.84	7.35	6.04	1.31	0.00392	0.0331	Schco3|2611016	Schco3|2611018
scaffold_9	987158	988583	9.42	9.2	9.6	-0.4	0.00392	0.0331	Schco3|2634209	
scaffold_10	1689405	1691575	9.89	9.71	10.06	-0.35	0.00393	0.0331	Schco3|2637716	
scaffold_1	478626	479054	5.43	6.3	2.82	3.48	0.00395	0.0332	Schco3|2604422	
scaffold_7	912760	915988	10.54	10.37	10.69	-0.32	0.00395	0.0332	Schco3|2314225	Schco3|2702080
scaffold_4	92283	93102	7.49	7.91	6.92	0.99	0.00398	0.0334		
scaffold_8	1600696	1601242	4.2	-0.29	5.16	-5.45	0.00398	0.0334	Schco3|2583780	
scaffold_4	475974	477550	10.08	10.25	9.9	0.34	0.004	0.0335	Schco3|1169257	
scaffold_9	982444	983167	7.74	8.14	7.19	0.95	0.00402	0.0336	Schco3|2634205	
scaffold_2	1822285	1825658	11.03	11.16	10.89	0.27	0.00403	0.0336	Schco3|2696654	
scaffold_2	1226688	1227970	9.5	9.73	9.23	0.5	0.00407	0.0339	Schco3|2720935	
scaffold_7	1421615	1422763	8.98	8.69	9.22	-0.53	0.00407	0.0339	Schco3|2629191	
scaffold_10	694735	696273	9.69	9.95	9.37	0.58	0.0041	0.0339	Schco3|2636589	Schco3|2636591
scaffold_1	1190563	1191166	7.41	7.82	6.82	1	0.0041	0.0339	Schco3|2524109	
scaffold_2	3728150	3729394	8.48	8.78	8.1	0.69	0.0041	0.0339	Schco3|2613571	

scaffold_20	10433	12905	10.35	10.51	10.17	0.34	0.0041	0.0339	Schco3|2603845	
scaffold_7	1268165	1269183	7.25	7.85	6.2	1.65	0.0041	0.0339	Schco3|1213723	
scaffold_1	1001868	1003061	10.08	10.29	9.84	0.45	0.0041	0.0339	Schco3|2605073	Schco3|2742505
scaffold_17	492665	494449	10.15	10.39	9.87	0.52	0.00414	0.0341	Schco3|2645362	
scaffold_6	587327	588217	7.97	8.33	7.5	0.82	0.00415	0.0342	Schco3|2625300	
scaffold_5	2178426	2179899	9.8	10.03	9.54	0.49	0.00416	0.0342	Schco3|2623145	Schco3|2623146
scaffold_1	1485541	1486460	8.7	8.94	8.41	0.54	0.00419	0.0344	Schco3|2605636	
scaffold_17	148202	149506	9.15	9.39	8.86	0.52	0.0042	0.0344	Schco3|2644950	
scaffold_4	933688	934387	6.63	5.68	7.2	-1.52	0.00421	0.0345	Schco3|2617901	
scaffold_4	1206933	1208550	10.17	10.33	9.98	0.35	0.00422	0.0345	Schco3|2535412	Schco3|2571979
scaffold_21	54796	56315	10.34	10.5	10.17	0.33	0.00429	0.035	Schco3|2646083	
scaffold_12	160041	160715	7.35	7.78	6.74	1.04	0.00429	0.035		
scaffold_8	1461231	1464345	11.1	11.24	10.96	0.28	0.00431	0.0351	Schco3|2507043	Schco3|2632006
scaffold_1	3412267	3413929	9.94	9.69	10.15	-0.46	0.00433	0.0352	Schco3|2481145	
scaffold_10	1012650	1013737	9.35	9.04	9.61	-0.57	0.00434	0.0352	Schco3|2671089	
scaffold_4	987055	988062	8.66	8.34	8.92	-0.57	0.00438	0.0355	Schco3|1280903	Schco3|2698877
scaffold_5	282021	283091	9.08	9.32	8.8	0.52	0.00439	0.0355	Schco3|2620780	
scaffold_1	2919687	2920988	9.33	9.08	9.54	-0.45	0.00442	0.0357	Schco3|2032037	Schco3|2607393
scaffold_8	1952881	1955548	9.82	9.6	10.02	-0.43	0.00442	0.0357	Schco3|2632606	
scaffold_8	1784837	1785778	9.09	9.31	8.83	0.49	0.00443	0.0357		
scaffold_9	134016	135113	8.48	8.75	8.14	0.61	0.00448	0.0361	Schco3|2584676	
scaffold_14	575336	576588	8.86	9.1	8.57	0.53	0.00449	0.0361	Schco3|86124	
scaffold_6	1413003	1414648	10.22	10.4	10.03	0.36	0.00449	0.0361	Schco3|2626206	
scaffold_2	2917196	2919427	9.27	9.53	8.97	0.56	0.00453	0.0363	Schco3|2167926	Schco3|2567053
scaffold_15	200426	203045	9.95	10.2	9.65	0.55	0.00456	0.0365	Schco3|2643773	
scaffold_5	1622410	1623015	5.24	3.13	6.07	-2.94	0.00457	0.0365	Schco3|2499350	
scaffold_12	429007	430497	8.53	8.8	8.2	0.6	0.00458	0.0365	Schco3|2602651	
scaffold_2	3353828	3356720	11.12	11.27	10.95	0.32	0.0046	0.0367	Schco3|2487849	
scaffold_10	1581971	1584310	10.12	10.28	9.94	0.34	0.0046	0.0367	Schco3|2637568	
scaffold_10	264197	265060	7.41	7.83	6.81	1.02	0.00462	0.0367	Schco3|2636005	
scaffold_1	1744986	1746850	9.58	9.34	9.79	-0.45	0.00463	0.0367	Schco3|2485397	
scaffold_5	2016485	2017884	8.55	8.86	8.16	0.7	0.00464	0.0368	Schco3|2539542	
scaffold_4	369420	370553	9.43	9.62	9.21	0.41	0.00464	0.0368	Schco3|2212909	
scaffold_12	537709	538350	7.84	8.18	7.39	0.79	0.00468	0.037	Schco3|2085956	

scaffold_12	1222683	1224995	10.15	10.34	9.94	0.4	0.00468	0.037	Schco3|2641214	
scaffold_10	689040	690912	10.15	10.31	9.96	0.35	0.0047	0.0371	Schco3|2636580	
scaffold_5	1778254	1780558	10.43	10.58	10.26	0.32	0.00472	0.0372	Schco3|2622668	Schco3|2622673
scaffold_3	2438761	2439574	9.01	9.24	8.73	0.52	0.00472	0.0372	Schco3|2615974	Schco3|2615976
scaffold_6	2095248	2096623	9.15	8.91	9.36	-0.45	0.00473	0.0372	Schco3|2627062	
scaffold_5	2316788	2319197	10.06	9.83	10.26	-0.44	0.0048	0.0376	Schco3|2623309	Schco3|2623312
scaffold_10	1813920	1815809	10.18	10.33	10	0.33	0.00485	0.038	Schco3|2512368	
scaffold_2	1068946	1072008	10.95	10.75	11.12	-0.37	0.00486	0.038	Schco3|2487593	Schco3|2720828
scaffold_9	1501644	1503635	9.32	9.53	9.09	0.44	0.00486	0.038	Schco3|2634922	
scaffold_4	133481	135751	10.24	10.43	10.03	0.41	0.00489	0.0382	Schco3|2617151	
scaffold_9	235657	236919	9.43	9.61	9.21	0.4	0.0049	0.0382	Schco3|2509917	Schco3|2633289
scaffold_13	1042873	1047965	10.98	11.11	10.83	0.28	0.00492	0.0383	Schco3|2517285	
scaffold_4	66608	68558	9.9	9.68	10.1	-0.42	0.00495	0.0385	Schco3|2495705	
scaffold_5	342220	343298	8.81	9.06	8.51	0.55	0.00498	0.0386	Schco3|1218986	
scaffold_2	3186076	3187204	9.95	10.12	9.75	0.37	0.00498	0.0386	Schco3|2612846	
scaffold_8	1908380	1909055	7.1	6.44	7.55	-1.11	0.00499	0.0386	Schco3|248956	
scaffold_9	1857325	1859588	10.66	10.83	10.46	0.37	0.00502	0.0388	Schco3|2635430	Schco3|2635435
scaffold_14	330746	331495	8.24	8.57	7.82	0.74	0.00506	0.0391	Schco3|2717189	
scaffold_5	3313462	3314714	9.62	9.39	9.81	-0.42	0.00507	0.0391	Schco3|2624691	
scaffold_9	1063601	1066226	10.57	10.37	10.75	-0.38	0.00509	0.0392	Schco3|2634313	Schco3|2634318
scaffold_4	2329529	2330596	9.99	10.16	9.8	0.36	0.00511	0.0393	Schco3|2494295	
scaffold_1	2673760	2674712	9.58	9.81	9.3	0.51	0.00512	0.0393	Schco3|2607089	
scaffold_5	2798135	2800102	9.82	9.63	9.98	-0.35	0.00512	0.0393	Schco3|2688203	
scaffold_10	1138443	1139842	10.02	10.19	9.83	0.35	0.00514	0.0394	Schco3|2637020	
scaffold_18	470683	472401	10.28	10.47	10.06	0.41	0.00519	0.0397	Schco3|2645921	
scaffold_2	2158793	2160705	10.06	10.26	9.83	0.43	0.00522	0.0399	Schco3|2596801	
scaffold_12	1285269	1286030	8.21	8.54	7.78	0.76	0.00523	0.0399	Schco3|2641286	
scaffold_4	2705604	2706198	6.83	7.39	5.87	1.52	0.00525	0.04	Schco3|2746897	
scaffold_8	868277	869817	9.93	10.11	9.73	0.38	0.00528	0.0402		
scaffold_11	1524535	1525884	9.65	9.89	9.35	0.54	0.00529	0.0402	Schco3|2076802	
scaffold_2	2249077	2251492	10.48	10.65	10.3	0.35	0.00533	0.0404	Schco3|2160311	
scaffold_5	2879377	2880210	8.76	9.05	8.4	0.65	0.00533	0.0404	Schco3|2700763	
scaffold_15	180011	181222	9.68	9.89	9.44	0.45	0.00535	0.0405	Schco3|2118324	Schco3|2519741
scaffold_4	2885738	2888106	10.34	10.53	10.12	0.41	0.00542	0.041	Schco3|2472070	Schco3|2665974

scaffold_3	1078739	1080116	9.87	10.1	9.59	0.51	0.00544	0.0411	Schco3|2493117	
scaffold_2	633494	635328	9.83	10.01	9.63	0.38	0.00546	0.0412	Schco3|2467772	Schco3|2488013
scaffold_7	687549	688922	9.53	9.31	9.71	-0.4	0.00548	0.0413	Schco3|2311844	
scaffold_7	794405	796555	10.29	10.13	10.44	-0.31	0.00549	0.0413	Schco3|2628431	Schco3|2668600
scaffold_3	956346	957140	7.04	6.28	7.53	-1.25	0.00554	0.0416	Schco3|2614269	
scaffold_5	2668656	2671235	10.65	10.83	10.43	0.4	0.00556	0.0417	Schco3|2623834	Schco3|9874
scaffold_4	542177	543290	9.19	9.39	8.96	0.43	0.00556	0.0417	Schco3|2617551	
scaffold_7	1163825	1165002	9.46	9.26	9.64	-0.39	0.00558	0.0418	Schco3|2668731	
scaffold_1	2928307	2929823	9.71	9.9	9.49	0.41	0.00561	0.042	Schco3|2032193	Schco3|2607408
scaffold_3	1960722	1963371	10.21	10.36	10.04	0.32	0.00565	0.0422	Schco3|2490724	
scaffold_6	1492725	1493421	7.54	7.97	6.92	1.05	0.00565	0.0422	Schco3|2626326	
scaffold_3	2190071	2191502	10.22	10.41	10.01	0.4	0.00571	0.0425	Schco3|2493743	
scaffold_2	2548252	2549889	9.43	9.66	9.14	0.52	0.00574	0.0427		
scaffold_13	1094044	1095333	8.95	8.68	9.17	-0.49	0.00575	0.0427	Schco3|2592158	
scaffold_9	1195811	1196945	8.1	7.71	8.41	-0.71	0.00576	0.0427	Schco3|1191692	Schco3|2509989
scaffold_12	920863	923487	10.74	10.87	10.58	0.29	0.00585	0.0434	Schco3|2640902	Schco3|2681137
scaffold_5	2983106	2983887	6.26	5	6.92	-1.93	0.00588	0.0435	Schco3|2501258	
scaffold_3	2764279	2765903	10.05	10.21	9.88	0.33	0.00592	0.0437	Schco3|2616339	
scaffold_2	3413926	3414892	8.92	9.17	8.62	0.55	0.00595	0.0439	Schco3|2567612	
scaffold_6	2049743	2051491	10.07	9.9	10.23	-0.33	0.00597	0.044	Schco3|2627009	
scaffold_8	1775244	1777027	9.35	9.09	9.57	-0.47	0.00606	0.0446	Schco3|1173242	Schco3|2601101
scaffold_13	521094	522574	9.6	9.41	9.76	-0.36	0.00606	0.0446	Schco3|2641737	Schco3|2641742
scaffold_5	2300518	2302293	9.94	10.1	9.76	0.34	0.00608	0.0446	Schco3|2623289	
scaffold_7	919706	920928	8.87	9.84	4.47	5.36	0.00611	0.0449		
scaffold_8	74698	75953	8.74	9.03	8.37	0.66	0.00615	0.045	Schco3|2630289	
scaffold_1	1620083	1621303	9.65	9.46	9.81	-0.35	0.00618	0.0453	Schco3|2605822	
scaffold_5	459689	461295	10.43	10.58	10.26	0.31	0.0062	0.0453	Schco3|2501591	Schco3|2621017
scaffold_5	1549631	1550296	6.09	6.87	4.24	2.64	0.00621	0.0453	Schco3|2468228	
scaffold_8	813456	815196	10.14	10.33	9.91	0.42	0.00622	0.0453	Schco3|2509259	
scaffold_6	278396	279072	7.32	6.7	7.75	-1.05	0.00626	0.0456	Schco3|2279054	
scaffold_9	98681	100223	9.03	9.32	8.67	0.64	0.00631	0.0459	Schco3|2633155	
scaffold_1	1455058	1457170	9.92	9.73	10.08	-0.35	0.00635	0.0461	Schco3|2605593	Schco3|2605597
scaffold_6	424703	426329	8.73	8.37	9.02	-0.65	0.00638	0.0463	Schco3|2502848	Schco3|2577799
scaffold_3	2784179	2785611	9.75	9.91	9.56	0.36	0.0064	0.0464	Schco3|2685882	

scaffold_3	560770	562386	7.83	8.19	7.35	0.84	0.00641	0.0464		
scaffold_16	33352	34504	9.29	9.48	9.08	0.4	0.00643	0.0465	Schco3|2123167	
scaffold_1	3849960	3851278	8.77	9.02	8.46	0.55	0.00651	0.047	Schco3|2608404	
scaffold_3	1500628	1502403	9.7	9.87	9.5	0.37	0.00652	0.047	Schco3|2614856	
scaffold_2	3325851	3328140	10.29	10.45	10.11	0.34	0.00653	0.047	Schco3|2613044	
scaffold_4	1812901	1816547	10.98	11.11	10.84	0.27	0.00654	0.047	Schco3|2665598	
scaffold_6	2284789	2286408	8.68	8.93	8.37	0.56	0.00654	0.047	Schco3|2668239	
scaffold_8	1374692	1375934	8.57	8.16	8.9	-0.74	0.00657	0.0471	Schco3|1356221	
scaffold_2	1249292	1251379	9.49	9.66	9.29	0.37	0.00658	0.0471	Schco3|2488787	Schco3|2610278
scaffold_14	360355	361454	9.54	9.72	9.34	0.38	0.00659	0.0472	Schco3|2518817	Schco3|2642788
scaffold_7	819030	819773	7.82	8.18	7.35	0.83	0.00663	0.0473	Schco3|2313326	
scaffold_1	4074777	4075831	9.19	9.42	8.9	0.52	0.00663	0.0473		
scaffold_12	1046683	1048050	9.89	10.05	9.71	0.34	0.00666	0.0475	Schco3|2517048	
scaffold_5	220988	222736	10	10.18	9.8	0.38	0.00669	0.0476	Schco3|2620718	
scaffold_7	493387	494267	7.9	8.22	7.48	0.74	0.0067	0.0477	Schco3|2628084	
scaffold_4	2978622	2980282	10.5	10.64	10.34	0.3	0.00673	0.0478	Schco3|2620175	
scaffold_3	1734389	1737400	11.08	10.94	11.21	-0.27	0.0068	0.0483	Schco3|2615106	Schco3|2615107
scaffold_1	4013668	4014915	10.1	10.33	9.84	0.49	0.00681	0.0483	Schco3|2482122	
scaffold_1	3620631	3621825	9.7	9.87	9.52	0.35	0.00686	0.0486	Schco3|2608180	Schco3|2608184
scaffold_4	969018	971392	10.73	10.86	10.59	0.27	0.00687	0.0486	Schco3|2617962	
scaffold_2	2395641	2398126	10.74	10.88	10.57	0.3	0.00689	0.0487	Schco3|2611728	
scaffold_1	2487070	2489067	10	10.21	9.74	0.47	0.00693	0.0489	Schco3|2695316	
scaffold_7	1593390	1594841	9.41	9.19	9.6	-0.41	0.00696	0.049	Schco3|2629361	
scaffold_12	628366	630296	10.24	10.44	10.01	0.44	0.00701	0.0493	Schco3|2640524	
scaffold_2	244637	245997	9.46	9.65	9.23	0.42	0.00702	0.0494	Schco3|2487496	
scaffold_10	189267	190145	9.13	9.33	8.89	0.44	0.00705	0.0495	Schco3|2635923	
